# Review and further developments in statistical corrections for Winner’s Curse in genetic association studies

**DOI:** 10.1371/journal.pgen.1010546

**Published:** 2023-09-18

**Authors:** Amanda Forde, Gibran Hemani, John Ferguson

**Affiliations:** 1 School of Mathematical and Statistical Sciences, University of Galway, Galway, Ireland; 2 MRC Integrative Epidemiology Unit, University of Bristol, Oakfield House, Oakfield Grove, Bristol, BS8 2BN, United Kingdom; 3 Population Health Sciences, Bristol Medical School, University of Bristol, Bristol, United Kingdom; 4 HRB Clinical Research Facility, NUI Galway, Galway, Ireland; University College London, UNITED KINGDOM

## Abstract

Genome-wide association studies (GWAS) are commonly used to identify genomic variants that are associated with complex traits, and estimate the magnitude of this association for each variant. However, it has been widely observed that the association estimates of variants tend to be lower in a replication study than in the study that discovered those associations. A phenomenon known as *Winner’s Curse* is responsible for this upward bias present in association estimates of significant variants in the discovery study. We review existing *Winner’s Curse* correction methods which require only GWAS summary statistics in order to make adjustments. In addition, we propose modifications to improve existing methods and propose a novel approach which uses the parametric bootstrap. We evaluate and compare methods, first using a wide variety of simulated data sets and then, using real data sets for three different traits. The metric, estimated mean squared error (MSE) over significant SNPs, was primarily used for method assessment. Our results indicate that widely used conditional likelihood based methods tend to perform poorly. The other considered methods behave much more similarly, with our proposed bootstrap method demonstrating very competitive performance. To complement this review, we have developed an R package, ‘winnerscurse’ which can be used to implement these various *Winner’s Curse* adjustment methods to GWAS summary statistics.

## Author summary

A genome-wide association study is designed to analyse many common genetic variants in thousands of samples and identify which variants are associated with a trait of interest. It provides estimates of association strength for each variant and variants are classified as associated if their test statistics obtained in the study pass a chosen significance threshold. However, due to a phenomenon known as *Winner’s Curse*, the association estimates of these significant variants tend to be upward biased and greater in magnitude than their true values. Naturally, this bias has adverse consequences for downstream statistical techniques which use these estimates. In this paper, we look at current methods which have been designed to combat *Winner’s Curse* and propose modifications to these methods in order to improve performance. Using a wide variety of simulated data sets as well as real data, we perform a thorough evaluation of these methods. We use a metric which allows us to identify which methods, on average, produce adjusted estimates for significant variants that are closest to the true values. To accompany our work, we have created an R package, ‘winnerscurse’, which allows users to easily apply *Winner’s Curse* correction methods to their data sets.

## Introduction

It has been observed that in general, the effect size of a variant or single nucleotide polymorphism (SNP) tends to be lower in a replication study than in the genome-wide association study (GWAS) that discovered the SNP-trait association. This observation is due to the phenomenon known as *Winner’s Curse*. In the context of a single discovery GWAS, the term *Winner’s Curse* describes how the estimates of association strength for SNPs that have been deemed most significant are very likely to be exaggerated compared with their true underlying values. These estimated effect sizes can take the form of log odds ratios (log-OR) resulting from a logistic regression for a binary outcome, e.g. disease status, or regression coefficients (beta) derived from a linear regression for a quantitative trait.

Dudbridge & Newcombe [1] distinguish between the concepts of ranking bias and selection bias, both of which are lenses through which we can view the effect of *Winner’s Curse*. Ranking bias stems from ranking many SNPs, often close to a million or more, by some measure of effect size or statistical significance. In practice, *p*-values are generally used. It is then expected that the bias will be greatest for those variants which have been ranked highly. Selection bias describes how the use of a stringent threshold, such as 5 × 10^−8^, can result in overestimated effect sizes for SNPs that exceed this threshold.

*Winner’s Curse* bias can have many practical consequences, especially with respect to techniques which are reliant on SNP-trait association estimates obtained from GWASs. One such example is Mendelian randomization (MR), a statistical framework which uses genetic variants as instrumental variables to estimate the magnitude of the casual effect of an exposure on an outcome. In the case of two-sample MR, if the same GWAS is used to identify instrument SNPs and estimate their effects relative to the exposure, *Winner’s Curse* will result in the overestimation of these SNP-exposure associations. This bias will then propagate into the causal estimate, resulting in a deflation of this estimate. On the other hand, if instrument SNPs are discovered in the same GWAS as that used to estimate the SNP-outcome associations, the causal estimate will be inflated [2]. In addition, *Winner’s Curse* has been shown to greatly increase the magnitude of weak instrument bias in these MR analyses [3]. Another implication of *Winner’s Curse* bias is in the use of polygenic risk scores which employs GWAS results for prediction purposes. Enlarged association estimates of significant variants used in creating the polygenic score can lead to reduced accuracy in out-of-sample prediction [4].

In this paper, we review existing *Winner’s Curse* correction methods and explore possible modifications that could be made in order to improve some of these methods. However, eliminating this bias induced by *Winner’s Curse* is known to be a difficult task. The challenge in doing so, in terms of genome-wide scans, was first pointed out by Göring et al. [5]. In their paper, they concluded that “attempts at bias correction give unsatisfactory results” and claimed that accurate locus-specific effect size estimates could only be obtained using a sample independent to that used for locus mapping. However, in the absence of readily available replication samples, this recommendation encourages the employment of sample-splitting, in which a portion of the original dataset is withheld for estimation. This approach can have major disadvantages as it reduces the power to detect linkage, whilst also increasing variance in the estimated effect sizes. Fortunately, since this issue was initially highlighted, several methods have been proposed for both linkage and association studies to reduce the bias in large observed effects, without requiring an independent sample.

Sun and Bull [6] are responsible for first proposing the use of the bootstrap to facilitate a solution to the problem of *Winner’s Curse* bias. In their paper, they consider estimating locus-specific effects by using statistical resampling procedures, such as cross-validation and the bootstrap. These techniques result in the original data set being randomly split into a detection and estimation sample, repeatedly. A bias reduction factor can then be generated by comparing the estimates obtained in the detection and estimation samples at each resampling step. Concentrating on the single-locus model, Sun and Bull [6] presented three potential estimators, namely the shrinkage, out-of-sample and weighted estimators, and applied these to the location corresponding to the largest test statistic that also exceeded a genome-wide significance criterion in the original sample. Both simulations and a genome-wide linkage analysis example with an Affected Sib Pair design showed great reduction in bias due to method application, with the bootstrap methods behaving best as judged by smaller MSE. However, for small effect sizes, the bootstrap estimates were still biased upward, which the authors attribute to a form of residual bias.

In the work of Wu et al. [7], additional support was provided for this bootstrap approach. Wu et al. [7] investigated the ability of the three estimators, with respect to reducing the selection bias for effect estimation in locus-specific quantitative trait linkage analysis. Similar to Sun and Bull [6], the methods were used for effect estimation at the location of the maximum test statistic. The results obtained demonstrated the capacity of the estimators to reduce the selection bias of the naïve approach, with their relative effectiveness depending on the size of the true effect and the power to discover it.

In contrast to the above uses of the bootstrap, Jeffries [8] discusses its suitability for the alleviation of bias in multiple comparison settings in general. Jeffries [8] ignores the role that a significance threshold may have in this bias, postulating that for these large observed effect sizes, overestimation is due to the existence of a large random component. In this application of bootstrap techniques to individual-level data, an empirical distribution of overestimation is generated, and utilized to construct alternative confidence intervals. Although the description of the suggested approach is given with respect to differences in gene expression, its potential application to genome-wide scans is recognised. In fact, it is noted that its use is not limited to the highest ranked location, in terms of test statistic, but can also be extended to other highly ranked locations, attenuating the bias described for those effect sizes.

As the popularity of GWASs began to grow, Garner [9] recognised the need to investigate *Winner’s Curse* bias in the context of these association studies. Similar to earlier research, Garner [9] stressed that the lack of success in replicating findings was due to overestimation of the most significant associations, as these estimates were used to elect suitable sample sizes for follow-up studies. Inflation of association estimates is attributed to the selection over many statistical tests and the fact that most studies lack adequate power. Garner [9] attests that increasing sample sizes will result in considerable bias reduction. In addition, in this paper, the asymptotic sampling distribution for the estimated effect size of a SNP after selection using a significance threshold, which is a left-truncated normal distribution, is derived.

A related development was seen in the emergence of *Winner’s Curse* correction methods based on conditional likelihood, such as that of Zöllner and Pritchard [10]. Focussing on case-control studies, Zöllner and Pritchard [10] detail a two-stage computational algorithm which determines the approximate maximum likelihood estimates (MLEs) of the population frequencies of all genotypes for a SNP of interest and corresponding penetrance parameters, conditional on the detection of a significant association signal and known population prevalence of the disease. They demonstrate large reductions in the bias of the parameter estimates, even with a low powered study, although a small degree of downward bias was still observed.

Even though we have chosen the approaches described in Ghosh et al. [11] to represent methods based on conditional likelihood with respect to method evaluation in this manuscript, as discussed below, other notable contributions to the field which alleviate *Winner’s Curse* using conditional likelihood must be mentioned. These include the work of Zhong and Prentice [12], and Xiao and Boehnke [13]. Firstly, Zhong and Prentice [12] focus on a 2-stage GWAS design, in which many SNPs are genotyped in a portion of samples in stage 1, while in stage 2, selected SNPs are genotyped in the remaining samples. For each significant SNP, the 2-stage design can yield a combined estimated effect size, namely the inverse variance weighted log OR. In this context, they propose several bias-reduced estimators, using the asymptotic approximation to the conditional probability density of the estimated log OR given the standardized estimated log OR is greater than a specified cut-point, to derive effect size estimates for selected SNPs. Their best performing estimator, the weighted MSE adjusted estimator, demonstrated nearly unbiased performance, except in very low power settings. However, it was also in these low to moderately powered situations in which all of proposed estimators tended to have greater variance compared with the naïve estimator. In addition, Zhong and Prentice [12] demonstrated that any bias present in the standard error of the estimated effect size of a significant SNP is essentially negligible in comparison to that in the corresponding estimated effect size. The method proposed by Xiao & Boehnke [13] can be considered a simpler version of that of Zöllner and Pritchard [10], which only considers the allele frequency difference and odds ratio parameters. They propose an “ascertainment-corrected” maximum likelihood approach that conditions on observing evidence for association. The conditional likelihood is maximised, as a function of the risk allele frequency in controls and risk allele difference, using the Nelder-Mead simplex technique, to obtain corrected MLEs for both allele frequency difference and odds ratio. With respect to log OR, the corrected MLE typically shows a reduction in both MSE and absolute bias, unless the effect size is large with high power.

On a genome-wide scale, bias correction methods that require only summary data, i.e. regression coefficients and standard errors, have notable advantages compared to methods that require individual-level genotyped data. First, methods based on summary data tend to be more computationally efficient, in terms of run time and memory efficiency. Furthermore, GWAS summary statistics are much more accessible and are more widely used in epidemiological techniques such as MR. Therefore, in this manuscript, we evaluate three more recently proposed methods which require only summary data, as described by Ghosh et al. [11], Ferguson et al. [14] and Bigdeli et al. [15]. These methods are introduced briefly below, and described in technical detail in the ‘Materials and methods’ section.

In a similar manner to the previously discussed condition likelihood methods, Ghosh et al. [11] use an approximate conditional likelihood to provide improved association estimates for SNPs with *p*-values less than the specified genome-wide significance threshold. The problem at hand is reframed as simply the estimation of a single parameter, i.e. the standardized effect size, for a truncated normal distribution. Interestingly, the authors admit that “the conditional MLE has no special optimality properties” in this context. However, their three proposed estimators are shown to greatly improve bias and MSE in the range of small to moderate effect size. Although the method is described in relation to a logistic regression and disease study framework, it can also be used for analyses concerning quantitative traits.

In contrast to the practice above in which correction is performed to each SNP separately, independently of estimated associations of other SNPs, alternative methods have been suggested which involve the use of all SNPs, including those which do not pass the threshold, in order to produce bias-reduced estimated effect sizes. We note that it is only through this joint consideration of all effect sizes that ranking bias can be properly addressed. Thus, the conditional likelihood-based techniques are limited in the sense that they just have the ability to account for the effect of selection bias alone. The empirical Bayes method described by Ferguson et al. [14] uses the collective distribution of all effect sizes to determine a suitable correction for each SNP. The method is shown to be squared error optimal if the true density of observed standardized effect sizes over differing SNPs is known. However, in practice, empirical Bayes needs to estimate the tails of this distribution and as a result, it is possible that rival correction methods can outperform empirical Bayes for larger standardized effects. That said, the empirical Bayes demonstrated superiority in terms of obtaining more accurate association estimates for SNPs with lower effect sizes. Ferguson et al. [14] also note that an additional attractive characteristic of the method is that the correction applied to each SNP is independent of the chosen significance threshold.

Bigdeli et al. [15] suggested the use of False Discovery Rate (FDR) Inverse Quantile Transformation (FIQT) to deal with *Winner’s Curse* bias. Identifying the similarity between correcting for *Winner’s Curse* bias on the scale of standardized effect sizes and adjusting *p*-values for multiple testing, they propose a two-step method. The approach first uses FDR for multiple testing adjustment of SNP *p*-values and then, obtains corrected standardized effect size estimates by means of the Gaussian quantiles corresponding to the adjusted *p*-values. When a sufficient number of signals are present and sample sizes are relatively large, they demonstrate that FIQT has superior MSE compared with competing approaches over a range of simulated scenarios. This performance is credited to the lower variance of FIQT in comparison with other methods.

Returning to the discussion of the evolution of the bootstrap method, Faye et al. [16] considered the extension of the approach beyond application to the most significant locus in a genome-wide linkage setting, as detailed by Sun and Bull [6], and propose a multi-locus bootstrap estimator for use in large-scale GWAS. By implementing identical selection criteria as those imposed on the original data set in each bootstrap sample, the developed method explicitly deals with both ranking and selection bias. Faye et al. [16] also incorporate two modifications in their procedure that aim to reduce overcorrection, especially under low power circumstances. The first takes into account variance differences associated with minor allele frequency while the second removes the negative correlation between the bootstrap and out-of-bootstrap estimates used in determining the bias reduction factor. Even though it was observed that the bootstrap had greater downward bias in comparison to the estimator of Ghosh et al. [11], it consistently produced smaller RMSE values. As this described approach of Faye et al. [16] requires individual-level data, we have chosen to continue the evolvement of the bootstrap in this manuscript by proposing and evaluating an alternative form of the method, which makes corrections using summary statistics.

The concentration of our work detailed here is on techniques which have been designed for use when a replication sample is unavailable. However, we note that there exist methods which use both a discovery and a replication GWAS in order to make suitable corrections to estimated effect sizes of significant SNPs. Examples include the uniformly minimum variance conditionally unbiased estimator (UMVCUE) of Bowden and Dudbridge [17], as well as the conditional likelihood approach of Zhong and Prentice [12], discussed above.

As mentioned above, we have made amendments to existing *Winner’s Curse* correction methods to address certain weaknesses. In particular, we investigated modifications that could be made to the empirical Bayes method in order to ensure that it makes better adjustments to association estimates. Following this review of correction methods, a rigorous evaluation and comparison of these methods was performed. This assessment took place by means of a simulation study as well as engagement with three real data sets. Simulations allowed us to compare methods easily over a wide range of different possible genetic architectures. We then used UK Biobank (UKBB) body mass index (BMI), type 2 diabetes (T2D) and height data sets to see how these techniques would perform in more realistic settings in which a large degree of linkage disequilibrium (LD) exists. In both instances, assessment of methods was predominantly based on the computation of estimated mean squared error (MSE) over significant SNPs. A notable challenge that was encountered at the start of the work discussed in this paper was the lack of available software to implement these various correction methods. Therefore, to complement this review, we have developed an R package, namely ‘winnerscurse’ (https://github.com/amandaforde/winnerscurse), which can be used to apply a number of *Winner’s Curse* adjustment methods to GWAS summary statistics. Techniques which require a replication GWAS are also included in this package.

## Materials and methods

Throughout this paper, we let $Z_{i}=\frac{{\hat{\beta }}_{i}}{\hat{se({\hat{\beta }}_{i})}}$ and $\mu_{i}=\frac{\beta_{i}}{\hat{se({\hat{\beta }}_{i})}}$ with the assumption that

$$Z_{i}∼N(\mu_{i},1)$$ (1)

asymptotically, in which *β*_*i*_ denotes the true effect size of SNP *i* for *i* = 1, …, *N*, ${\hat{\beta }}_{i}$ its estimated effect size, with respect to a trait of interest, and $\hat{se({\hat{\beta }}_{i})}$ its estimated standard error. *N* represents the total number of SNPs in the discovery GWAS. Depending on the type of phenotype, be it a disease or a quantitative trait, this estimated effect size can represent a log odds ratio or a regression coefficient attained from a linear regression, respectively.

### Conditional likelihood

As mentioned, the conditional likelihood method of Ghosh et al. [11] notably differs in its approach at making *Winner’s Curse* corrections from the other methods evaluated in this paper. Adjustments are made to the estimated effect sizes of only those SNPs which satisfy |*z*| > *c*, where *c* is the value corresponding to the pre-specified significance threshold. The reduction in estimated effect size for each significant SNP is imposed independently of other SNPs and is directly determined by the value of *c*. Recognizing that a SNP has been deemed significant, the corresponding conditional likelihood is given by:

$$L_{c}(\mu_{i})=p_{\mu_{i}}(z_{i}||Z_{i}|>c)=\frac{p_{\mu_{i}}(z_{i})}{P_{\mu_{i}}(|Z_{i}|>c)}=\frac{\phi (z_{i}-\mu_{i})}{\Phi (-c+\mu_{i})+\Phi (-c-\mu_{i})}$$ (2)

in which $\phi (x)=\frac{1}{\sqrt{2\pi }}e^{-\frac{x^{2}}{2}}$, the probability density function of the standard Gaussian distribution and $\Phi (x)=\frac{1}{\sqrt{2\pi }}\int_{-\infty }^{x}e^{-t^{2}/2}\;dt$, the corresponding cumulative distribution function (cdf). In general, *c* takes the form of $c=\Phi^{-1}(1-\frac{\alpha }{2})$, with *α* being a threshold to which a Bonferroni correction has been applied in order to control for family-wise error rate, e.g. 5 × 10^−8^.

Using this conditional likelihood, three estimators of *μ*_*i*_, or equivalently of *β*_*i*_ as any estimator for *μ*_*i*_ can be used to produce an estimator for *β*_*i*_ by simply multiplying it by $\hat{\mathrm{se}({\hat{\beta }}_{i})}$, were proposed. The first, ${\tilde{\mu }}_{i1}$ is the obvious conditional maximum likelihood estimator:

$${\tilde{\mu }}_{i1}={\arg\;\max}_{\mu_{i}}L_{c}(\mu_{i})$$ (3)

while the second, ${\tilde{\mu }}_{i2}$ is defined as

$${\tilde{\mu }}_{i2}=\frac{\int_{-\infty }^{\infty }\mu_{i}L_{c}(\mu_{i})d\mu_{i}}{\int_{-\infty }^{\infty }L_{c}(\mu_{i})d\mu_{i}}.$$ (4)

This is the mean of the random variable that follows the distribution *L*_c_(*μ*_*i*_), normalized to ensure a proper density. However, it was observed that for instances in which the true effect size, *β*_*i*_ is close to that of a null effect, the estimator ${\tilde{\mu }}_{i2}$ has greater mean squared error than ${\tilde{\mu }}_{i1}$ but for true effect sizes further from zero, ${\tilde{\mu }}_{i2}$ performs better. Therefore, the use of ${\tilde{\mu }}_{i3}=\frac{{\tilde{\mu }}_{i1}+{\tilde{\mu }}_{i2}}{2},$ which can combine the strengths of these two estimators in order to curtail *Winner’s Curse* bias for significant SNPs more accurately, was suggested.

### Empirical bayes

Motivated by Efron’s empirical Bayes implementation of Tweedie’s formula to correct for selection bias [18], the empirical Bayes method detailed by Ferguson et al. [14] focuses on the importance of sharing information between SNPs in order to make adjustments, through the exploitation of the empirical distribution of all effect sizes. This is a notably different approach to that of the previously discussed conditional likelihood method, which when making a correction to the estimated effect size of a particular SNP essentially fails to acknowledge the existence of any other SNPs.

Under the normal sampling assumption described by Eq [1], Tweedie’s formula describes the relationship between the posterior mean, *E*(*μ*|*z*), and the marginal density function, *p*(*z*), as

$$E(\mu |z)=z+\frac{d}{dz}\log p(z)$$ (5)

Amazingly, provided one can estimate *p*(*z*), Tweedie’s formula facilitates estimation of the posterior mean in complete absence of knowledge of the prior distribution, *π*(*μ*), which in this instance is the true distribution of standardized effect sizes across the genome. Thus, the estimator of *μ*_*i*_ proposed by this method takes the form of

$${\tilde{\mu }}_{i}=\hat{E(\mu_{i}|z_{i})}=z_{i}+\frac{d}{dz_{i}}\log\;\hat{p(z_{i})}.$$ (6)

Estimation of log *p*(*z*) occurs upon application of the following steps. First, partitions of the interval [*z*_1_, *z*_*N*_] of identical width are formed, in which the *z*-statistics have been arranged in ascending order. The number of *z*-statistics which fall inside each partition are noted and regressed against a set of natural cubic spline basis functions with knots located at the midpoint of each partition, using a Poisson generalized linear model. Ferguson et al. [14] suggest choosing the number of basis functions so that the Bayesian Information Criterion (BIC) is minimized for the model. The fitted regression function at *z* is then used to obtain the estimate for log *p*(*z*), and subsequently, ${\tilde{\mu }}_{i}$ for *i* = 1, …, *N*, by means of numerical differentiation.

Ferguson et al. [14] show that if the true marginal density, *p*(*z*), could be used here, then the empirical Bayes estimator would perform optimally at minimizing the mean squared error (MSE) over all SNPs. However, since it is only an estimate of *p*(*z*) that can be obtained, this optimal behaviour is not guaranteed. This is especially a concern in the extreme tails of the distribution where the z-statistics of the most significant SNPs lie as it is more difficult to accurately estimate *p*(*z*) in these regions. Ferguson et al. [14] considered an ad hoc strategy to assist in overcoming this issue. The suggested approach involves the combination of this estimator with the conditional likelihood estimator, in a manner which is determined by the estimators’ respective lengths of 95% confidence and credible intervals. Here, we instead investigated 5 alternative modifications to the original empirical Bayes method described above in order to better stabilize the tail of the estimated marginal density, $\hat{p(z)}$ and its derivative, particularly in the context of strong LD that is observed in high density genotyping arrays. These variations avoid the unappealing combination of two appreciably different estimators, empirical Bayes and conditional likelihood. The explored modifications were:

- Altering the minimum-BIC estimated spline function to be log-linear beyond the 10^th^ largest negative and the 10^th^ largest positive *z*-statistics
- Limiting the number of knots in the spline, in particular using 7 degrees of freedom as originally suggested by Efron [18]
- Utilizing smoothing splines, rather than natural splines, through the gam function in the R package mgcv [19], to avoid specifying knot positions, assuming a poisson distribution for the partition counts
- As above, but this time using a more realistic negative binomial distribution for these counts
- Employing splines with additional shape constraints, through the scam function in the R package scam [20], to enforce monotonicity of the estimated density function, $\hat{p(z)}$

More information about these modifications and their rationale is given in the supplementary material.

### FDR inverse quantile transformation

FDR Inverse Quantile Transformation (FIQT), as proposed by Bigdeli et al. [15], employs a straightforward two-step procedure in order to produce less biased association estimates. First, a FDR (false discovery rate) multiple testing adjustment is applied to the *p*-values of all SNPs, giving FDR adjusted *p*-values *p*_i_*, *i* = 1, …, *N*. Following this, these adjusted *p*-values are transformed back to the *z*-statistic scale by means of an inverse Gaussian cumulative distribution function (cdf) and for each SNP, it is ensured that this new *z*-statistic, $\hat{z_{i}^{*}}$, has the same sign as its original effect size. Mathematically, $\hat{z_{i}^{*}},$ *i* = 1, …, *N*, can be described as

$$\hat{z_{i}^{*}}=\mathrm{sign}(z_{i})\Phi^{-1}(1-\frac{p_{i}^{*}}{2}).$$ (7)

For SNP *i*, its new estimated effect size is simply calculated as $\hat{\beta_{i}}=\hat{z_{i}^{*}}\hat{\mathrm{se}(\hat{\beta_{i}})}$.

The rationale that led to the use of this method is based on the analogy between performing multiple testing adjustments to *p*-values and reducing *Winner’s Curse* bias in estimated SNP effect sizes, in which these effect sizes are in the form of *z*-statistics. In the attempt to correct for *Winner’s Curse*, a shrinkage towards the null effect of zero is generally incurred by the *z*-statistics while the application of a multiple testing adjustment to *p*-values sees the growth of the *p*-values towards one, the null value.

This multiple testing adjustment is imposed through the implementation of the R function p.adjust. This is followed by the use of the R function qnorm for the purpose of back-transformation. However, near zero *p*-values can prove problematic when evaluating qnorm and thus, a restraint is incorporated in FIQT which results in the association estimates of SNPs with very large *z*-statistics, e.g. greater than 37, failing to be adjusted.

### Bootstrap

Inspired by the bootstrap resampling method detailed in Faye et al. [16] and its corresponding computational implementation described by Sun et al. [21], we have established a similar approach which can be easily applied to published sets of GWAS summary statistics without requiring original individual-level data. In addition, a second advantage of our new method is a considerable improvement in computational efficiency over the original method.

This procedure begins with arranging all *N* SNPs according to their original *z*-statistics, $z_{i}=\frac{{\hat{\beta }}_{i}}{\hat{se({\hat{\beta }}_{i})}}$, in descending order, that is a labelling of SNPs is assumed such that *z*_1_ > *z*_2_ > ··· > *z*_*N*_. A randomized estimate of the extent of ranking bias for the *k*^th^ largest *z*-statistic is calculated by means of the parametric bootstrap as follows:

1. A value ${\hat{\beta }}_{i}^{b}$ is simulated for SNP *i*, *i* = 1, …, *N*, independently, from a Gaussian distribution with mean ${\hat{\beta }}_{i}$ and standard deviation $\hat{se({\hat{\beta }}_{i})}$, i.e.
$${\hat{\beta }}_{i}^{b}∼N\left({\hat{\beta }}_{i},\hat{\mathrm{se}({\hat{\beta }}_{i})}\right).$$ (8)
2. Upon obtaining ${\hat{\beta }}_{i}^{b}$ for *i* = 1, …, *N*, the *z*_*i*_^b^-statistic of SNP *i* is defined as
$$z_{i}^{b}=\frac{{\hat{\beta }}_{i}^{b}}{\hat{\mathrm{se}({\hat{\beta }}_{i})}}.$$ (9)
  We define *A*(*k*) as the index corresponding to the *k*^th^ largest entry in the vector: $[z_{1}^{b},\ldots ,z_{N}^{b}]=\left[\frac{{\hat{\beta }}_{1}^{b}}{\hat{\mathrm{se}({\hat{\beta }}_{1})}},\ldots ,\frac{{\hat{\beta }}_{N}^{b}}{\hat{\mathrm{se}({\hat{\beta }}_{N})}}\right]$.
3. Then, the estimated bias of SNP *k*, the SNP with the *k* ^th^ largest original *z*-statistic, takes the following form:
$${\mathrm{bias}}_{k}=\frac{{\hat{\beta }}_{A(k)}^{b}-{\hat{\beta }}_{A(k)}^{\mathrm{oob}}}{\hat{\mathrm{se}({\hat{\beta }}_{A(k)})}}=\frac{{\hat{\beta }}_{A(k)}^{b}-{\hat{\beta }}_{A(k)}}{\hat{\mathrm{se}({\hat{\beta }}_{A(k)})}},$$ (10)
  in which ${\hat{\beta }}_{A(k)}^{b}$ is the bootstrap value of the SNP ranked in position *k* in the ordering of *z*_*i*_^b^-statistics, ${\hat{\beta }}_{A(k)}^{\mathrm{oob}}={\hat{\beta }}_{A(k)}$ is that same SNP’s original *β* estimate and $\hat{\mathrm{se}({\hat{\beta }}_{A(k)})}$ its standard error.

In the next step of the process, a cubic smoothing spline is fitted to the data in which the *z*-statistics are considered as the inputs and bias_*k*_, their corresponding outputs. The predicted values from this model fitting provides new estimates for the bias correction, bias_k_* for each SNP. This additional stage in which bias_k_* is obtained reduces the need for several bootstrap iterations for each SNP in order to ensure competitive performance of the method. Thus, the use of the cubic smoothing spline results in an approach that is faster as only one bootstrap iteration is required per SNP, while using bias_k_* instead of bias_*k*_ ensures that the method has greater accuracy. Finally, the new estimate for the true effect size of SNP *k*, the SNP with the *k*^th^ largest original *z*-statistic, is defined as: ${\hat{\beta }}_{k}^{*}={\hat{\beta }}_{k}-\hat{\mathrm{se}({\hat{\beta }}_{k})}∙{\mathrm{bias}}_{k}^{*}$.

We note that our code for this method includes the following two features. Firstly, in order to avoid the sign of ${\hat{\beta }}_{k}^{*}$ being different to that of ${\hat{\beta }}_{k}$, if the method results in a change of sign then we simply set ${\hat{\beta }}_{k}^{*}$ = 0. Secondly, due to the nature of *Winner’s Curse* bias, it is desirable that our method would only result in reductions in the absolute estimated effect sizes of significant SNPs, i.e. $\left|{\hat{\beta }}_{k}^{*}|\leq |{\hat{\beta }}_{k}\right|$. Therefore, if the bootstrap approach described results in the adjusted estimated effect size of a SNP being greater in absolute value than its corresponding original estimate, then we fail to adjust the estimate and set ${\hat{\beta }}_{k}^{*}={\hat{\beta }}_{k}$. With respect to significant SNPs, we have seen in practice that situations in which either of these lines of code must be employed are in fact extremely rare.

In addition to those mentioned previously, there are several notable differences between our algorithm described above and the method proposed by Faye et al. [16]. Firstly, it is the parametric bootstrap that is used here to estimate the magnitude of bias for each SNP as opposed to the more common nonparametric bootstrap which requires individual-level data. Our method draws only one bootstrap resample, i.e. only one bootstrap value ${\hat{\beta }}_{i}^{b}$ is simulated for SNP *i*, *i* = 1, …, *N*. It also includes an extra step which involves the use of a smoothing spline. In contrast, Faye et al. [16] express the need for a number of bootstrap samples, e.g. at least 100, in their approach. Furthermore, Faye et al. [16] correct the out-of-bootstrap estimator for a particular SNP in each bootstrap sample, ${\hat{\beta }}_{E}$, to ensure that it is uncorrelated with the bootstrap estimate, ${\hat{\beta }}_{D}$, conditional on the estimate, ${\hat{\beta }}_{N}$, in the entire data set. Note that ${\hat{\beta }}_{E}$, ${\hat{\beta }}_{D}$ and ${\hat{\beta }}_{N}$ are notation used in Faye et al. [16]. In the algorithm described above, this is unnecessary as our bootstrap estimates, ${\hat{\beta }}_{A(k)}^{b}$, as defined above, are simulated at random and thus, by definition are independent of ${\hat{\beta }}_{A(k)}^{\mathrm{oob}}$, conditional on the estimates ${\hat{\beta }}_{A(k)}^{\mathrm{oob}}={\hat{\beta }}_{A(k)}$. A final difference is that the algorithm in Faye et al. [16] only uses bootstrap resamples where the *k*^th^ most significant SNP was above the significance threshold when calculating the bias correction for the *k*^th^ most significant SNP on the overall sample. Our bootstrap procedure uses all available parametric bootstrap resamples, which is by default only one, in calculating the bias correction.

### Simulation study

The simulation study followed a factorial design in which GWAS summary statistics were simulated for a quantitative trait under 8 different genetic architectures, described by combinations of three parameters, namely sample size *n*, heritability *h*^2^, polygenicity (proportion of effect SNPs) *π*. The following the values chosen for these parameters:

- sample size *n*∈{30000,300000}
- heritability *h*^2^∈{0.3,0.8}
- polygenicity *π*∈{0.01,0.001}

Assuming a normal distribution of effect sizes, for a fixed array of *N* = 1,000,000 SNPs, our strategy entailed imposing a simple correlation structure on the SNPs in order to imitate the presence of linkage disequilibrium (LD) in real data. It was assumed that the same correlation structure exists in independent blocks of 100 SNPs. Thus, for each block of 100 SNPs, the estimated effect sizes, ${\hat{\beta }}_{i}$ were simulated using:

$$\hat{\beta }∼N(D^{-\frac{1}{2}}RD^{\frac{1}{2}}b,D^{-\frac{1}{2}}RD^{-\frac{1}{2}}\sigma^{2}).$$ (11)

Here, ***b*** is a vector containing the true SNP-trait effect sizes which have been scaled to ensure that the phenotype has variance 1. The matrix ***D*** is a diagonal 100 × 100 matrix, in which *d*_*i*_ = *n*∙2∙maf_*i*_(1−maf_*i*_) and maf_*i*_ is the minor allele frequency of SNP *i*, while ***R*** is a simple 100 × 100 matrix of inter-genotype correlations, with $R_{ij}={\hat{\rho }}^{\left|i-j\right|}$ and $\hat{\rho }=0.9825$. The reasoning for the selection of this value for $\hat{\rho }$ and why it was considered suitable, as well as other details regarding this simulation, are described in the supplementary material. For each SNP, values for ${\hat{\beta }}_{i},\;\hat{se({\hat{\beta }}_{i})}$ and $E({\hat{\beta }}_{i})$ were produced with $E({\hat{\beta }}_{i})$ obtained using $E(\hat{\beta })=D^{-\frac{1}{2}}RD^{\frac{1}{2}}b$. For each of these 8 different genetic architectures, 100 sets of summary statistics were simulated.

The *Winner’s Curse* correction methods detailed in ‘Materials and methods’ were applied to each data set using the R package ‘winnerscurse’, producing adjusted estimated effect sizes, ${\hat{\beta }}_{\mathrm{adj},i}$, for each SNP *i*, *i* = 1, …, *N*. The performance of these methods were investigated at two different significance thresholds, namely *α*_1_ = 5 × 10^−8^ and *α*_2_ = 5 × 10^−4^, with a stronger focus given to the more commonly used genome-wide significance threshold of *α*_1_ = 5 × 10^−8^. In order to assess each method’s ability at providing less biased SNP-trait association estimates, the estimated change in root mean squared error (RMSE) of significant SNPs due to method implementation were computed for each data set and method. For simplicity, let *i* = 1, …, *N*_sig_ represent indexes for the significant SNPs in a particular simulated set of summary statistics, i.e. *N*_sig_ is the number of SNPs which satisfy |*z*_*i*_| > *c* with $\left|z_{i}|=\left|\frac{{\hat{\beta }}_{i}}{\hat{\mathrm{se}({\hat{\beta }}_{i})}}\right|,\;c=\Phi^{-1}(1-\frac{\alpha }{2}\right)$ and *α*∈{*α*_1_, *α*_2_}. Then, the estimated change in RMSE of significant SNPs may be defined as:

$$\sqrt{\frac{1}{N_{\mathrm{sig}}}\sum_{i=1}^{N_{\mathrm{sig}}}{\left({\hat{\beta }}_{\mathrm{adj},i}-\beta_{i}\right)}^{2}}-\sqrt{\frac{1}{N_{\mathrm{sig}}}\sum_{i=1}^{N_{\mathrm{sig}}}{\left({\hat{\beta }}_{i}-\beta_{i}\right)}^{2}}.$$ (12)

The change in RMSE for each method was calculated for only those data sets in which at least one significant SNP was detected. This evaluation metric based on RMSE was considered to be most suitable for several reasons. Firstly, by focussing on bias alone, it is possible that we may recommend methods that have high variability, and which if judged instead by metrics like MSE or RMSE would be regarded as inaccurate. Bias correction will usually increase standard errors and the extent of this increase is an important factor to consider when recommending a method. Due to our approach to simulation, we cannot compute standard errors conditional on selection as we simulate differing true effects for each SNP on differing simulation iterates. Therefore, empirical RMSE seems a good metric to focus on that naturally encompasses both bias and standard error, conditional on selection, into one quantity. In addition, RMSE has the advantage over MSE that it is measured on the scale of the true effects, rather than a squared scale, and therefore, is more interpretable. In addition to the above metric, the relative change in RMSE, which is equal to the change in RMSE divided by the naïve RMSE, was computed in a similar manner. For a given correction method, this value provides the percentage improvement in RMSE due to applying that method to the set of summary statistics. The final metric computed was the estimated average bias over all significant SNPs, defined as:

$$\frac{1}{N_{\mathrm{sig}}}\sum_{i=1}^{N_{\mathrm{sig}}}\left({\hat{\beta }}_{\mathrm{adj},i}-\beta_{i}\right).$$ (13)

It was required to compute this average bias separately for significant SNPs with positive and negative true effects to avoid the bias in either direction simply cancelling each other out.

In addition to the above simulation set-up, GWAS summary statistics were simulated and methods evaluated under the assumption that SNPs were independent. In this instance, 100 sets of summary statistics were simulated for each combination of parameters, while the evaluation metric concerning the change in RMSE of significant SNPs was prioritised. This simulation process, which incorporates an independence assumption, was repeated in a similar fashion for a binary trait with a normal distribution of effect sizes. Furthermore, a quantitative phenotype with a bimodal effect size distribution as well as one with a skewed distribution were also considered. Further details regarding these additional simulations can be found in the supplementary material.

### Empirical analysis

In order to compare the performance of these *Winner’s Curse* correction methods with respect to real data, three different UK Biobank data sets were used, namely body mass index (BMI), height and type 2 diabetes (T2D). As with real data, the true effect size of each SNP is unknown and so it is more difficult to assess how much each method reduces the bias induced by *Winner’s Curse*. To overcome this challenge, each original large data set was randomly split in two, leaving between 166,172 and 166,687 individuals in each of the six smaller data sets. This provided the ability to execute two independent GWASs of similar sample size for each trait in which one GWAS was designated as the discovery GWAS and the other the replication GWAS. The unbiased replication GWAS association estimates can then be used as proxies for the true effect sizes of the SNPs found to be significant in the discovery GWAS. PLINK 2.0 [22] was used to perform quality control as well as each of the statistical analyses.

The genotypic data used was collected, processed and imputed by UK Biobank (UKBB) [23]. During our implementation of required quality control steps, we ensured the removal of both related individuals and those that were of non-European ancestry. These non-European samples were identified by principal component analysis (PCA) using 1000 Genomes Project (1KGP) data. This was done by first computing the multi-mean of the top eight principal components for 1KGP samples of European ancestry. The UKBB samples with a Mahalanobis distance >6 SD from this multi-mean were then considered to be non-European and subsequently removed from our data set. The UKBB provided relatedness file allowed us to easily remove related samples. This file lists pairs of individuals related up to the third degree and thus, one individual from each pair was removed from the data set until no related individuals remained. Furthermore, samples which had been identified as outliers with respect to heterozygosity and missingness, together with samples with discordant sex information and those suffering from chromosomal aneuploidy, were also discarded. These particular samples were listed in the UKBB supplied sample quality control file. The total number of samples remaining after the execution of these quality control steps were 332,618, 333,642 and 332,927 for BMI, T2D and height, respectively. With respect to variants, only those with an information score greater than 0.8, a minor allele frequency greater than 0.01, a genotyping rate of at least 98% and those that passed the Hardy-Weinberg test at the specified significance threshold of 1 × 10^−8^ were included. This resulted in a total of 7,915,560 SNPs that were considered suitable for our analyses.

The methods of interest were applied to the summary statistics of each discovery GWAS using the R package ‘winnerscurse’. Evaluation took place by computing the estimated MSE of *N*_sig_ significant SNPs in that GWAS, defined as:

$$\frac{1}{N_{\mathrm{sig}}}\sum_{i=1}^{N_{\mathrm{sig}}}{\left({\hat{\beta }}_{\mathrm{disc},\mathrm{adj},i}-{\hat{\beta }}_{\mathrm{rep},i}\right)}^{2}-\frac{1}{N_{\mathrm{sig}}}\sum_{i=1}^{N_{\mathrm{sig}}}{\left(\hat{\mathrm{se}({\hat{\beta }}_{\mathrm{rep},i})}\right)}^{2}.$$ (14)

The subtraction of the average standard error in the above expression ensures that the computed metric approximates the true empirical MSE of significant SNPs. A detailed derivation of Eq [14] is provided in the supplementary material. It is possible that this estimated MSE quantity may be negative in some instances. Despite this, comparison of this metric across differing methods is still considered meaningful with lower values of estimated MSE being the most desirable. Much like the simulation study, the average bias was also calculated for both positive and negative significant SNPs, with a similar definition to Eq [13] but in which the true effect size, *β*_*i*_, is replaced by the estimated effect size obtained in the replication study. For each of the three traits, it was possible to evaluate the performance of methods twice as in each case, the original roles of the two independent data sets, i.e. discovery and replication, could be switched and re-evaluation of methods could then take place with respect to the SNPs that were deemed significant in this new discovery GWAS.

## Results

### Simulation study

#### When is winner’s curse bias most prominent?

A simulation study in which a simple correlation structure was imposed on the set of *N* = 1,000,000 SNPs was first executed, as described in ‘Materials and methods’. Before application of the *Winner’s Curse* correction methods to the sets of summary statistics, an attempt to gain an insight into the simulation scenarios in which *Winner’s Curse* bias is most prominent was made. This was done by computing the average number of significant SNPs, the average naïve MSE of significant SNPs and the average proportion of significant SNPs that had significantly overestimated effect sizes in each setting with respect to two significance thresholds, 5 × 10^−8^ and 5 × 10^−4^. A SNP is defined as being significantly overestimated or as having a significantly more extreme effect size estimate, at the 2.5% significance level, if it satisfies the condition:

$$|{\hat{\beta }}_{i}|>|\beta_{i}|+1.96∙\hat{\mathrm{se}({\hat{\beta }}_{i})}$$ (15)

Thus, the proportion of significant SNPs that are significantly overestimated is considered to be representative of the proportion of significant SNPs with effect size estimates that greatly suffer from *Winner’s Curse* bias. As detailed in S1 Table, it was clear that as sample size was increased from 30,000 to 300,000, this proportion of significant SNPs decreased. The other two parameters which played key roles in defining the various simulated genetic architectures were heritability and polygenicity. It was observed that the proportion of significantly overestimated significant SNPs decreased when heritability was increased from 0.3 to 0.8, but when the value representing trait polygenicity was increased from 0.001 to 0.01, this proportion increased.

In fact, it was also noted that as the number of significant SNPs increased, both the MSE of significant SNPs and the proportion of these that were significantly overestimated decreased. This can be clearly seen in S1 Fig. This indicates that as the number of samples in a study and as the number of SNPs passing the significance threshold increases, bias induced by *Winner’s Curse* will be less of an issue among significant SNPs. In terms of genetic architecture characteristics, these results suggest that the presence of *Winner’s Curse* bias in the estimated effect sizes of significant SNPs should be of a greater concern when investigating traits with lower heritability or traits which have a larger proportion of effect SNPs. Of course, these factors mentioned here all directly pertain to the power of genome-wide significance of true effects, which is likely the most important one variable summary of the degree of *Winner’s Curse*, as shown in much of the existing literature. The extent of *Winner’s Curse* bias will partially depend on the true effect size distribution. This true effect size distribution is dependent on a combination of factors such as polygenicity, heritability and allele frequency distribution, with all of these features relating to power and in turn, determining the number of SNPs passing the significance threshold. In addition, increasing the sample size will result in increased power and less *Winner’s Curse* bias. However, differing combinations of polygenicity, heritability, allele frequency distribution and sample size can generate the same distribution of power, and thus it is worth taking into account how these other factors jointly affect the degree of *Winner’s Curse* bias.

#### Evaluation of performance at *p <* 5 × 10^−8^

**Fig 1:**
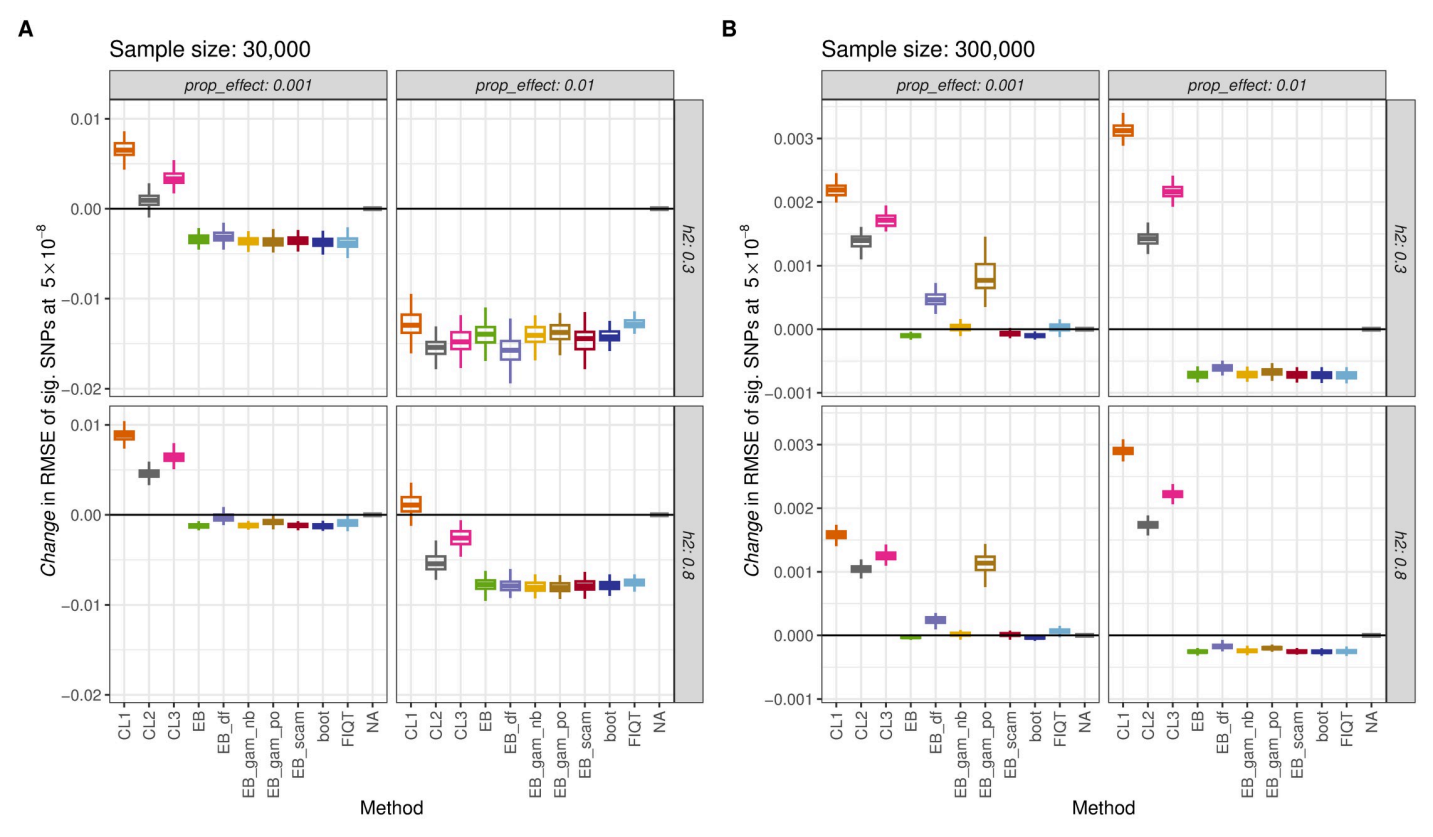
Estimated change in RMSE of significant SNPs at threshold 5 × 10^−8^ for each method and simulation setting, with a simple correlation structure imposed on the set of SNPs. RMSE is evaluated for 100 simulated sets of summary statistics for each setting. (A) contains results for sample sizes of 30,000 while (B) concerns sample sizes of 300,000. Heritability is denoted by ‘h2’ while ‘prop_effect’ represents the proportion of effect SNPs. Methods are abbreviated as: ‘CL1’,’CL2’ and ‘CL3’ = conditional likelihood estimators of Ghosh et al. [11], ‘EB’ = original empirical Bayes method with tail restriction, ‘EB_df’ = empirical Bayes with fixed degrees of freedom of 7, ‘EB_gam_po’ = empirical Bayes with smoothing splines assuming poisson count distribution, ‘EB_gam_nb’ = empirical Bayes with smoothing splines assuming negative binomial count distribution, ‘EB_scam’ = empirical Bayes with shape constrained additive models, ‘boot’ = proposed bootstrap approach and ‘FIQT’ = FDR Inverse Quantile Transformation. The solid black lines represent no change in RMSE.

With respect to the evaluation of methods, we focus on the results of computing the quantity ‘change in RMSE over all significant SNPs due to method implementation’ for each method, with obtaining a negative value being desirable. These results are provided in S2 and S6 Tables. At a threshold of 5 × 10^−8^, several observations were notable. Firstly, for scenarios in which sample size has been designated the greater value of 300,000, the effect of applying the methods is on a much smaller scale to those scenarios with sample sizes of 30,000. This is evident from the large difference in the values on the y-axis between plots (A) and (B) of Fig 1. This observation ties in with the fact that the magnitude of *Winner’s Curse* bias is greater at *n* = 30,000. At this 5 × 10^−8^ significance threshold, the conditional likelihood methods are seen to perform poorly, especially when sample sizes are increased to 300,000. In most instances, these methods provide worse association estimates than the naïve approach, often increasing the RMSE. In addition to an increase in variance, the reason for this observation is the overcorrection of estimated effect sizes, especially those that lie close to the significance threshold. See the supplementary material for further discussion.

The novel bootstrap method is one of the most consistent methods at reducing the RMSE of association estimates for significant SNPs at this threshold. In all situations depicted, it has one of the largest negative values for the change in RMSE of significant SNPs and on average, improves the RMSE by 15% across the 8 settings, as shown in S3 Table. FIQT tends to perform in a somewhat similar manner to this bootstrap method. With respect to the empirical Bayes method and its variations, for sample sizes of 300,000, the best performing versions were the original empirical Bayes method (‘EB’), ‘EB-gam-nb’ and ‘EB-scam’. With the lower sample size, all variations perform very similarly. However, the empirical Bayes variants ‘EB-df’ and ‘EB-gam-po’ performed less well overall. In the case of ‘EB-gam-po’, this might be partly due to convergence problems that sometimes occurred in obtaining the poisson regression fit. In general, we advise caution in utilizing the empirical Bayes results in the context of convergence warnings from R.

In terms of the average bias over all significant SNPs at this 5 × 10^−8^ threshold, the results obtained may be viewed in S3 and S4 Figs, as well as S4 and S5 Tables. As expected, due to the symmetric nature of the simulated effect size distributions, the values computed for the average bias of positive significant SNPs are essentially mirrored by the negative significant SNPs. The plots produced for sample sizes of 30,000 and proportion of effect SNPs of 0.01 provide support for the use of the conditional likelihood methods. However, their tendency to overcorrect the estimated effect sizes is evident as negative values for the average bias of positive SNPs are witnessed regularly. Similar to above, it is deduced that the most consistent performances at producing average bias values close to zero are demonstrated by the empirical Bayes versions ‘EB’, ‘EB-gam-nb’, ‘EB-scam’, the bootstrap method and FIQT.

#### Evaluation of performance at *p* < 5 × 10^−4^

A lower threshold of 5 × 10^−4^ was also investigated. Less emphasis is placed on the results obtained at this threshold as it is possible that many false positives are detected here, i.e. many SNPs that in fact have a true effect size of zero pass the significance threshold, although lower thresholds may be useful for the construction of polygenic risk scores. Therefore, as these *Winner’s Curse* correction methods are all considered to be shrinkage methods, improvements in the RMSE over all significant SNPs would be expected. However, as can be seen in S5 Fig, positive values are witnessed at this threshold when sample sizes are large. However, these positive values most often occur for the conditional likelihood methods which seem to be the worst performers overall. It seems here that the most consistent and best performing methods are the bootstrap method, the original empirical Bayes method and the empirical Bayes method which uses shape constrained additive models (SCAMs). These methods all reduce the RMSE of significant SNPs at this threshold by an average of at least 18.7%, as shown in S7 Table.

#### Additional simulations in absence of linkage disequilibrium

In order to demonstrate potential performance of the *Winner’s Curse* methods in the context of SNP-trait associations from genome-wide arrays with lower SNP density or LD-pruned datasets, we also examined the less complex situation in which SNPs are independent. The results of these extra simulations are shown in S8–S15 Figs, and described in depth in the supplementary material. In this setting, with a normal effect size distribution, the most consistent methods in terms of reducing the RMSE of significant SNPs were the original empirical Bayes method and ‘EB-gam-nb’. Just as was observed in the simulations with linkage disequilibrium, the conditional likelihood methods perform poorly and often result in an increase in the evaluation metric in comparison to the naïve approach, while the proposed bootstrap method continued to exhibit competitive performance.

### Empirical analysis

The results of an initial exploration of the six UK Biobank sets of summary statistics are detailed in Table 1. From trait to trait, there is a large difference in the number of SNPs with *p*-values lower than the genome-wide significance threshold of 5 × 10^−8^. Values for the proportion of these SNPs with significantly overestimated effect sizes in each discovery GWAS are included. A comparison of BMI and height GWASs at the 5 × 10^−8^ threshold tends to indicate that as the number of significant SNPs increases, the proportion that are significantly overestimated decreases. This trend is even more apparent at the larger threshold of 5 × 10^−4^, and as stated above, was also clearly observed in the simulated data.

**Table 1:** The number of significant SNPs at two significance thresholds, 5 × 10^−8^ and 5 × 10^−4^, with proportions that indicate the extent of *Winner’s Curse* bias for each data set.

GWAS	No. sig. SNPs (5 × 10^−8^)	Prop. sig. SNPs with *smaller* replication estimate (5 × 10^−8^)	Prop. sig. SNPs *significantly* overestimated (5 × 10^−8^)	No. sig. SNPs (5 × 10^−4^)	Prop. sig. SNPs with *smaller* replication estimate (5 × 10^−4^)	Prop. sig. SNPs *significantly* overestimated (5 × 10^−4^)
**BMI 1**	6,908	0.7135	0.2251	94,173	0.8365	0.3386
**BMI 2**	7,951	0.8009	0.3089	98,351	0.8455	0.3604
**T2D 1**	31	0.0645	0.0645	5,832	0.9830	0.8433
**T2D 2**	76	1	0.1579	5,507	0.9951	0.8397
**Height 1**	70,020	0.6444	0.1829	257,000	0.6940	0.2095
**Height 2**	70,634	0.6825	0.1772	268,497	0.7179	0.2406

As well as the number of significant SNPs, Table 1 contains the proportion of these significant SNPs that had smaller estimated effect sizes, in terms of absolute value, in their respective replication GWAS, and the proportion of significant SNPs that have significantly overestimated effect sizes, for each data set.

#### The problem of Linkage Disequilibrium in real data

Naturally, the results of engagement with real data sets are more complex than those of the simulation study. For example, it was noted that for BMI, in one instance, all significant SNPs which had a *z*-value greater than 15 in the discovery GWAS had association estimates in the replication GWAS which were in fact greater. This observation can be clearly seen in S16 Fig in which *z*-statistics are plotted against estimated bias for each data set, with estimated bias of SNP *i* defined as:

$$\hat{{\mathrm{bias}}_{i}}={\hat{\beta }}_{\mathrm{disc},i}-{\hat{\beta }}_{\mathrm{rep},i}$$ (16)

This finding is of course contrary to what is expected. However, these SNPs with *z*-values greater than 15 were all in strong linkage disequilibrium and thus, represented a single independent signal. It can be seen in Table 1 that a similar result was noted when the first T2D data set was used as the discovery GWAS. When using a significance threshold of 5 × 10^−8^, most of the 31 significant SNPs had larger estimated effect sizes in the replication GWAS than in the discovery GWAS. In these cases, we need to be careful not to over-generalize or interpret the results of applying a *Winner’s Curse* correction, given that there may be very few independent association signals at *p* < 5 × 10^−8^.

#### Evaluation of performance at *p* < 5 × 10^−8^

**Fig 2:**
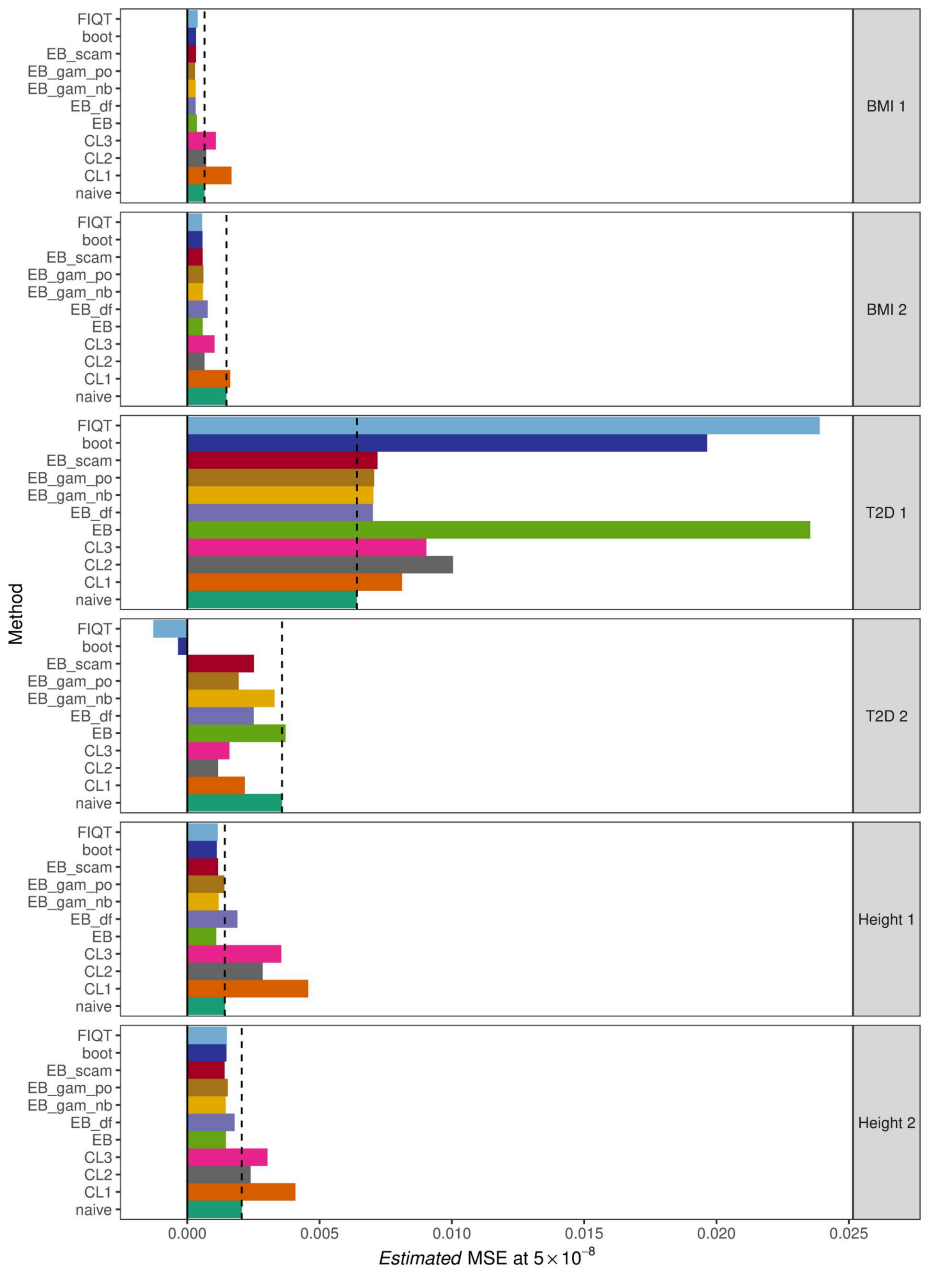
Estimated MSE of significant SNPs at threshold 5 × 10^−8^ for each method and data set. Methods are abbreviated as: ‘naïve’ = no correction method applied, ‘CL1’,’CL2’ and ‘CL3’ = conditional likelihood estimators of Ghosh et al. (11), ‘EB’ = original empirical Bayes method with tail restriction, ‘EB_df’ = empirical Bayes with fixed degrees of freedom of 7, ‘EB_gam_po’ = empirical Bayes with smoothing splines assuming poisson count distribution, ‘EB_gam_nb’ = empirical Bayes with smoothing splines assuming negative binomial count distribution, ‘EB_scam’ = empirical Bayes with shape constrained additive models, ‘boot’ = proposed bootstrap approach, and ‘FIQT’ = FDR Inverse Quantile Transformation. The darker green bar and dashed black line represent the estimated MSE of the naïve approach.

As stated in ‘Materials and methods’, the methods were evaluated using the estimated MSE of SNPs which passed the chosen significance threshold. Using the threshold of 5 × 10^−8^, the estimated MSE for each method and GWAS combination are displayed in Table 2 while Fig 2 provides a corresponding illustration of these values. In this figure, the light blue bar as well as the black dotted horizontal line mark the estimated MSE obtained using the naïve approach, i.e. when no *Winner’s Curse* correction method has been applied and the raw effect estimates are used. This provides a standard to which the performance of each method can be directly compared with, in which it is desired that method application will result in an estimated MSE less than this approach. Similar to the section above describing the results of the simulation study, the poor performance of the conditional likelihood methods is evident. In 5 out of the 6 independent instances, it was observed that at least one of these methods had a greater estimated MSE than that of the naïve approach.

**Table 2:** Estimated MSE of significant SNPs at threshold 5 × 10^−8^ for each method and data set.

GWAS	BMI 1	BMI 2	T2D 1	T2D 2	Height 1	Height 2
**naive**	0.00065	0.00148	0.00641	0.00358	0.00142	0.00206
**CL1**	0.00167	0.00162	0.00811	0.00217	0.00457	0.00408
**CL2**	0.00071	0.00065	0.01004	0.00116	0.00285	0.00239
**CL3**	0.00108	0.00103	0.00903	0.00159	0.00355	0.00303
**EB**	0.00036	0.00058	0.02354	0.00371	0.00109	0.00146
**EB df = 7**	0.00031	0.00077	0.00701	0.00251	0.00189	0.00179
**EB scam**	0.00033	0.00058	0.00719	0.00252	0.00116	0.00141
**EB gam-po**	0.00029	0.00061	0.00706	0.00194	0.0014	0.00153
**EB gam-nb**	0.00031	0.00059	0.00703	0.0033	0.00118	0.00145
**boot**	0.00033	0.00057	0.01964	-0.0004	0.00111	0.00148
**FIQT**	0.00039	0.00056	0.0239	-0.0013	0.00115	0.00149

Methods are abbreviated as: ‘naïve’ = no correction method applied, ‘CL1’,’CL2’ and ‘CL3’ = conditional likelihood estimators of Ghosh et al. (11), ‘EB’ = original empirical Bayes method with tail restriction, ‘EB df = 7’ = empirical Bayes with fixed degrees of freedom of 7, ‘EB scam’ = empirical Bayes with shape constrained additive models, ‘EB gam-po’ = empirical Bayes with smoothing splines assuming poisson count distribution, ‘EB gam-nb’ = empirical Bayes with smoothing splines assuming negative binomial count distribution, ‘boot’ = proposed bootstrap approach, and ‘FIQT’ = FDR Inverse Quantile Transformation. Values greater than their corresponding naïve value are shaded in grey while light green shaded cells highlight the method that resulted in the lowest estimated MSE value for each data set.

On real data, the original empirical Bayes method proposed by Ferguson et al. [14] performed poorly, sometimes failing to adjust estimated associations downwards. This observation motivated the proposal of possible modifications as mentioned in ‘Materials and methods’. These suggested improvements, in particular the inclusion of the shape-constrained additive models and the use of the generalized additive model, resulted in slightly more consistent reductions in MSE over significant SNPs. In fact, taking all six data sets into account, it is ‘EB-scam’ and ‘EB-gam-po’, which tend to be the best performing methods, having an average improvement on estimated MSE of greater than 29.4% over the naïve approach.

However, as stated previously, we must be cautious when using the first T2D data set to evaluate methods, and also when using the second T2D data set. The problem with linkage disequilibrium and very few independent signals is common to both data sets. In Fig 2 for the first T2D data set, it is witnessed that all methods result in greater estimated MSE values than the naïve approach, with the original empirical Bayes method, bootstrap and FIQT clearly greatly shrinking the estimated effect sizes of significant SNPs away from those larger replication effect sizes. Therefore, if we exclude these two T2D data sets and re-compute the average improvement in estimated MSE for each method, it is our proposed bootstrap method which is seen to be the dominant method with an average improvement of approximately 40.2%.

Given the issues regarding method performance that occur in the presence of linkage disequilibrium, we also acquired a pruned set of SNPs and investigated the behaviour of the correction methods in that setting. The results obtained are provided in S15 and S16 Tables, and illustrated in S21 and S22 Figs, with further details included in the supplementary material. Comparing these results with those attained for the original set of SNPs, a large degree of similarity is evident. The single most notable difference is seen at the 5 × 10^−8^ threshold for the T2D datasets, in which most methods are now producing a lower estimated MSE than that of the naïve approach. However, it must be noted that these calculations are based on only two and four significant SNPs for the first and second T2D data sets, respectively. At this 5 × 10^−8^ threshold, using the pruned set of SNPs, ‘EB’, ‘EB-gam-nb’ and FIQT were the most reliable correction methods, producing an average improvement in estimated MSE of greater than 50% across the six data sets.

Applying the methods to the T2D data sets highlighted the importance of having a sufficient number of independent signals in the set of significant SNPs, in order to ensure appropriate method performance. This observation motivated a brief empirical investigation, which aimed to establish how many independent signals are required so that these methods perform better than the naïve approach of using no correction. Our work, detailed in the supplementary material, suggests that one would need at least 30 independent significant signals to guarantee confidence in the application of the empirical Bayes methods, the bootstrap method or FIQT.

In addition to the above, we also considered the average bias over all positive and negative significant SNPs at the 5 × 10^−8^ threshold, as shown in S17 and S18 Figs. We note that both T2D data sets had zero significant SNPs with negative estimated effect sizes at this threshold. The results obtained here for the height and BMI data sets further emphasize the overcorrection tendency of the conditional likelihood approaches, as demonstrated by large positive values for the average bias of negative SNPs. In most instances, the proposed bootstrap method, ‘EB’ and ‘EB-scam’ yield small values for the average bias closer to zero than that of the naïve approach.

#### Evaluation of performance at *p* < 5 × 10^−4^

**Fig 3:**
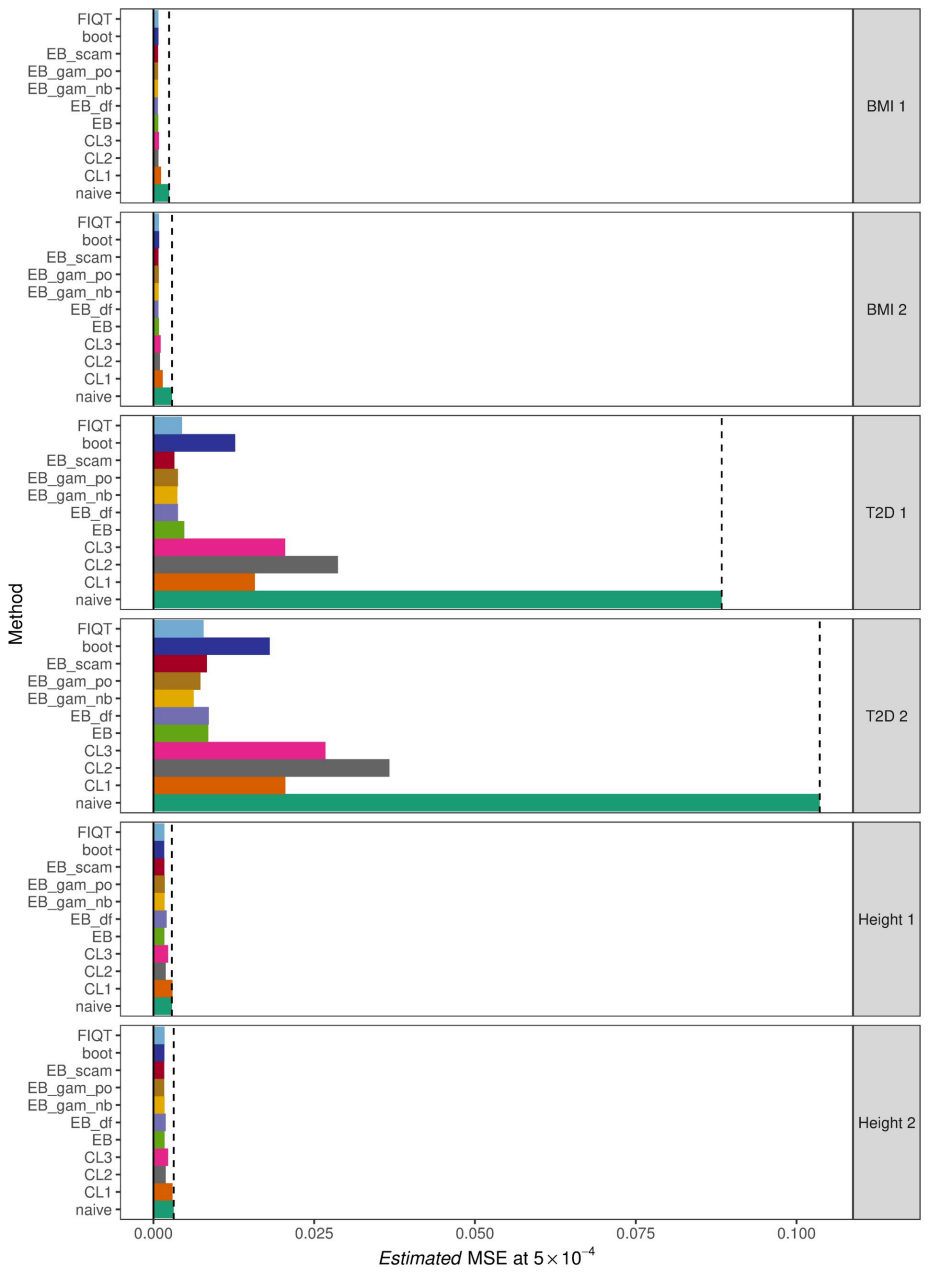
Estimated MSE of significant SNPs at threshold 5 × 10^−4^ for each method and data set. Methods are abbreviated as: ‘naïve’ = no correction method applied, ‘CL1’,’CL2’ and ‘CL3’ = conditional likelihood estimators of Ghosh et al. [11], ‘EB’ = original empirical Bayes method with tail restriction, ‘EB_df’ = empirical Bayes with fixed degrees of freedom of 7, ‘EB_gam_po’ = empirical Bayes with smoothing splines assuming poisson count distribution, ‘EB_gam_nb’ = empirical Bayes with smoothing splines assuming negative binomial count distribution, ‘EB_scam’ = empirical Bayes with shape constrained additive models, ‘boot’ = proposed bootstrap approach, and ‘FIQT’ = FDR Inverse Quantile Transformation. The darker green bar and dashed black line represent the estimated MSE of the naïve approach.

This evaluation procedure was repeated using a larger significance threshold of 5 × 10^−4^. The results of which can be found summarised in S12 Table and Fig 3. At this threshold, for all 6 data sets, all of the methods produce estimated MSE values less than the naïve approach. Each version of the empirical Bayes method along with the bootstrap and FIQT lead to an average improvement in estimated MSE of between 65 and 70% with the implementation of the empirical Bayes algorithm which incorporates shape constrained additive models (SCAMs) having the greatest average improvement of just over 70%.

## Discussion

In this article, we investigated the problem of *Winner’s Curse* bias which results in the estimated effect sizes of significant SNPs often being greater than their true values. Our work concentrated on methods that could be used to reduce this bias in settings in which only summary statistics of the GWAS that discovered these SNP-trait associations were available. We chose to focus on this particular situation as *Winner’s Curse* correction methods which only require GWAS summary data tend to be very computational efficient and furthermore, this summary data is often much easier to access than individual-level data.

We performed a thorough evaluation and comparison of these methods using both simulated and real data sets. Our simulation study considered a wide range of genetic architectures including data sets in which a simple correlation structure had been imposed on the set of SNPs as well as data sets of independent SNPs. In addition, three UKBB real data sets were used for method evaluation purposes. As well as assessing currently published correction methods, we also explored several possible modifications that could be made in order to improve these methods. In particular, we looked at a number of variations of the empirical Bayes method and proposed an additional approach which uses the parametric bootstrap in order to establish suitable corrections for the estimated effect size of each SNP. The estimated mean squared error (MSE) was chosen as an appropriate metric in order to compare the methods. Due to the notable lack of software for implementation of *Winner’s Curse* correction methods, we developed an R package, ‘winnerscurse’, as an accompaniment to the work described in this paper. This allows users to apply all methods discussed here, as well as the proposed modifications, to their sets of GWAS summary data.

As a first step in both our simulation study and engagement with real data, we computed the proportion of significant SNPs that were significantly overestimated and observed the common trend that as the number of SNPs passing the significance threshold increased, the proportion of those that were significantly overestimated decreased. This aligns with the postulation that as sample sizes increase, *Winner’s Curse* bias becomes less of a concern although it still exists. However, caution must be taken when working with real data sets, especially those of binary traits, in which a very small number of SNPs have been deemed significant at a certain threshold. In this instance, it may be that the significant SNPs are representative of only one or two independent signals. For example, in our first T2D data set, while using a threshold of 5 × 10^−8^, we witnessed 93.5% of significant SNPs having greater replication effect size estimates than those obtained in the discovery GWAS. We note that our brief empirical investigation, as detailed in the supplementary material, suggested that at least 30 independent significant signals are required in order to ensure appropriate performance of the *Winner’s Curse* correction methods. Fortunately, as sample sizes increase in the future and different diseases have greater numbers of true signals captured by their respective sets of significant SNPs, this issue will only be seen to present itself in rare circumstances.

With respect to method performance, it was clear that the conditional likelihood methods performed poorly as in most instances, especially for *p* < 5 × 10^−8^, these methods resulted in greater values for the estimated MSE among significant SNPs than the naïve approach. The other considered methods behaved much more similarly to the extent that we cannot state that there is a clear advantage of one method over another. Thus, the choice of which method a user should apply to their set of GWAS summary statistics in order to correct for *Winner’s Curse* is dependent on personal preference. However, it is advised that when doing so, the possible limitations of the chosen method are understood well. Notably, the empirical Bayes methods have a clear theoretical advantage, that is, when the true effect size distribution is known, empirical Bayes is fully Bayesian and minimizes the average MSE of the standardized estimated effects as estimates of the true standardized effects over all competing estimators. Unfortunately, performance of empirical Bayes estimators can be restricted in practice due to inaccurate estimation of the extreme tails of the *z*-statistic distribution. This estimation difficulty is particularly problematic when the existence of strong linkage disequilibrium results in clusters of associations in the tails. These clusters can be falsely detected as local modes in the distribution by automatic fitting algorithms. Some progress on improving estimation in the tails has been made here with the proposal of modifications that employ generalized additive models or shape constrained additive models. However, these adaptations have not resulted in large enough improvements in order to claim objective superiority of the empirical Bayes methods over other approaches such as the bootstrap method or FIQT. In a setting in which the distribution of effect sizes is asymmetric, methods like the empirical Bayes and bootstrap, where the correction rule is not a function of absolute value *z*-statistics, possess the potential to perform better than FIQT and conditional likelihood methods. In spite of this fact, no tangible evidence of improved performance over FIQT was observed on the real data sets that we examined.

With both our set of simulations and real data analysis, we have aimed to be as comprehensive as possible as it is possible that differing method performance results may occur under differing genetic architectures, but this is an obviously difficult task. Informing these simulations appropriately is particularly challenging, especially when attempting to define the true effect size distribution. However, under the assumption of independent SNPs, we also investigated scenarios which had a bimodal or skewed distribution of effect sizes, as described in the supplementary material. Furthermore, for simulations involving correlated SNPs, we have assumed a very simplistic linkage disequilibrium (LD) structure in which the minor allele frequencies have been simulated independently of this LD structure. In contrast, the use of real data permitted the analysis of method performance in a realistic setting where a large degree of LD exists. However, this was limited to only three UKBB data sets. In the case of the binary trait T2D, it must be noted that due to the very small number of significant SNPs at *p* < 5 × 10^−8^, the results are deemed rather questionable here.

Due to space considerations, *Winner’s Curse* correction methods which require both a discovery and replication GWAS in order to make suitable adjustments to estimated effect sizes have not been examined in this manuscript, even though several of these methods have been implemented in our developed R package, ‘winnerscurse’. Furthermore, computation of standard errors of the adjusted estimated effect sizes have not been considered here. However, for methods such as the empirical Bayes, bootstrap and FIQT, the R package, ‘winnerscurse’, utilizes the parametric bootstrap in order to obtain these standard errors. This package can also be used to provide confidence intervals for estimated effect sizes which have been corrected for *Winner’s Curse* using the conditional likelihood methods. In two-sample Mendelian randomization, it is known that as this *Winner’s Curse* bias can be present in the estimated SNP-exposure associations, the causal estimate will then suffer from bias. Thus, the *Winner’s Curse* correction methods explored in this paper can also be potentially used as plug-in corrections for two-sample MR. In addition, these methods could prove beneficial in the computation of polygenic risk scores, in order to reduce the effect of *Winner’s Curse* bias there.

## Supporting information

S1 Fig Number of significant SNPs at threshold 5 × 10^−8^ plotted against the proportion of those SNPs with significantly overestimated effect sizes, for each simulation setting with a simple correlation structure imposed on the set of SNPs. The simulation settings are defined by combinations of three parameters, sample size *n*, heritability *h*^2^ and polygenicity *π*, as shown in the table. The legend underneath the plot indicates which colour corresponds to which scenario.
(TIFF)

S2 Fig *Z*-statistics plotted against bias for each simulation setting, with a simple correlation structure imposed on the set of SNPs. The *z*-statistic of a SNP is defined as its estimated effect size divided by the standard error of that estimated effect size while the bias of a SNP is equal to its true effect size subtracted from its estimated effect size. Plot subtitles show the sample size *n*, heritability *h*^2^ and polygenicity *π* values of each setting. Dark red dashed vertical lines represent the *z*-statistic corresponding to a *p*-value of 5 × 10^−8^ while light red dashed vertical lines represent the significance threshold of 5 × 10^−4^. Dark grey points highlight SNPs with significantly overestimated effect sizes that have *p*-values less than 5 × 10^−4^.
(TIFF)

S3 Fig Average bias of significant SNPs with *positive* association estimates at threshold 5 × 10^−8^ for each method and simulation setting, with a simple correlation structure imposed on the set of SNPs. (TIFF)

S4 Fig Average bias of significant SNPs with *negative* association estimates at threshold 5 × 10^−8^ for each method and simulation setting, with a simple correlation structure imposed on the set of SNPs. (TIFF)

S5 Fig Estimated change in RMSE of significant SNPs at threshold 5 × 10^−4^ for each method and simulation setting, with a simple correlation structure imposed on the set of SNPs. (TIFF)

S6 Fig Average bias of significant SNPs with *positive* association estimates at threshold 5 × 10^−4^ for each method and simulation setting, with a simple correlation structure imposed on the set of SNPs. (TIFF)

S7 Fig Average bias of significant SNPs with *negative* association estimates at threshold 5 × 10^−4^ for each method and simulation setting, with a simple correlation structure imposed on the set of SNPs. (TIFF)

S8 Fig Estimated change in RMSE of significant SNPs at threshold 5 × 10^−8^ for each method and simulation setting, assuming a quantitative trait, independent SNPs and a normal effect size distribution. (TIFF)

S9 Fig Estimated change in RMSE of significant SNPs at threshold 5 × 10^−4^ for each method and simulation setting, assuming a quantitative trait, independent SNPs and a normal effect size distribution. (TIFF)

S10 Fig Estimated change in RMSE of significant SNPs at threshold 5 × 10^−8^ for each method and simulation setting, assuming a quantitative trait, independent SNPs and a bimodal effect size distribution. (TIFF)

S11 Fig Estimated change in RMSE of significant SNPs at threshold 5 × 10^−4^ for each method and simulation setting, assuming a quantitative trait, independent SNPs and a bimodal effect size distribution. (TIFF)

S12 Fig Estimated change in RMSE of significant SNPs at threshold 5 × 10^−8^ for each method and simulation setting, assuming a quantitative trait, independent SNPs and a skewed effect size distribution. (TIFF)

S13 Fig Estimated change in RMSE of significant SNPs at threshold 5 × 10^−4^ for each method and simulation setting, assuming a quantitative trait, independent SNPs and a skewed effect size distribution. (TIFF)

S14 Fig Estimated change in RMSE of significant SNPs at threshold 5 × 10^−8^ for each method and simulation setting, assuming a binary trait, independent SNPs and a normal effect size distribution. (TIFF)

S15 Fig Estimated change in RMSE of significant SNPs at threshold 5 × 10^−4^ for each method and simulation setting, assuming a binary trait, independent SNPs and a normal effect size distribution. (TIFF)

S16 Fig Z-statistics plotted against bias for each real data set. The *z*-statistic of a SNP is defined as its estimated effect size divided by the standard error of that estimated effect size while the estimated bias of a SNP is defined by Eq [16] in the main text. Dark red dashed vertical lines represent the *z*-statistic corresponding to a *p*-value of 5 × 10^−8^ while light red dashed vertical lines represent the significance threshold of 5 × 10^−4^. Dark grey points highlight SNPs with significantly overestimated effect sizes that have *p*-values less than 5 × 10^−4^.
(TIFF)

S17 Fig Average bias of significant SNPs with *positive* association estimates at threshold 5 × 10^−8^ for each method and data set. The darker green bar and dashed black line represent the average bias of the naïve approach.
(TIFF)

S18 Fig Average bias of significant SNPs with *negative* association estimates at threshold 5 × 10^−8^ for each method and data set. The darker green bar and dashed black line represent the average bias of the naïve approach. Note the absence of bars for all methods for the T2D data sets here as both data sets had zero SNPs with negative association estimates that were deemed significant at this threshold.
(TIFF)

S19 Fig Average bias of significant SNPs with *positive* association estimates at threshold 5 × 10^−4^ for each method and data set. The darker green bar and dashed black line represent the average bias of the naïve approach.
(TIFF)

S20 Fig Average bias of significant SNPs with *negative* association estimates at threshold 5 × 10^−4^ for each method and data set. The darker green bar and dashed black line represent the average bias of the naïve approach.
(TIFF)

S21 Fig Estimated MSE of significant SNPs at threshold 5 × 10^−8^ for each method and *pruned* data set. The darker green bar and dashed black line represent the average bias of the naïve approach.
(TIFF)

S22 Fig Estimated MSE of significant SNPs at threshold 5 × 10^−4^ for each method and *pruned* data set. The darker green bar and dashed black line represent the average bias of the naïve approach.
(TIFF)

S1 Table The average number and MSE of significant SNPs at two significance thresholds, 5 × 10^−8^ and 5 × 10^−4^, with proportions that indicate the extent of *Winner’s Curse* bias for each simulation scenario, with a simple correlation structure imposed on the set of SNPs. The parameters defining each simulation scenario are shown at the top. As well as the number of significant SNPs and their naïve MSE, S1 Table also contains the proportion of significant SNPs that were seen to have a larger estimated effect size than their true effect size, in terms of absolute value, as well as the proportion of significant SNPs that have significantly overestimated effect sizes. Values provided are averages obtained across 100 simulated sets of summary statistiscs.
(DOCX)

S2 Table Estimated change in RMSE of significant SNPs at threshold 5 × 10^−8^ for each method and simulation setting, with a simple correlation structure imposed on the set of SNPs. The parameters defining each simulation scenario are shown at the top. Values provided are averages obtained across 100 simulated sets of summary statistiscs. Positive values are shaded in grey, indicating poor performing methods, while light green shaded cells highlight the method which, on average, resulted in the greatest reduction in RMSE for each scenario.
(DOCX)

S3 Table Estimated relative change in RMSE of significant SNPs at threshold 5 × 10^−8^ for each method and simulation setting, with a simple correlation structure imposed on the set of SNPs. The parameters defining each simulation scenario are shown at the top. Values provided are averages obtained across 100 simulated sets of summary statistiscs. Positive values are shaded in grey, indicating poor performing methods, while light green shaded cells highlight the method which, on average, resulted in the greatest relative reduction in RMSE for each scenario. As the final column contains the mean of each row, it shows that the bootstrap method has the greatest average estimated relative reduction in RMSE. This value of -0.1497 suggests that on average, the bootstrap method improves the RMSE of significant SNPs by ≈14.97%.
(DOCX)

S4 Table Average bias of significant SNPs with *positive* association estimates at threshold 5 × 10^−8^ for each method and simulation setting, with a simple correlation structure imposed on the set of SNPs. The parameters defining each simulation scenario are shown at the top. Values provided are averages obtained across 100 simulated sets of summary statistiscs. Values that are greater, in absolute value, than their corresponding naïve value are shaded in grey, while light green shaded cells highlight the method which, on average, resulted in the smallest absolute bias for each scenario.
(DOCX)

S5 Table Average bias of significant SNPs with *negative* association estimates at threshold 5 × 10^−8^ for each method and simulation setting, with a simple correlation structure imposed on the set of SNPs. The parameters defining each simulation scenario are shown at the top. Values provided are averages obtained across 100 simulated sets of summary statistiscs. Values that are greater, in absolute value, than their corresponding naïve value are shaded in grey, while light green shaded cells highlight the method which, on average, resulted in the smallest absolute bias for each scenario.
(DOCX)

S6 Table Estimated change in RMSE of significant SNPs at threshold 5 × 10^−4^ for each method and simulation setting, with a simple correlation structure imposed on the set of SNPs. The parameters defining each simulation scenario are shown at the top. Values provided are averages obtained across 100 simulated sets of summary statistiscs. Positive values are shaded in grey, indicating poor performing methods, while light green shaded cells highlight the method which, on average, resulted in the greatest reduction in RMSE for each scenario.
(DOCX)

S7 Table Estimated relative change in RMSE of significant SNPs at threshold 5 × 10^−4^ for each method and simulation setting, with a simple correlation structure imposed on the set of SNPs. The parameters defining each simulation scenario are shown at the top. Values provided are averages obtained across 100 simulated sets of summary statistiscs. Positive values are shaded in grey, indicating poor performing methods, while light green shaded cells highlight the method which, on average, resulted in the greatest relative reduction in RMSE for each scenario. As the final column contains the mean of each row, it shows that the original empirical Bayes method has the greatest average estimated relative reduction in RMSE. This value of -0.1935 suggests that on average, this form of the empirical Bayes method improves the RMSE of significant SNPs by ≈19.35%.
(DOCX)

S8 Table Average bias of significant SNPs with *positive* association estimates at threshold 5 × 10^−4^ for each method and simulation setting, with a simple correlation structure imposed on the set of SNPs. The parameters defining each simulation scenario are shown at the top. Values provided are averages obtained across 100 simulated sets of summary statistiscs. Values that are greater, in absolute value, than their corresponding naïve value are shaded in grey, while light green shaded cells highlight the method which, on average, resulted in the smallest absolute bias for each scenario.
(DOCX)

S9 Table Average bias of significant SNPs with *negative* association estimates at threshold 5 × 10^−4^ for each method and simulation setting, with a simple correlation structure imposed on the set of SNPs. The parameters defining each simulation scenario are shown at the top. Values provided are averages obtained across 100 simulated sets of summary statistiscs. Values that are greater, in absolute value, than their corresponding naïve value are shaded in grey, while light green shaded cells highlight the method which, on average, resulted in the smallest absolute bias for each scenario.
(DOCX)

S10 Table Average bias of significant SNPs with *positive* association estimates at threshold 5 × 10^−8^ for each method and data set. The first row provides the average bias obtained if the unadjusted estimated effect sizes of the discovery GWAS are used. Values that are greater than their corresponding naïve value are shaded in grey while light green shaded cells highlight the method that resulted in the smallest absolute bias for each data set.
(DOCX)

S11 Table Average bias of significant SNPs with *negative* association estimates at threshold 5 × 10^−8^ for each method and data set. The first row provides the average bias obtained if the unadjusted estimated effect sizes of the discovery GWAS are used. Values that are greater than their corresponding naïve value are shaded in grey while light green shaded cells highlight the method that resulted in the smallest absolute bias for each data set. Note that the T2D data sets are absent from this table as both data sets had zero SNPs with negative association estimates that were deemed significant at this threshold.
(DOCX)

S12 Table Estimated MSE of significant SNPs at threshold 5 × 10^−4^ for each method and data set. The first row provides the estimated MSE obtained if the unadjusted estimated effect sizes of the discovery GWAS are used. Values that are greater than their corresponding naïve value are shaded in grey while light green shaded cells highlight the method that resulted in the lowest estimated MSE value for each data set.
(DOCX)

S13 Table Average bias of significant SNPs with *positive* association estimates at threshold 5 × 10^−4^ for each method and data set. The first row provides the average bias obtained if the unadjusted estimated effect sizes of the discovery GWAS are used. Values that are greater than their corresponding naïve value are shaded in grey while light green shaded cells highlight the method that resulted in the smallest absolute bias for each data set.
(DOCX)

S14 Table Average bias of significant SNPs with *negative* association estimates at threshold 5 × 10^−4^ for each method and data set. The first row provides the average bias obtained if the unadjusted estimated effect sizes of the discovery GWAS are used. Values that are greater than their corresponding naïve value have been shaded in grey while light green shaded cells highlight the method that resulted in the smallest absolute bias for each data set.
(DOCX)

S15 Table Estimated MSE of significant SNPs at threshold 5 × 10^−8^ for each method and *pruned* data set. The first row provides the estimated MSE obtained if the unadjusted estimated effect sizes of the discovery GWAS are used. Values that are greater than their corresponding naïve value are shaded in grey while light green shaded cells highlight the method that resulted in the lowest estimated MSE value for each data set.
(DOCX)

S16 Table Estimated MSE of significant SNPs at threshold 5 × 10^−4^ for each method and *pruned* data set. The first row provides the estimated MSE obtained if the unadjusted estimated effect sizes of the discovery GWAS are used. Values that are greater than their corresponding naïve value are shaded in grey while light green shaded cells highlight the method that resulted in the lowest estimated MSE value for each data set.
(DOCX)

S1 File Text Supplement. This file contains a more detailed description of the various proposed modifications to the empirical Bayes method, the simulation process and the evaluation of method performance using simulated data sets of independent SNPs. It also includes a derivation of the estimated MSE of significant SNPs with respect to real data sets, as well as discussion of the use of pruned real data sets and the number of independent signals required in order to ensure the use of a *Winner’s Curse* correction method is suitable.
(PDF)
