# Supplementary figures and images for: Review and further developments in statistical corrections for Winner’s Curse in genetic association studies
Source: PLoS Genet. 2023 Sep 18;19(9):e1010546. doi: 10.1371/journal.pgen.1010546 (PMC10538662; doi:10.1371/journal.pgen.1010546)

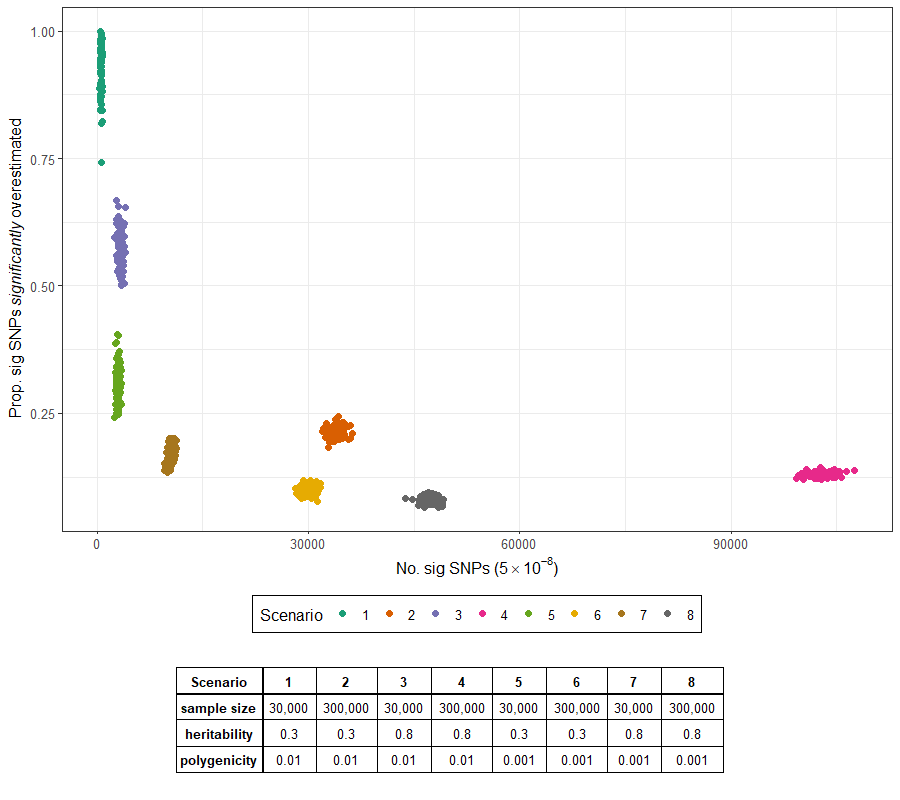

Supplement: S1 Fig — The simulation settings are defined by combinations of three parameters, sample size n, heritability h2 and polygenicity π, as shown in the table. The legend underneath the plot indicates which colour corresponds to which scenario. (TIFF) [file pgen.1010546.s001.tiff]

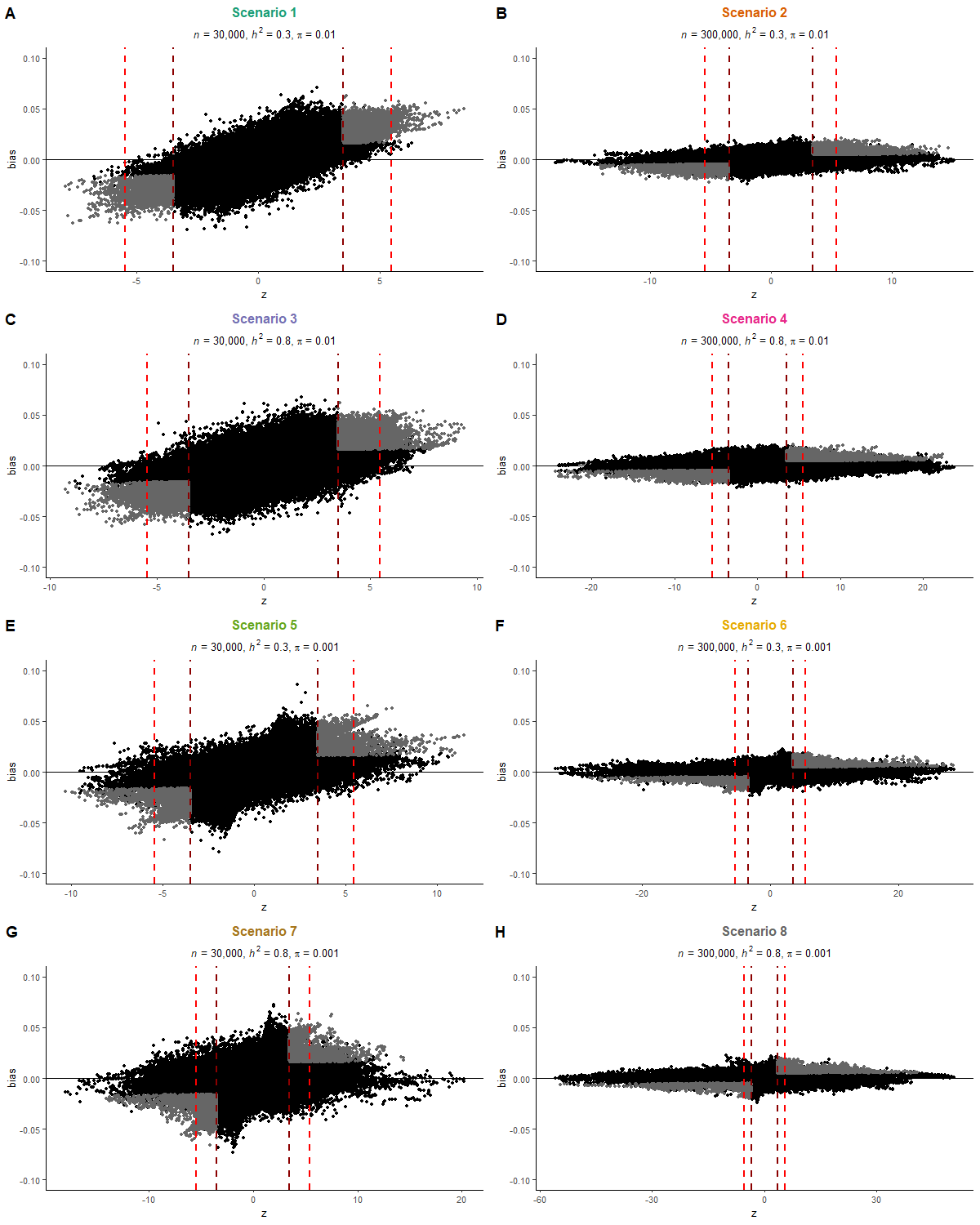

Supplement: S2 Fig — The z-statistic of a SNP is defined as its estimated effect size divided by the standard error of that estimated effect size while the bias of a SNP is equal to its true effect size subtracted from its estimated effect size. Plot subtitles show the sample size n, heritability h2 and polygenicity π values of each setting. Dark red dashed vertical lines represent the z-statistic corresponding to a p-value of 5 × 10^−8^ while light red dashed vertical lines represent the significance threshold of 5 × 10^−4^. Dark grey points highlight SNPs with significantly overestimated effect sizes that have p-values less than 5 × 10^−4^. (TIFF) [file pgen.1010546.s002.tiff]

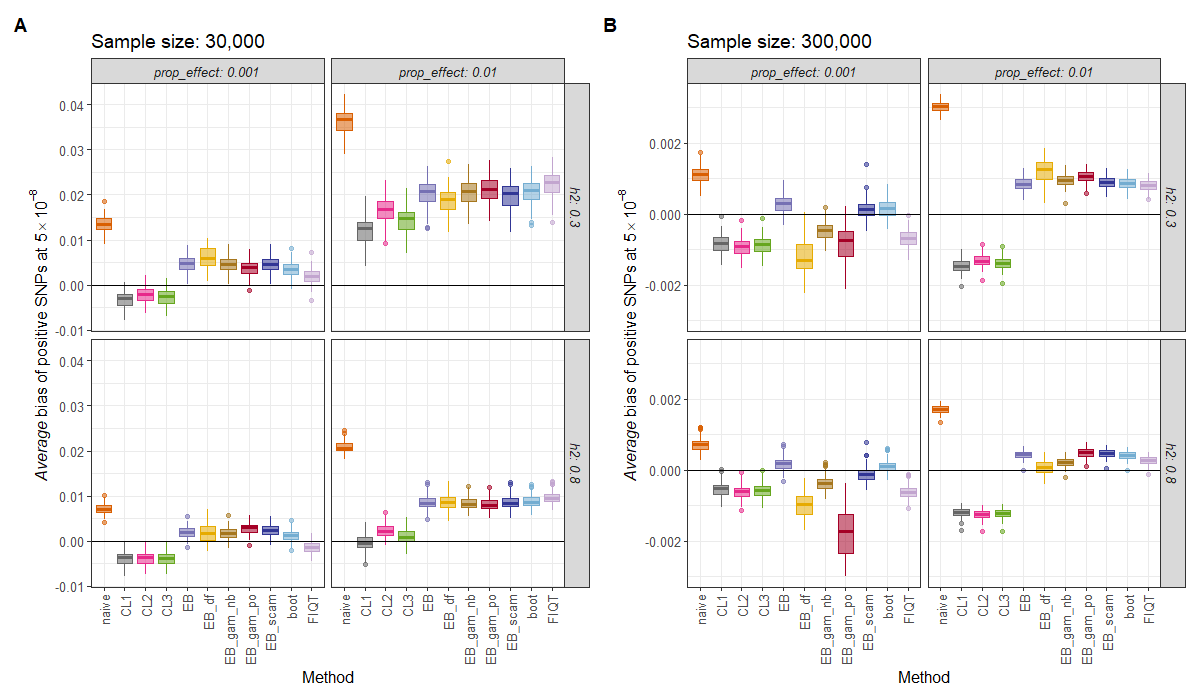

Supplement: S3 Fig — (TIFF) [file pgen.1010546.s003.tiff]

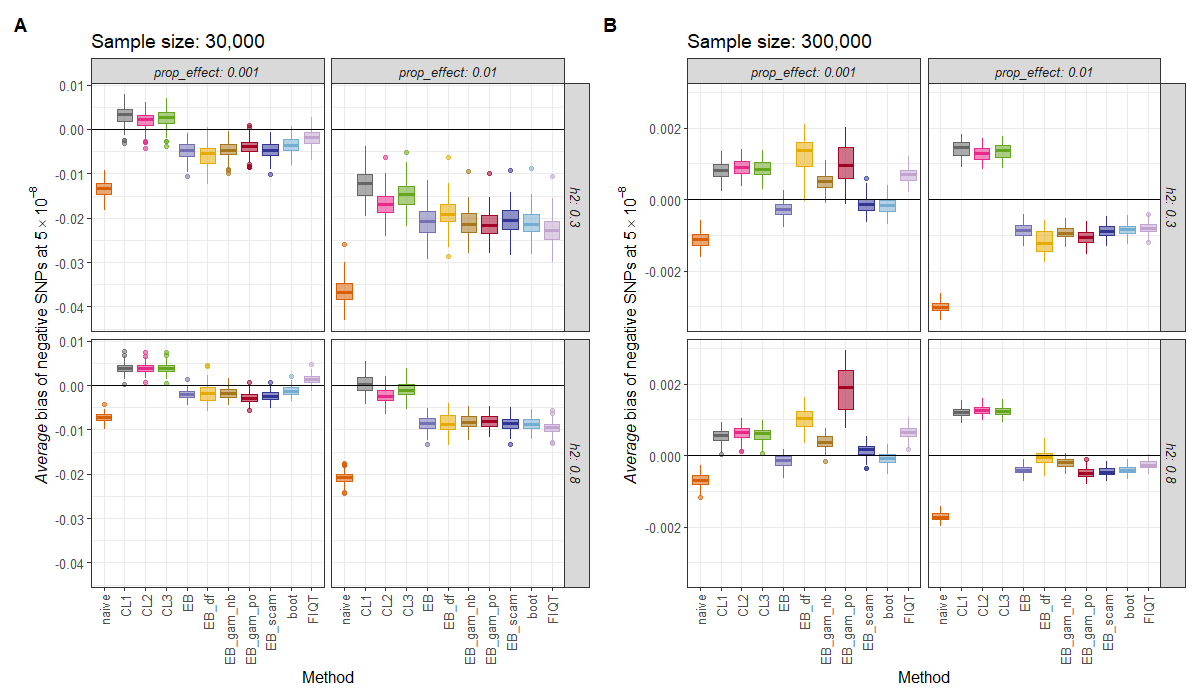

Supplement: S4 Fig — (TIFF) [file pgen.1010546.s004.tiff]

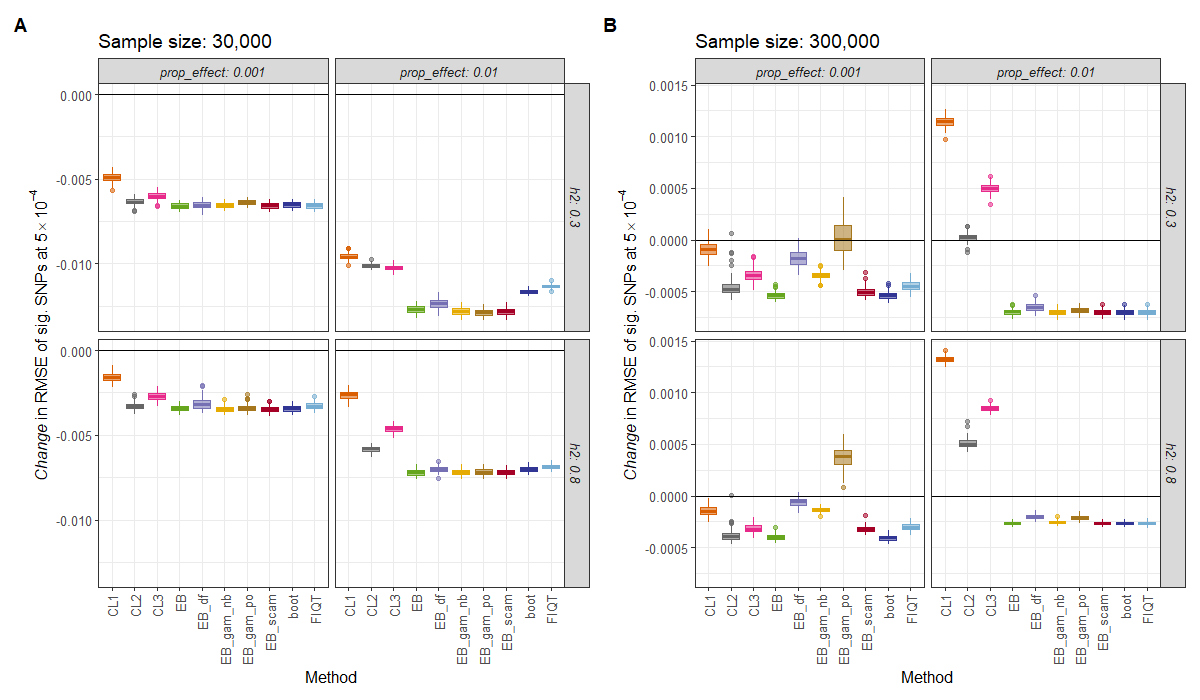

Supplement: S5 Fig — (TIFF) [file pgen.1010546.s005.tiff]

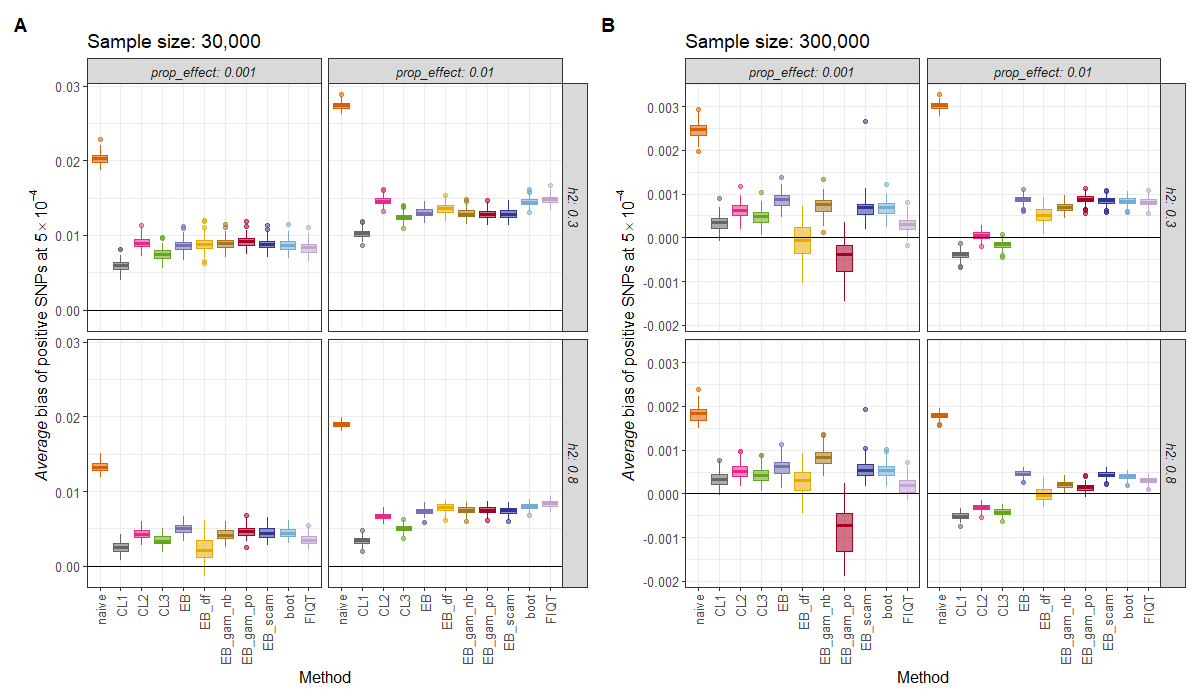

Supplement: S6 Fig — (TIFF) [file pgen.1010546.s006.tiff]

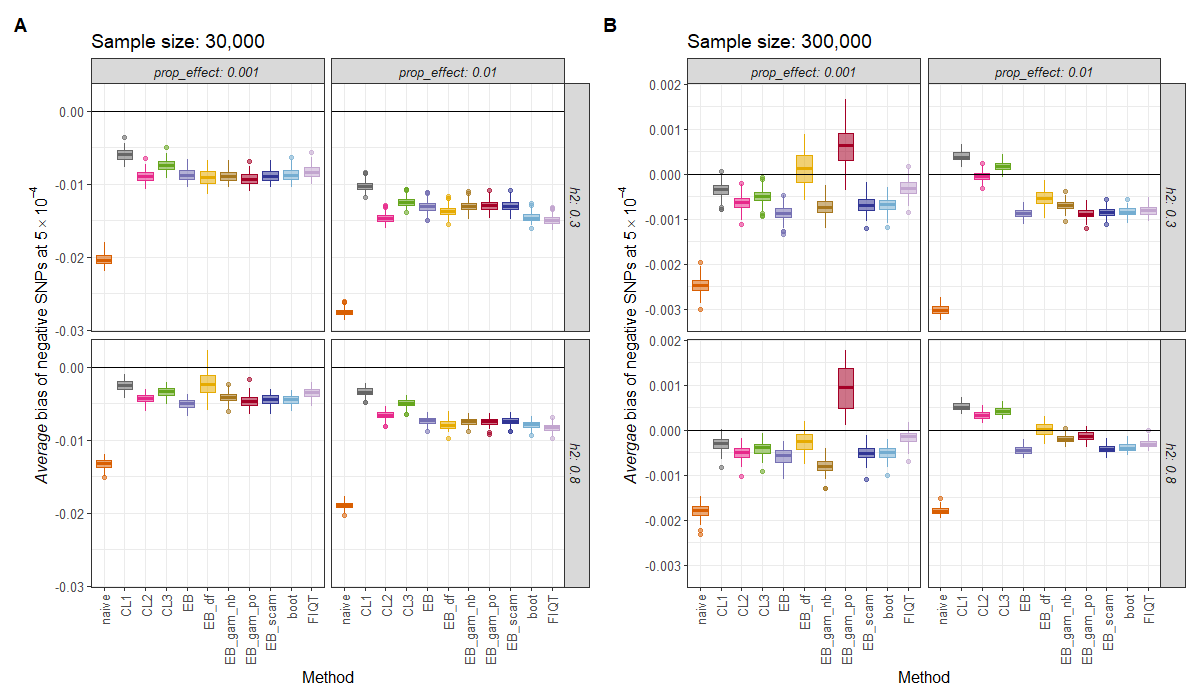

Supplement: S7 Fig — (TIFF) [file pgen.1010546.s007.tiff]

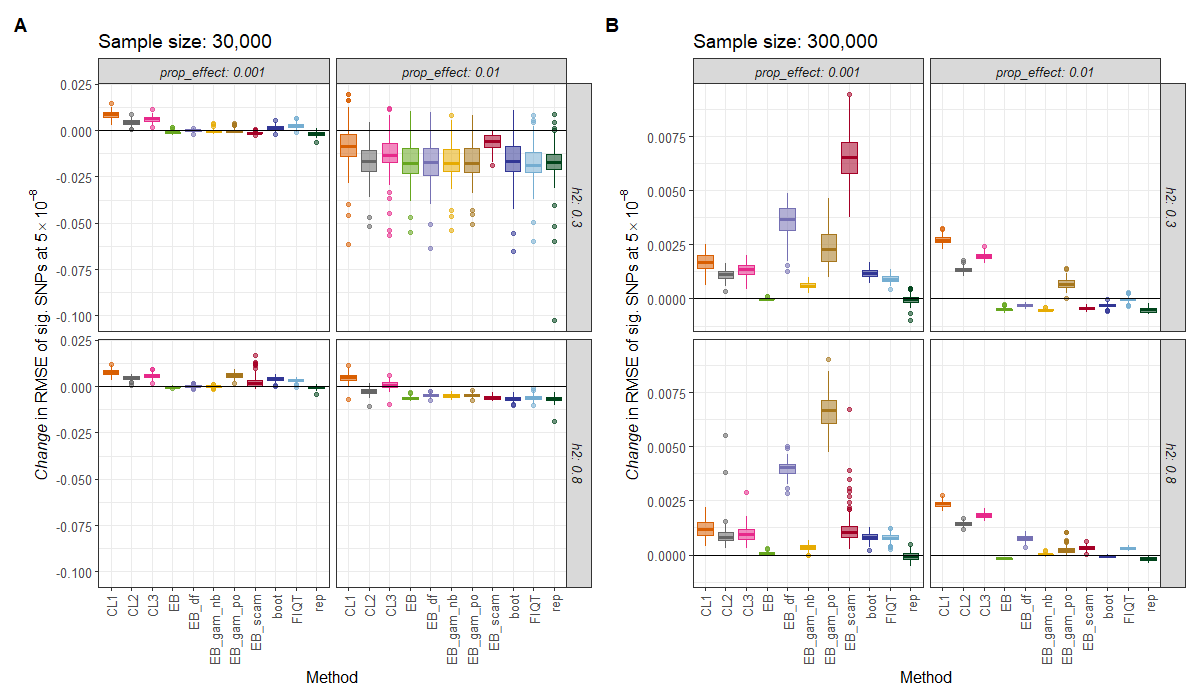

Supplement: S8 Fig — (TIFF) [file pgen.1010546.s008.tiff]

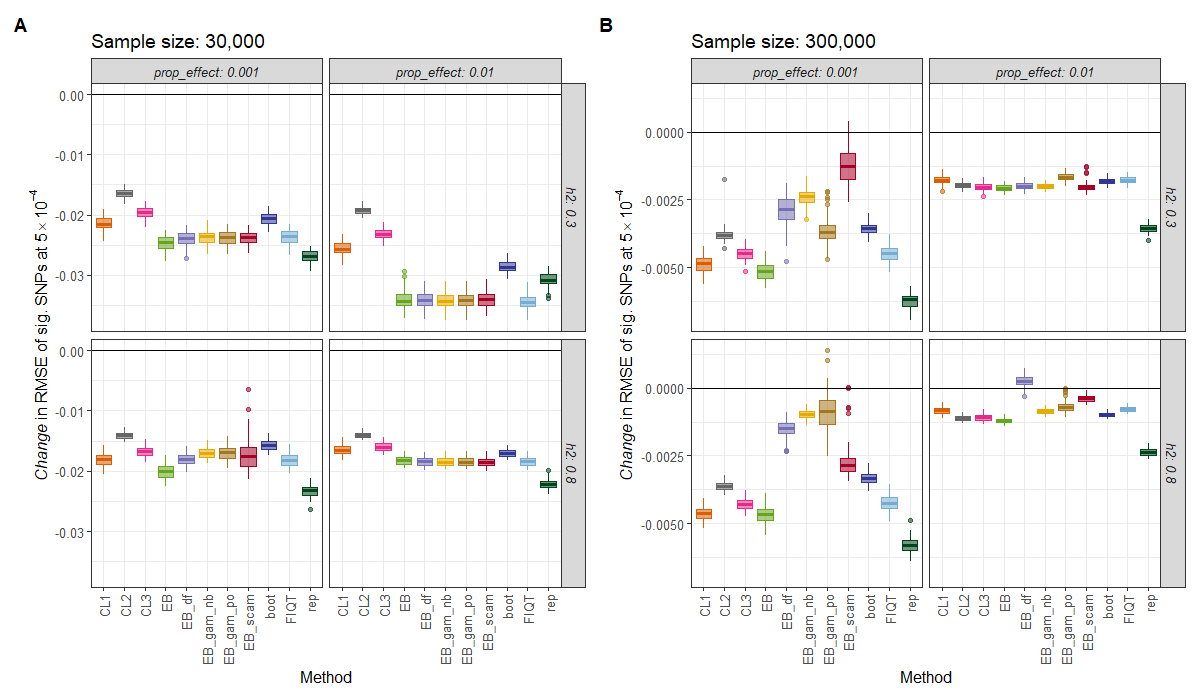

Supplement: S9 Fig — (TIFF) [file pgen.1010546.s009.tiff]

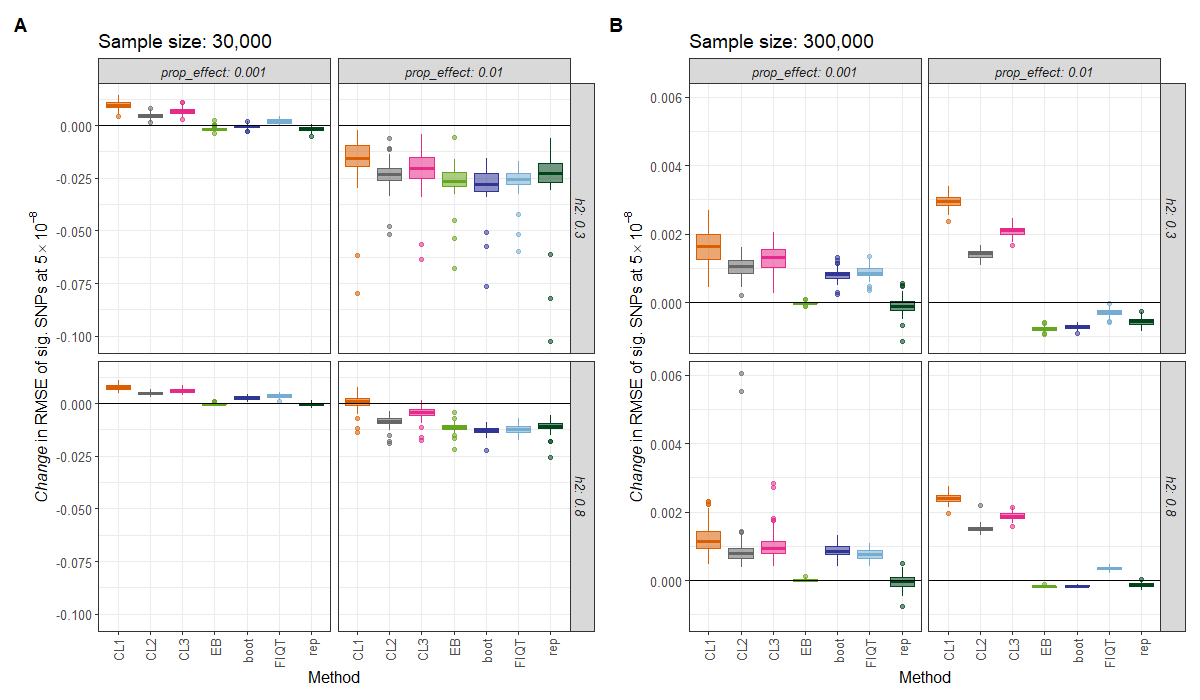

Supplement: S10 Fig — (TIFF) [file pgen.1010546.s010.tiff]

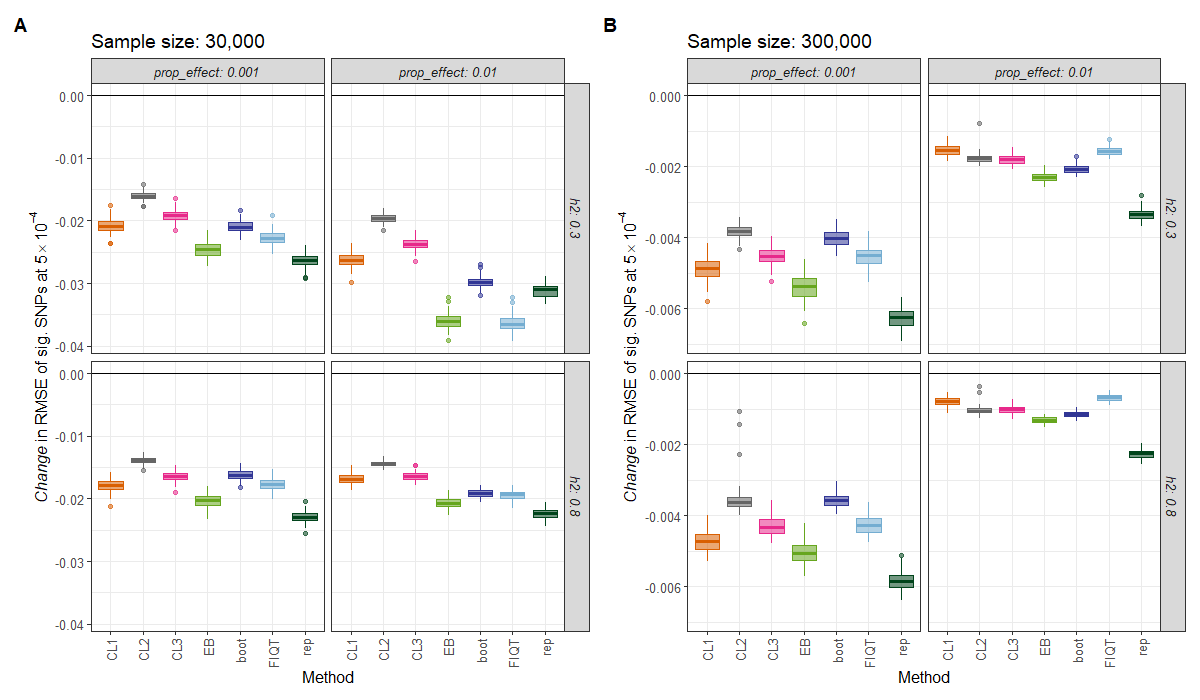

Supplement: S11 Fig — (TIFF) [file pgen.1010546.s011.tiff]

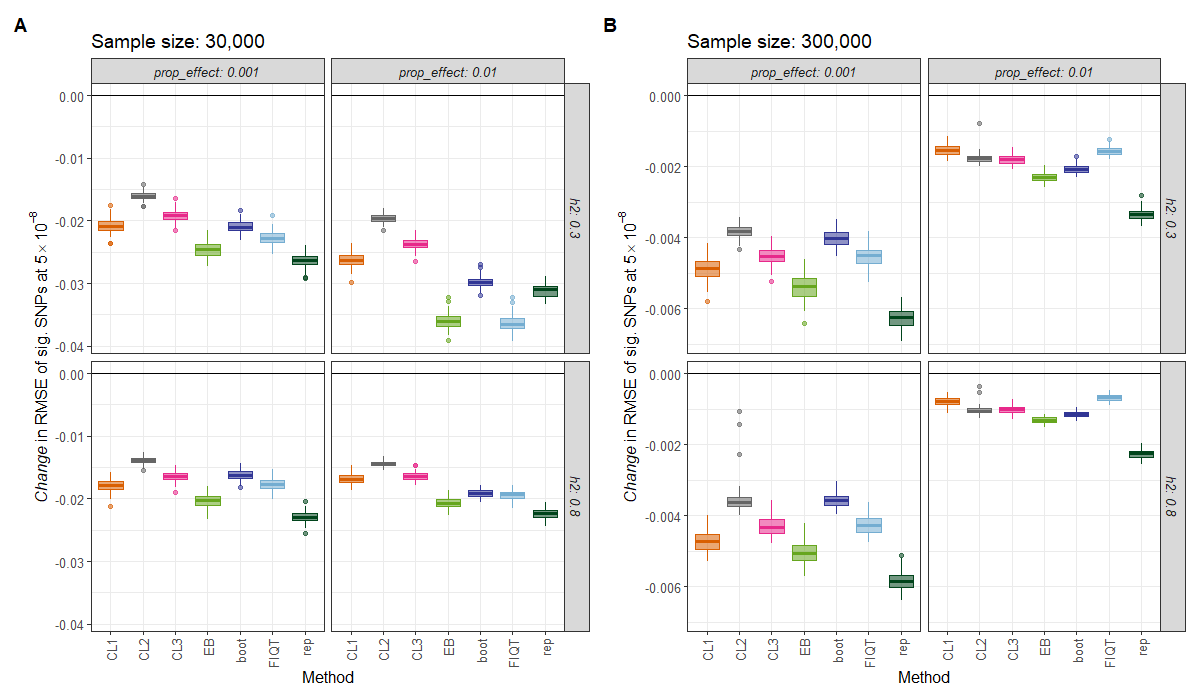

Supplement: S12 Fig — (TIFF) [file pgen.1010546.s012.tiff]

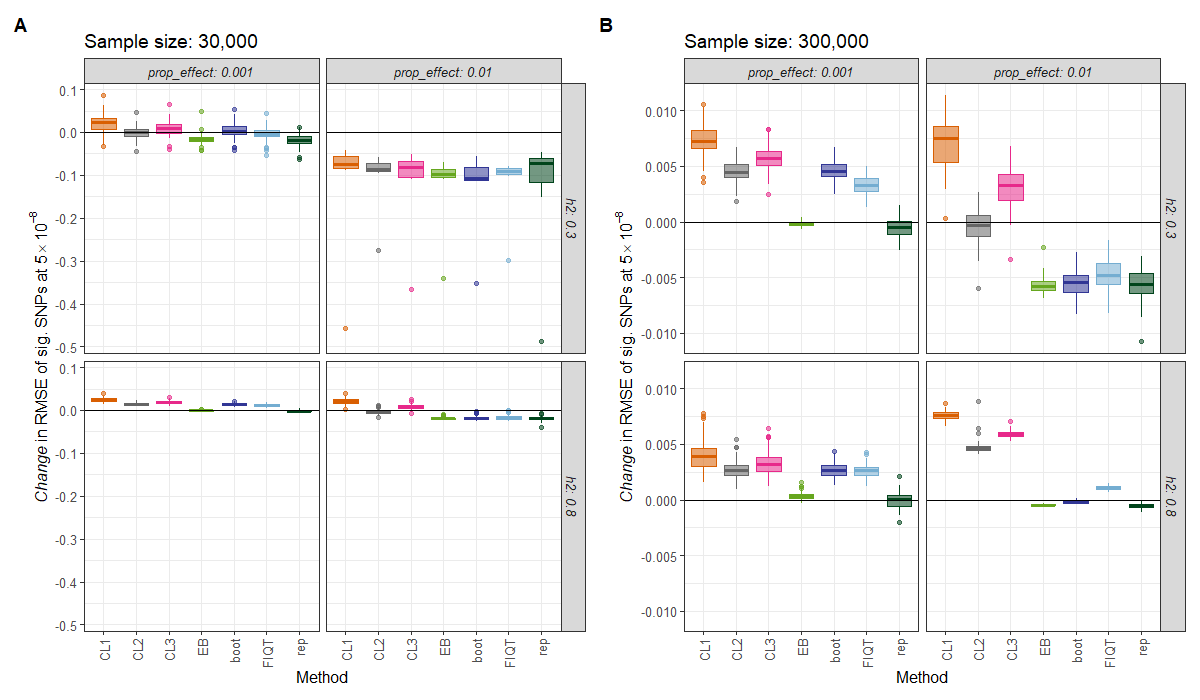

Supplement: S14 Fig — (TIFF) [file pgen.1010546.s014.tiff]

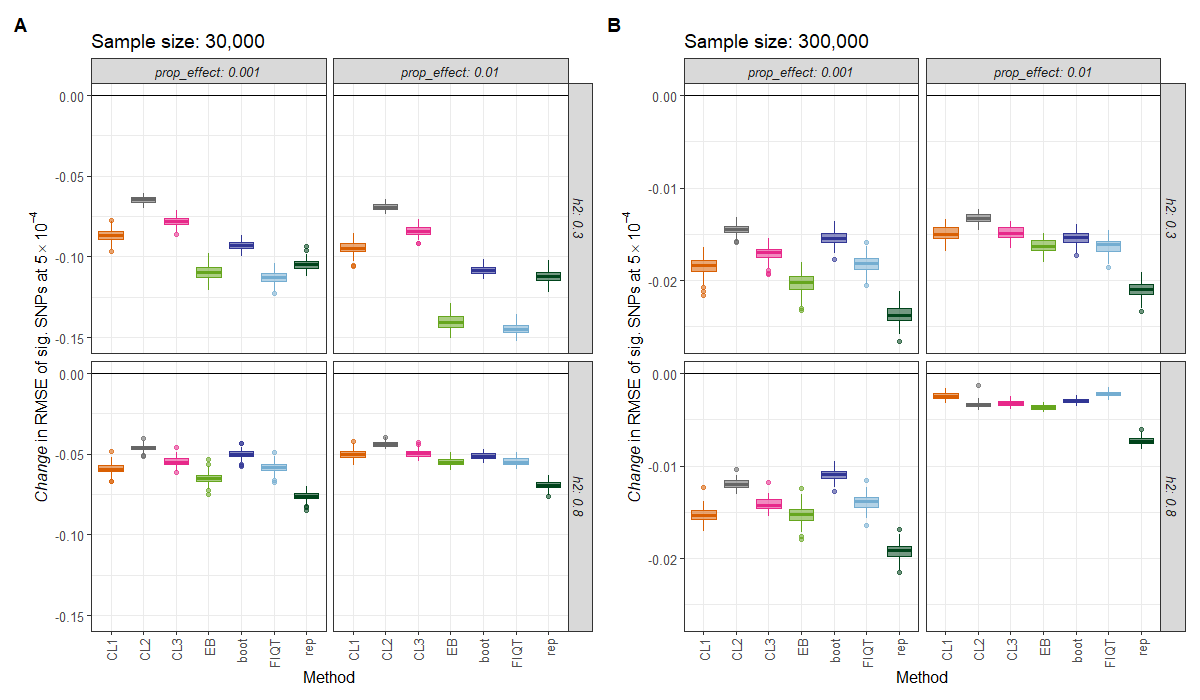

Supplement: S15 Fig — (TIFF) [file pgen.1010546.s015.tiff]

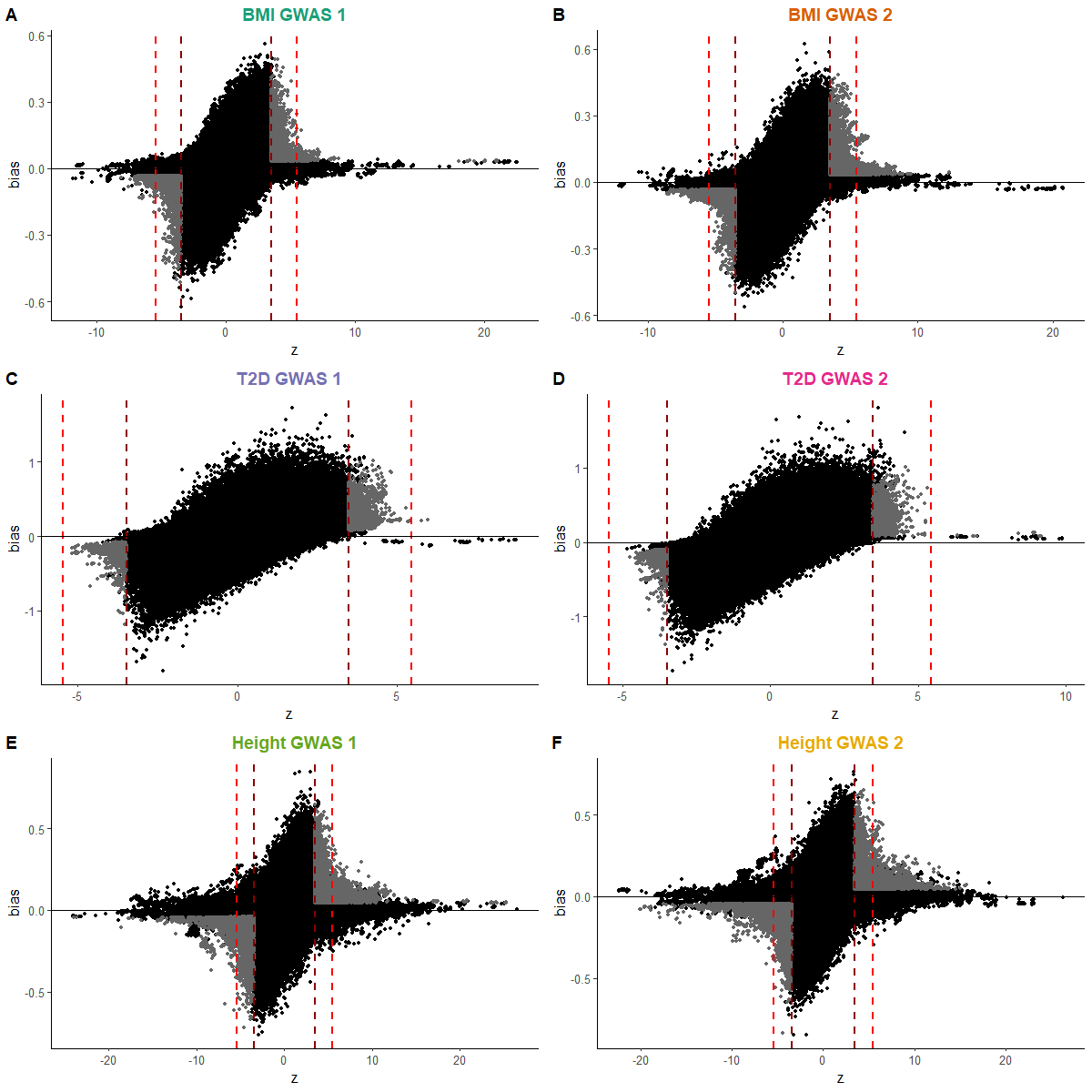

Supplement: S16 Fig — The z-statistic of a SNP is defined as its estimated effect size divided by the standard error of that estimated effect size while the estimated bias of a SNP is defined by Eq [16] in the main text. Dark red dashed vertical lines represent the z-statistic corresponding to a p-value of 5 × 10^−8^ while light red dashed vertical lines represent the significance threshold of 5 × 10^−4^. Dark grey points highlight SNPs with significantly overestimated effect sizes that have p-values less than 5 × 10^−4^. (TIFF) [file pgen.1010546.s016.tiff]

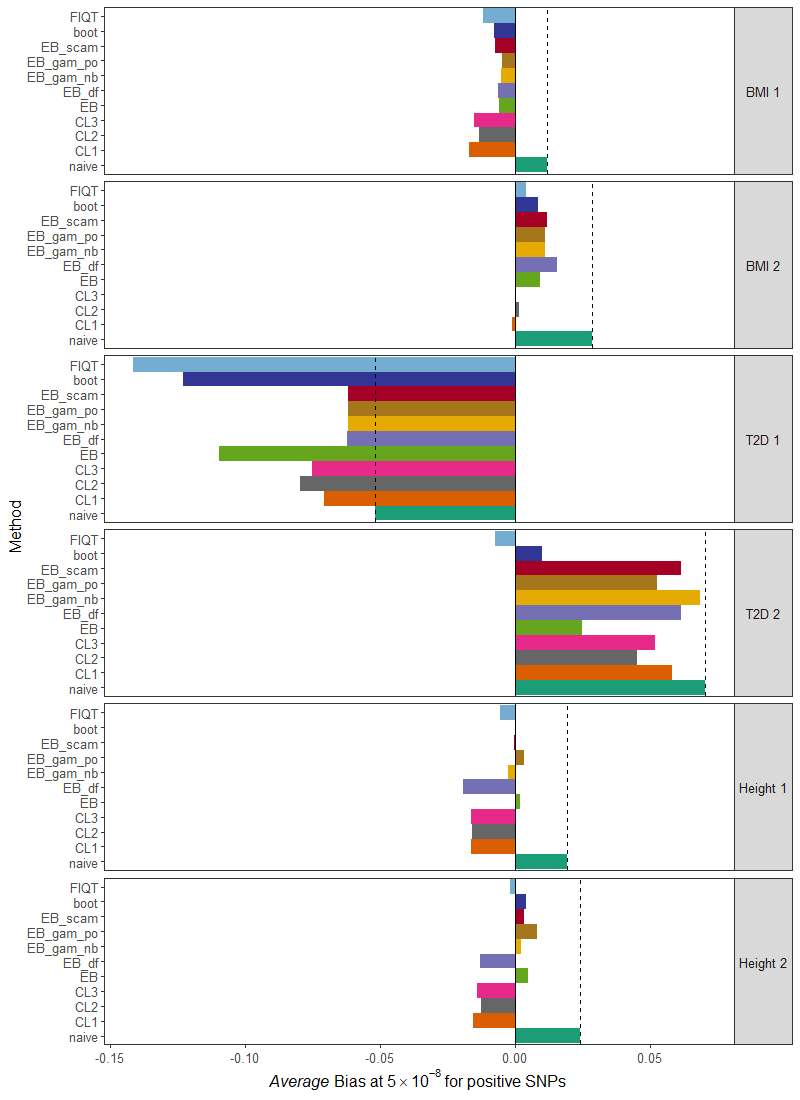

Supplement: S17 Fig — The darker green bar and dashed black line represent the average bias of the naïve approach. (TIFF) [file pgen.1010546.s017.tiff]

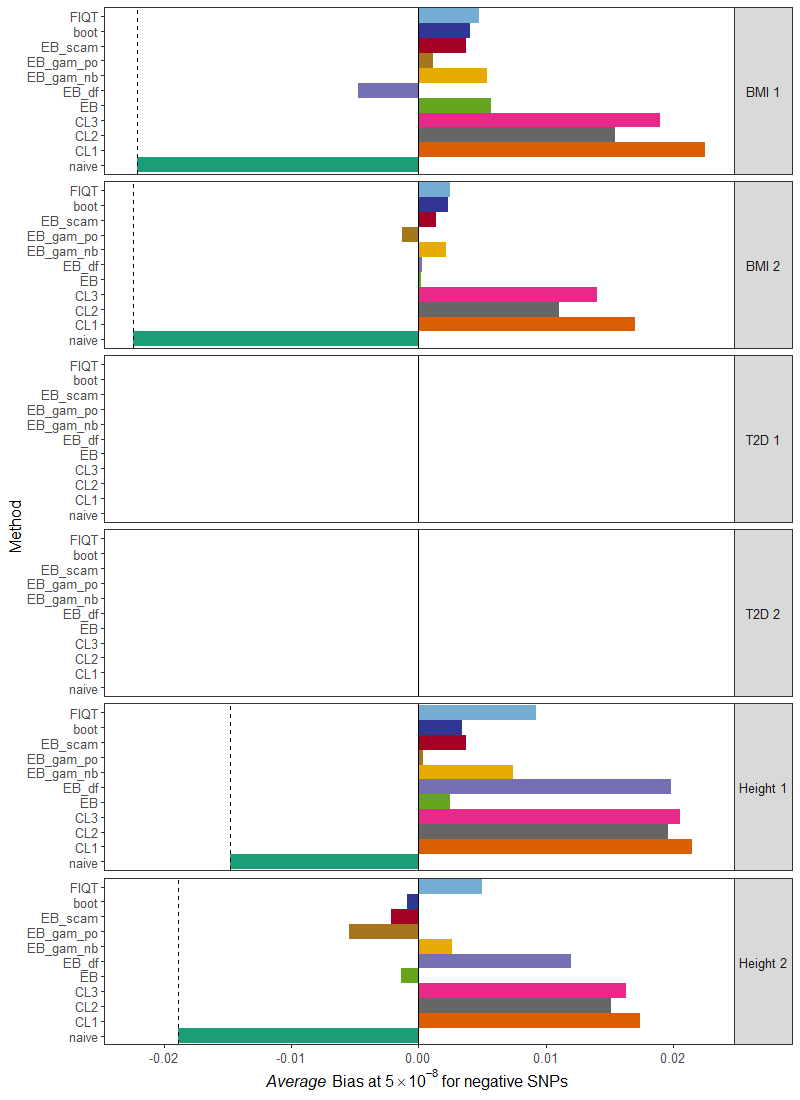

Supplement: S18 Fig — The darker green bar and dashed black line represent the average bias of the naïve approach. Note the absence of bars for all methods for the T2D data sets here as both data sets had zero SNPs with negative association estimates that were deemed significant at this threshold. (TIFF) [file pgen.1010546.s018.tiff]

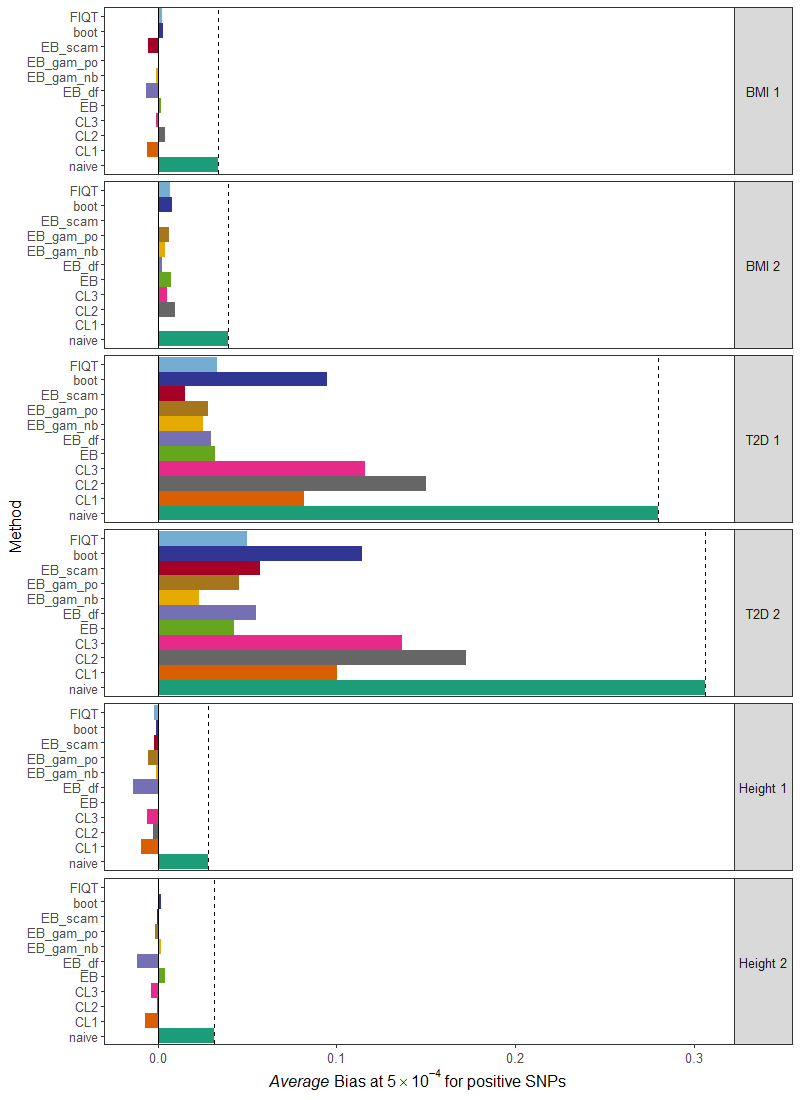

Supplement: S19 Fig — The darker green bar and dashed black line represent the average bias of the naïve approach. (TIFF) [file pgen.1010546.s019.tiff]

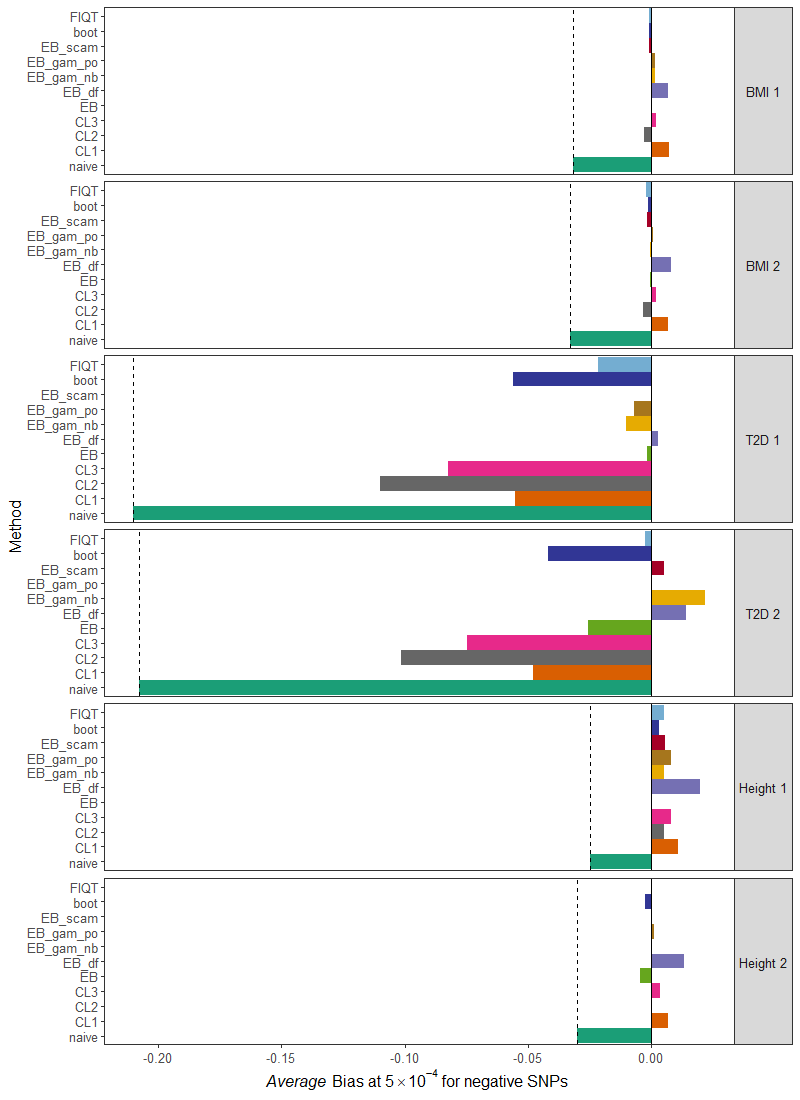

Supplement: S20 Fig — The darker green bar and dashed black line represent the average bias of the naïve approach. (TIFF) [file pgen.1010546.s020.tiff]

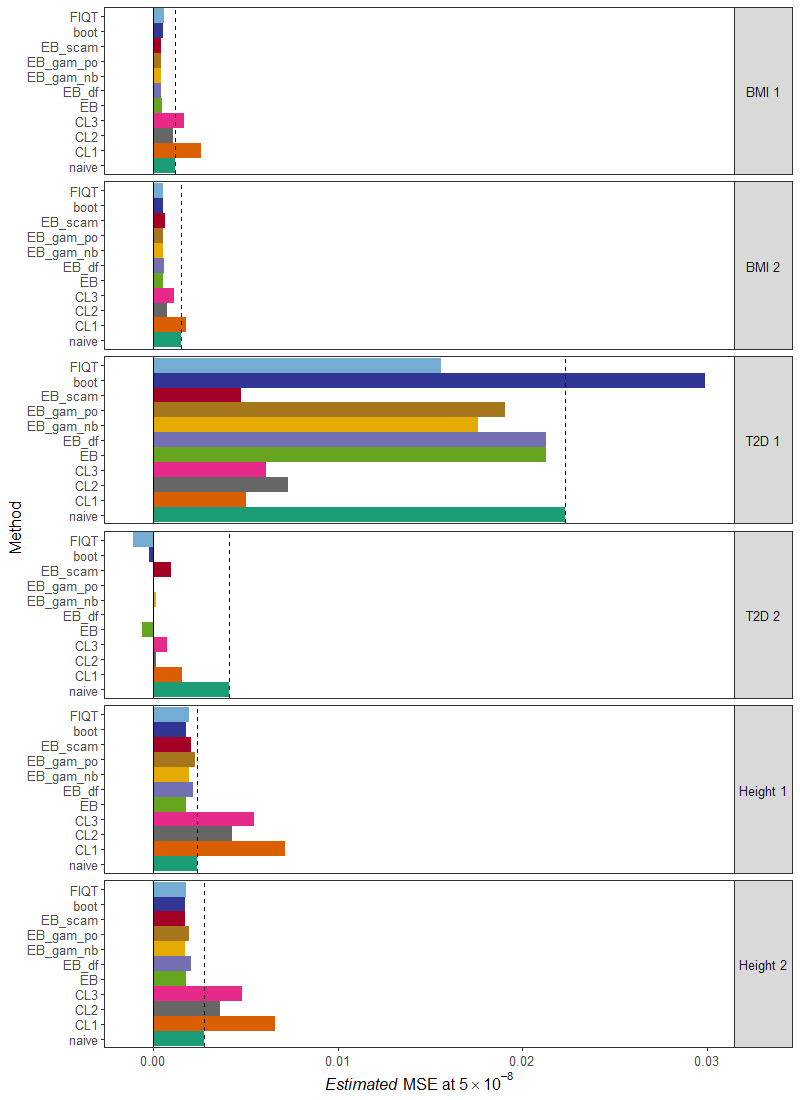

Supplement: S21 Fig — The darker green bar and dashed black line represent the average bias of the naïve approach. (TIFF) [file pgen.1010546.s021.tiff]

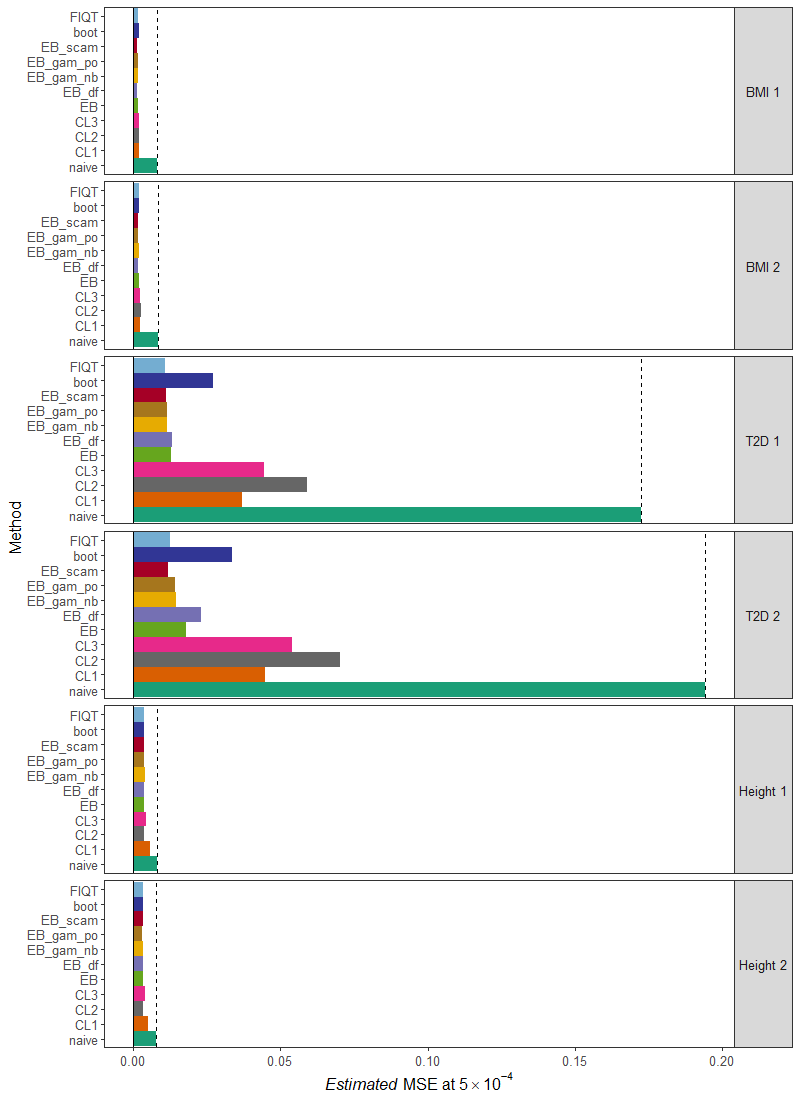

Supplement: S22 Fig — The darker green bar and dashed black line represent the average bias of the naïve approach. (TIFF) [file pgen.1010546.s022.tiff]
