# Supplementary material for: Review and further developments in statistical corrections for Winner’s Curse in genetic association studies
Source: PLoS Genet. 2023 Sep 18;19(9):e1010546. doi: 10.1371/journal.pgen.1010546 (PMC10538662; doi:10.1371/journal.pgen.1010546)
Supplement: S1 Table — The parameters defining each simulation scenario are shown at the top. As well as the number of significant SNPs and their naïve MSE, S1 Table also contains the proportion of significant SNPs that were seen to have a larger estimated effect size than their true effect size, in terms of absolute value, as well as the proportion of significant SNPs that have significantly overestimated effect sizes. Values provided are averages obtained across 100 simulated sets of summary statistiscs. (DOCX) [file pgen.1010546.s023.docx]

**S1 Table. The average number and MSE of significant SNPs at two significance thresholds, 5 × 10^-8^ and 5 × 10^-4^, with proportions that indicate the extent of *Winner’s Curse* bias for each simulation scenario, with a simple correlation structure imposed on the set of SNPs.**

| **Simulation scenario** | **1** | **2** | **3** | **4** | **5** | **6** | **7** | **8** |
| --- | --- | --- | --- | --- | --- | --- | --- | --- |
| **sample size *n*** | 30,000 | 300,000 | 30,000 | 300,000 | 30,000 | 300,000 | 30,000 | 300,000 |
| **heritability *h*^2^** | 0.3 | 0.3 | 0.8 | 0.8 | 0.3 | 0.3 | 0.8 | 0.8 |
| **polygenicity *π*** | 0.01 | 0.01 | 0.01 | 0.01 | 0.001 | 0.001 | 0.001 | 0.001 |
| **Metric** |  |  |  |  |  |  |  |  |
| **No. sig. SNPs**  **(5 × 10^-8^)** | 555 | 33,835 | 3,268 | 103,021 | 2,953 | 29,983 | 10,445 | 47,188 |
| **MSE of sig. SNPs**  **(5 × 10^-8^)** | 0.00146 | 0.000025 | 0.000621 | 0.000019 | 0.000398 | 0.000018 | 0.000231 | 0.000017 |
| **Prop. sig. SNPs with *larger* estimate**  **(5 × 10^-8^)** | 0.999 | 0.7771 | 0.9523 | 0.6684 | 0.8339 | 0.6088 | 0.7091 | 0.5651 |
| **Prop. sig. SNPs *significantly* overestimated**  **(5 × 10^-8^)** | 0.9326 | 0.2138 | 0.5715 | 0.13 | 0.3064 | 0.0998 | 0.1655 | 0.0805 |
| **No. sig. SNPs**  **(5 × 10^-4^)** | 16,709 | 114,020 | 36,120 | 212,293 | 19,751 | 54,428 | 32,654 | 69,354 |
| **MSE of sig. SNPs**  **(5 × 10^-4^)** | 0.000843 | 0.000026 | 0.00051 | 0.00002 | 0.00065 | 0.000032 | 0.000445 | 0.000028 |
| **Prop. sig. SNPs with *larger* estimate**  **(5 × 10^-4^)** | 0.9952 | 0.7699 | 0.9378 | 0.6698 | 0.8873 | 0.6625 | 0.774 | 0.6123 |
| **Prop. sig. SNPs *significantly* overestimated**  **(5 × 10^-4^)** | 0.8403 | 0.2216 | 0.5169 | 0.1392 | 0.5512 | 0.2253 | 0.3559 | 0.1814 |

The parameters defining each simulation scenario are shown at the top. As well as the number of significant SNPs and their naïve MSE, Table S1 also contains the proportion of significant SNPs that were seen to have a larger estimated effect size than their true effect size, in terms of absolute value, as well as the proportion of significant SNPs that have significantly overestimated effect sizes. Values provided are averages obtained across 100 simulated sets of summary statistiscs.
