# Supplementary material for: Review and further developments in statistical corrections for Winner’s Curse in genetic association studies
Source: PLoS Genet. 2023 Sep 18;19(9):e1010546. doi: 10.1371/journal.pgen.1010546 (PMC10538662; doi:10.1371/journal.pgen.1010546)
Supplement: S6 Table — The parameters defining each simulation scenario are shown at the top. Values provided are averages obtained across 100 simulated sets of summary statistiscs. Positive values are shaded in grey, indicating poor performing methods, while light green shaded cells highlight the method which, on average, resulted in the greatest reduction in RMSE for each scenario. (DOCX) [file pgen.1010546.s028.docx]

**S6 Table. Estimated change in RMSE of significant SNPs at threshold 5 × 10^-4^** **for each method and simulation setting, with a simple correlation structure imposed on the set of SNPs.**

| **Simulation scenario** | **1** | **2** | **3** | **4** | **5** | **6** | **7** | **8** |
| --- | --- | --- | --- | --- | --- | --- | --- | --- |
| **sample size *n*** | 30,000 | 300,000 | 30,000 | 300,000 | 30,000 | 300,000 | 30,000 | 300,000 |
| **heritability *h*^2^** | 0.3 | 0.3 | 0.8 | 0.8 | 0.3 | 0.3 | 0.8 | 0.8 |
| **polygenicity *π*** | 0.01 | 0.01 | 0.01 | 0.01 | 0.01 | 0.001 | 0.001 | 0.001 |
| **Method** |  |  |  |  |  |  |  |  |
| **CL1** | -0.009565 | 0.001145 | -0.002652 | 0.001324 | -0.004908 | -0.00009 | -0.001573 | -0.000147 |
| **CL2** | -0.010104 | 0.000023 | -0.005838 | 0.000508 | -0.006345 | -0.000457 | -0.003298 | -0.000384 |
| **CL3** | -0.010252 | 0.000501 | -0.004626 | 0.000846 | -0.005981 | -0.000343 | -0.002724 | -0.000317 |
| **EB** | -0.012711 | -0.000697 | -0.007196 | -0.000266 | -0.006605 | -0.000538 | -0.003446 | -0.000398 |
| **EB df=7** | -0.01233 | -0.000651 | -0.007007 | -0.0002 | -0.006539 | -0.000178 | -0.003159 | -0.000058 |
| **EB scam** | -0.012816 | -0.0007 | -0.007199 | -0.000265 | -0.006562 | -0.000502 | -0.003496 | -0.000322 |
| **EB gam-po** | -0.012895 | -0.000683 | -0.007168 | -0.000212 | -0.006375 | 0.00002 | -0.003403 | 0.000366 |
| **EB gam-nb** | -0.012813 | -0.000701 | -0.007174 | -0.000254 | -0.006551 | -0.000347 | -0.003471 | -0.000134 |
| **boot** | -0.01163 | -0.000702 | -0.007018 | -0.000267 | -0.006536 | -0.000537 | -0.003446 | -0.000406 |
| **FIQT** | -0.011317 | -0.000703 | -0.006862 | -0.000265 | -0.006565 | -0.000444 | -0.003294 | -0.0003 |

The parameters defining each simulation scenario are shown at the top. Values provided are averages obtained across 100 simulated sets of summary statistiscs. Positive values are shaded in grey, indicating poor performing methods, while light green shaded cells highlight the method which, on average, resulted in the greatest reduction in RMSE for each scenario.
