# Supplementary material for: Review and further developments in statistical corrections for Winner’s Curse in genetic association studies
Source: PLoS Genet. 2023 Sep 18;19(9):e1010546. doi: 10.1371/journal.pgen.1010546 (PMC10538662; doi:10.1371/journal.pgen.1010546)
Supplement: S7 Table — The parameters defining each simulation scenario are shown at the top. Values provided are averages obtained across 100 simulated sets of summary statistiscs. Positive values are shaded in grey, indicating poor performing methods, while light green shaded cells highlight the method which, on average, resulted in the greatest relative reduction in RMSE for each scenario. As the final column contains the mean of each row, it shows that the original empirical Bayes method has the greatest average estimated relative reduction in RMSE. This value of -0.1935 suggests that on average, this form of the empirical Bayes method improves the RMSE of significant SNPs by ≈19.35%. (DOCX) [file pgen.1010546.s029.docx]

**S7 Table. Estimated relative change in RMSE of significant SNPs at threshold 5 × 10^-4^** **for each method and simulation setting, with a simple correlation structure imposed on the set of SNPs.**

| **Simulation scenario** | **1** | **2** | **3** | **4** | **5** | **6** | **7** | **8** |  |
| --- | --- | --- | --- | --- | --- | --- | --- | --- | --- |
| **sample size *n*** | 30,000 | 300,000 | 30,000 | 300,000 | 30,000 | 300,000 | 30,000 | 300,000 |  |
| **heritability *h*^2^** | 0.3 | 0.3 | 0.8 | 0.8 | 0.3 | 0.3 | 0.8 | 0.8 |  |
| **polygenicity *π*** | 0.01 | 0.01 | 0.01 | 0.01 | 0.01 | 0.001 | 0.001 | 0.001 |  |
| **Method** |  |  |  |  |  |  |  |  |  |
| **CL1** | -0.3297 | 0.2252 | -0.1177 | 0.2974 | -0.1926 | -0.0159 | -0.0746 | -0.0276 | -0.0294 |
| **CL2** | -0.3483 | 0.0046 | -0.2592 | 0.1142 | -0.2490 | -0.0811 | -0.1565 | -0.0723 | -0.1310 |
| **CL3** | -0.3534 | 0.0986 | -0.2054 | 0.1901 | -0.2347 | -0.0609 | -0.1293 | -0.0598 | -0.0944 |
| **EB** | -0.4382 | -0.1370 | -0.3195 | -0.0596 | -0.2592 | -0.0957 | -0.1636 | -0.0750 | -0.1935 |
| **EB df=7** | -0.4250 | -0.1279 | -0.3111 | -0.0448 | -0.2566 | -0.0315 | -0.1499 | -0.0110 | -0.1697 |
| **EB scam** | -0.4418 | -0.1377 | -0.3196 | -0.0596 | -0.2575 | -0.0893 | -0.1660 | -0.0607 | -0.1915 |
| **EB gam-po** | -0.4445 | -0.1343 | -0.3183 | -0.0475 | -0.2502 | 0.0038 | -0.1615 | 0.0691 | -0.1604 |
| **EB gam-nb** | -0.4417 | -0.1379 | -0.3185 | -0.0571 | -0.2571 | -0.0616 | -0.1648 | -0.0253 | -0.1830 |
| **boot** | -0.4009 | -0.1380 | -0.3116 | -0.0599 | -0.2565 | -0.0955 | -0.1636 | -0.0766 | -0.1878 |
| **FIQT** | -0.3901 | -0.1383 | -0.3047 | -0.0595 | -0.2577 | -0.0789 | -0.1564 | -0.0566 | -0.1803 |

The parameters defining each simulation scenario are shown at the top. Values provided are averages obtained across 100 simulated sets of summary statistiscs. Positive values are shaded in grey, indicating poor performing methods, while light green shaded cells highlight the method which, on average, resulted in the greatest relative reduction in RMSE for each scenario. As the final column contains the mean of each row, it shows that the original empirical Bayes method has the greatest average estimated relative reduction in RMSE. This value of -0.1935 suggests that on average, this form of the empirical Bayes method improves the RMSE of significant SNPs by ≈19.35%.
