# Supplementary material for: Review and further developments in statistical corrections for Winner’s Curse in genetic association studies
Source: PLoS Genet. 2023 Sep 18;19(9):e1010546. doi: 10.1371/journal.pgen.1010546 (PMC10538662; doi:10.1371/journal.pgen.1010546)
Supplement: S8 Table — The parameters defining each simulation scenario are shown at the top. Values provided are averages obtained across 100 simulated sets of summary statistiscs. Values that are greater, in absolute value, than their corresponding naïve value are shaded in grey, while light green shaded cells highlight the method which, on average, resulted in the smallest absolute bias for each scenario. (DOCX) [file pgen.1010546.s030.docx]

**S8 Table. Average bias of significant SNPs with *positive* association estimates at threshold 5 × 10^-4^** **for each method and simulation setting, with a simple correlation structure imposed on the set of SNPs.**

| **Simulation scenario** | **1** | **2** | **3** | **4** | **5** | **6** | **7** | **8** |
| --- | --- | --- | --- | --- | --- | --- | --- | --- |
| **sample size *n*** | 30,000 | 300,000 | 30,000 | 300,000 | 30,000 | 300,000 | 30,000 | 300,000 |
| **heritability *h*^2^** | 0.3 | 0.3 | 0.8 | 0.8 | 0.3 | 0.3 | 0.8 | 0.8 |
| **polygenicity *π*** | 0.01 | 0.01 | 0.01 | 0.01 | 0.001 | 0.001 | 0.001 | 0.001 |
| **Method** |  |  |  |  |  |  |  |  |
| **naive** | 0.027447 | 0.003011 | 0.018973 | 0.001792 | 0.020343 | 0.002453 | 0.013256 | 0.001821 |
| **CL1** | 0.010230 | -0.000397 | 0.003354 | -0.000511 | 0.005936 | 0.000340 | 0.002532 | 0.000328 |
| **CL2** | 0.014591 | 0.000048 | 0.006645 | -0.000318 | 0.008965 | 0.000624 | 0.004336 | 0.000519 |
| **CL3** | 0.012411 | -0.000175 | 0.005000 | -0.000415 | 0.007450 | 0.000482 | 0.003434 | 0.000423 |
| **EB** | 0.013008 | 0.000869 | 0.007328 | 0.000456 | 0.008691 | 0.000860 | 0.005060 | 0.000607 |
| **EB df=7** | 0.013573 | 0.000526 | 0.007835 | -0.000011 | 0.008827 | -0.000066 | 0.002277 | 0.000299 |
| **EB scam** | 0.012880 | 0.000841 | 0.007393 | 0.000434 | 0.008857 | 0.000682 | 0.004453 | 0.000554 |
| **EB gam-po** | 0.012790 | 0.000875 | 0.007487 | 0.000142 | 0.009223 | -0.000483 | 0.004668 | -0.000851 |
| **EB-gam-nb** | 0.012914 | 0.000690 | 0.007484 | 0.000208 | 0.008908 | 0.000735 | 0.004196 | 0.000829 |
| **boot** | 0.014452 | 0.000836 | 0.007932 | 0.000400 | 0.008701 | 0.000671 | 0.004457 | 0.000527 |
| **FIQT** | 0.014796 | 0.000811 | 0.008334 | 0.000302 | 0.008326 | 0.000291 | 0.003554 | 0.000178 |

The parameters defining each simulation scenario are shown at the top. Values provided are averages obtained across 100 simulated sets of summary statistiscs. Values that are greater, in absolute value, than their corresponding naïve value are shaded in grey, while light green shaded cells highlight the method which, on average, resulted in the smallest absolute bias for each scenario.
