# Supplementary material for: Review and further developments in statistical corrections for Winner’s Curse in genetic association studies
Source: PLoS Genet. 2023 Sep 18;19(9):e1010546. doi: 10.1371/journal.pgen.1010546 (PMC10538662; doi:10.1371/journal.pgen.1010546)
Supplement: S11 Table — The first row provides the average bias obtained if the unadjusted estimated effect sizes of the discovery GWAS are used. Values that are greater than their corresponding naïve value are shaded in grey while light green shaded cells highlight the method that resulted in the smallest absolute bias for each data set. Note that the T2D data sets are absent from this table as both data sets had zero SNPs with negative association estimates that were deemed significant at this threshold. (DOCX) [file pgen.1010546.s033.docx]

**S11 Table. Average bias of significant SNPs with *negative* association estimates at threshold 5 × 10^-8^** **for each method and data set.**

| **GWAS** | **BMI 1** | **BMI 2** | **Height 1** | **Height 2** |
| --- | --- | --- | --- | --- |
| **naive** | -0.02212 | -0.02242 | -0.01478 | -0.01887 |
| **CL1** | 0.02255 | 0.01699 | 0.02149 | 0.01743 |
| **CL2** | 0.01545 | 0.01105 | 0.01958 | 0.01510 |
| **CL3** | 0.01900 | 0.01402 | 0.02054 | 0.01627 |
| **EB** | 0.00571 | 0.00021 | 0.00245 | -0.00138 |
| **EB df=7** | -0.00476 | 0.00028 | 0.01984 | 0.01202 |
| **EB scam** | 0.00374 | 0.00138 | 0.00377 | -0.00213 |
| **EB gam-po** | 0.00113 | -0.00126 | 0.00032 | -0.00546 |
| **EB gam-nb** | 0.00541 | 0.00214 | 0.00740 | 0.00265 |
| **boot** | 0.00406 | 0.00235 | 0.00341 | -0.00093 |
| **FIQT** | 0.00474 | 0.00249 | 0.00926 | 0.00498 |

The first row provides the average bias obtained if the unadjusted estimated effect sizes of the discovery GWAS are used. Values that are greater than their corresponding naïve value are shaded in grey while light green shaded cells highlight the method that resulted in the smallest absolute bias for each data set. Note that the T2D data sets are absent from this table as both data sets had zero SNPs with negative association estimates that were deemed significant at this threshold.
