# Supplementary material for: Review and further developments in statistical corrections for Winner’s Curse in genetic association studies
Source: PLoS Genet. 2023 Sep 18;19(9):e1010546. doi: 10.1371/journal.pgen.1010546 (PMC10538662; doi:10.1371/journal.pgen.1010546)
Supplement: S15 Table — The first row provides the estimated MSE obtained if the unadjusted estimated effect sizes of the discovery GWAS are used. Values that are greater than their corresponding naïve value are shaded in grey while light green shaded cells highlight the method that resulted in the lowest estimated MSE value for each data set. (DOCX) [file pgen.1010546.s037.docx]

**S15 Table. Estimated MSE of significant SNPs at threshold 5 × 10^-8^** **for each method and *pruned* data set.**

| **GWAS** | **BMI 1** | **BMI 2** | **T2D 1** | **T2D 2** | **Height 1** | **Height 2** |
| --- | --- | --- | --- | --- | --- | --- |
| **naive** | 0.00120 | 0.00151 | 0.02233 | 0.00411 | 0.00239 | 0.00276 |
| **CL1** | 0.00260 | 0.00181 | 0.00502 | 0.00155 | 0.00716 | 0.00658 |
| **CL2** | 0.00110 | 0.00077 | 0.00732 | 0.00018 | 0.00427 | 0.00364 |
| **CL3** | 0.00169 | 0.00116 | 0.00612 | 0.00075 | 0.00545 | 0.00482 |
| **EB** | 0.00051 | 0.00056 | 0.02128 | -0.0006 | 0.00178 | 0.00178 |
| **EB df=7** | 0.00045 | 0.00059 | 0.02131 | -0.00001 | 0.00216 | 0.00204 |
| **EB** **scam** | 0.00043 | 0.00065 | 0.04764 | 0.00099 | 0.00205 | 0.00175 |
| **EB gam-po** | 0.00044 | 0.00055 | 0.01905 | 0.00007 | 0.00225 | 0.00197 |
| **EB gam-nb** | 0.00043 | 0.00054 | 0.01757 | 0.00015 | 0.00194 | 0.00174 |
| **boot** | 0.00053 | 0.00053 | 0.02993 | -0.00022 | 0.00180 | 0.00171 |
| **FIQT** | 0.00058 | 0.00054 | 0.01562 | -0.00107 | 0.00196 | 0.00179 |

The first row provides the estimated MSE obtained if the unadjusted estimated effect sizes of the discovery GWAS are used. Values that are greater than their corresponding naïve value are shaded in grey while light green shaded cells highlight the method that resulted in the lowest estimated MSE value for each data set.
