# Supplementary material for: Review and further developments in statistical corrections for Winner’s Curse in genetic association studies
Source: PLoS Genet. 2023 Sep 18;19(9):e1010546. doi: 10.1371/journal.pgen.1010546 (PMC10538662; doi:10.1371/journal.pgen.1010546)
Supplement: S1 File — This file contains a more detailed description of the various proposed modifications to the empirical Bayes method, the simulation process and the evaluation of method performance using simulated data sets of independent SNPs. It also includes a derivation of the estimated MSE of significant SNPs with respect to real data sets, as well as discussion of the use of pruned real data sets and the number of independent signals required in order to ensure the use of a Winner’s Curse correction method is suitable. (PDF) [file pgen.1010546.s039.pdf]

Supplement for: Review and further developments in statistical corrections for Winner's Curse in genetic association studies

Amanda Forde[1*], Gibran Hemani[2,3], John Ferguson[4]

[1]School of Mathematical and Statistical Sciences, University of Galway, Galway, Ireland

[2] MRC Integrative Epidemiology Unit, University of Bristol, Oakfield House, Oakfield Grove, Bristol, BS8 2BN, UK

[3] Population Health Sciences, Bristol Medical School, University of Bristol, Bristol, UK

[4]HRB Clinical Research Facility, NUI Galway, Galway, Ireland

***** Corresponding author

E-mail: a.forde21@nuigalway.ie

Contents

Modifications to the Empirical Bayes method **1**

Simulation details **2**

Quantitative trait with a correlation structure imposed **2**

Quantitative trait with independence assumed **4**

Binary trait with independence assumed **5**

Evaluation of performance assuming independent SNPs **8**

Derivation of the estimated MSE of significant SNPs **10**

Evaluation of performance using pruned real data sets **12**

Number of independent signals required **13**

Performance of the conditional likelihood method for true effects near genome-wide significance threshold **18**

References **20**

Modifications to the Empirical Bayes method

As mentioned in the main manuscript, our work incorporated the exploration of several variations of the empirical Bayes method. We first adapted the method by simply altering the minimum-BIC estimated basis function of the natural cubic spline so that the boundary knots were no longer designated to be the most extreme *z*-statistics. Instead, the lower boundary knot is defined as the 10th *z*-statistic when the *z*-statistics lie in increasing order while the upper boundary knot is the 10th *z*-statistic when the *z*-statistics have been arranged in decreasing order. With *z*-statistics labelled in increasing order such that $z_1 < z_2 < \cdots < z_N$, this constraint ensures that the estimated log $p(z)$ is linear beyond these boundary knots, i.e. below z_{10} and above $z_{N\text{-}10}$.

Following this, together with the above modification, the exclusion of the utilization of BIC for model selection purposes was considered which limited the number of knots in the spline. We reverted back to abiding by Efron's original specification of setting the degrees of freedom to 7 (1). The motivation for this choice stemmed from evaluating the performance of the empirical Bayes method with real data sets in which it was seen that the BIC approach generally selected a large number of basis functions, resulting in severe overfitting. The reason for this being perhaps due to these model selection criteria not accounting for the presence of strong linkage disequilibrium in real data sets.

This observation was also responsible for the investigation of models that enforced additional constraints on the shape of the estimated log density function. We assessed a variation of the empirical Bayes method in which the gam function in the R package mgcv (2) was employed. The gam function fits a generalized additive model (GAM), with smoothness estimation integrated in the fitting process. The smoothing parameters are selected by means of generalized cross-validation (GCV). Two uses of this function were investigated, one in which the distribution family was specified as poisson and the other in which the identification of the family as negative binomial took place. The negative binomial is considered to be the more realistic choice here as it accounts for the overdispersion that is typically found in this form of count data.

In addition, the scam function in the R package scam (3) was used to apply two shape constrained additive models (SCAMs) to the dataset at hand. This action imposes the restriction that only monotone increasing smooths can be attained for *z*-statistics greater than or equal to zero. Alternatively, for negative *z*-statistics, it results in smooths which are monotone decreasing.

Simulation details

Quantitative trait with a correlation structure imposed

The following steps were executed in order to obtain an estimated effect size, $\hat{\beta}_i$ and corresponding standard error, $\mathrm{se}(\hat{\beta}_i)$ for SNP i, $i = 1, \ldots, N$, for a quantitative trait in which a simple correlation structure has been imposed on the set of SNPs and the true effect sizes follow a normal distribution. N is the total number of SNPs, which has been fixed at $N = 1{,}000{,}000$ for this simulation study.

- A value is provided for π, the proportion of the total SNPs which are truly associated with the trait in question. This determines the number of effect SNPs $K = \pi \cdot N$, $1 \le K \le N$, forming a polygenic background.
- A minor allele frequency is attained for SNP i using the uniform distribution $\mathrm{maf}_i \sim U[0.01, 0.5]$.
- Let us assume that the true effect sizes are $\boldsymbol{b} = b_1, \ldots, b_N$. We then let $\boldsymbol{X}$ be an $n \times N$ matrix of genotypes, assumed to be mean centred, that is for each column i: $\sum_j X_{j,i} = 0$. Recall that n represents the sample size or total number of individuals. We assume that genotypes $\boldsymbol{X}$ affects the $n \times 1$ response matrix $\boldsymbol{Y}$ through the following linear model:

$$\boldsymbol{Y} = \boldsymbol{X}\boldsymbol{b} + \varepsilon \qquad \text{(S1)}$$

where ε is a vector of independent zero mean normally distributed errors, i.e. it is assumed that $\mathrm{var}(\varepsilon) = \sigma^2\boldsymbol{I}$ where $\boldsymbol{I}$ denotes the $n \times n$ identity matrix. Now, let $\boldsymbol{D} = \mathrm{Diag}(d_1, \ldots, d_N)$ where $d_i = \sum_j X^2_{j,i}$. Using $E(X^2_i) = \sum_j X^2_{j,i}/n$, an approximation via the Law of Large Numbers, and the identity $\mathrm{var}(X_i) = E(X^2_i) - (E(X_i))^2$, we have:

$$d_i = n \cdot \mathrm{var}(X_i) = n \cdot 2 \cdot \mathrm{maf}_i(1 - \mathrm{maf}_i) \qquad \text{(S2)}$$

as each column of genotypes is assumed to be mean centred, $E(X_i) = 0$ for each i and the variance of each SNP i, $\mathrm{var}(X_i)$, is assumed to take the form $\mathrm{var}(X_i) = 2 \cdot \mathrm{maf}_i(1-\mathrm{maf}_i)$.

With the above definition for $\boldsymbol{D}$, the regression coefficients, $\hat{\beta}_1, \ldots, \hat{\beta}_N$ from the marginal regression of Y on each SNP X_i can be computed via the following matrix equation, in which $\hat{\beta} = [\hat{\beta}_1 \; \hat{\beta}_2 \; \cdots \; \hat{\beta}_N]^T$:

$$\hat{\beta} = \boldsymbol{D}^{-1}\boldsymbol{X}^{\boldsymbol{T}}\boldsymbol{Y}\,. \qquad \text{(S3)}$$

Conditionally on the genotype matrix $\boldsymbol{X}$, these estimated regression coefficients have variance-covariance matrix:

$$\mathrm{cov}(\hat{\beta}) = \boldsymbol{D}^{-1}\boldsymbol{X}^{\boldsymbol{T}}\boldsymbol{X}\boldsymbol{D}^{-1}\sigma^2 = \boldsymbol{D}^{-\frac{1}{2}}\boldsymbol{D}^{-\frac{1}{2}}\boldsymbol{X}^{\boldsymbol{T}}\boldsymbol{X}\boldsymbol{D}^{-\frac{1}{2}}\boldsymbol{D}^{-\frac{1}{2}}\sigma^2 \approx \boldsymbol{D}^{-\frac{1}{2}}\boldsymbol{R}\boldsymbol{D}^{-\frac{1}{2}}\sigma^2 \qquad \text{(S4)}$$

where $\boldsymbol{R}$ is the $N \times N$ LD matrix of inter-genotype correlations, which should approximately equal the empirical correlation matrix $\boldsymbol{D}^{-½}\boldsymbol{X}^{\boldsymbol{T}}\boldsymbol{X}\,\boldsymbol{D}^{-½}$. Letting $\boldsymbol{SE}$ be the $N \times N$ diagonal matrix with element i equal to $\mathrm{se}(\hat{\beta}_i) = \sigma/\sqrt{\sum_{j=1}^{n} X^2_{j,i}}$, the final expression could instead be written as: $\mathbf{cov}(\hat{\beta}) = (\boldsymbol{SE})^{-\frac{1}{2}}\boldsymbol{R}(\boldsymbol{SE})^{-\frac{1}{2}}$.

Finally, these estimated associations are not necessarily unbiased for the true causal effects $b_1, \ldots, b_N$. Instead they are unbiased for:

$$E(\hat{\beta}) = \boldsymbol{D}^{-1}\boldsymbol{X}^{\boldsymbol{T}}E(\boldsymbol{Y}) = \boldsymbol{D}^{-1}\boldsymbol{X}^{\boldsymbol{T}}E(\boldsymbol{Xb} + \varepsilon) = \boldsymbol{D}^{-1}\boldsymbol{X}^{\boldsymbol{T}}\boldsymbol{Xb} = \boldsymbol{D}^{-\frac{1}{2}}\boldsymbol{D}^{-\frac{1}{2}}\boldsymbol{X}^{\boldsymbol{T}}\boldsymbol{X}\boldsymbol{D}^{-\frac{1}{2}}\boldsymbol{D}^{\frac{1}{2}}\boldsymbol{b} \approx \boldsymbol{D}^{-\frac{1}{2}}\boldsymbol{R}\boldsymbol{D}^{\frac{1}{2}}\boldsymbol{b} \quad \text{(S5)}$$

Multivariate normality of $\hat{\beta}$ is inherited from the assumption that ε is normally distributed. In summary, conditional on the centred genotype matrix $\boldsymbol{X}$:

$$\hat{\beta} \sim N\left(\boldsymbol{D}^{-\frac{1}{2}}\boldsymbol{R}\boldsymbol{D}^{\frac{1}{2}}\boldsymbol{b}, \boldsymbol{D}^{-\frac{1}{2}}\boldsymbol{R}\boldsymbol{D}^{-\frac{1}{2}}\sigma^2\right) \quad \text{(S6)}$$

We note that illustrations of derivations similar to the above can also be seen in existing literature, for example in Lloyd-Jones et al. (5).

- Assuming that the true effect sizes, b_i, $i = 1, \ldots, K$, of associated SNPs follow a Gaussian distribution with mean 0, b_i is then sampled for SNP i, $i = 1, \ldots, K$, from the distribution $b_i \sim N(0, [2\text{maf}_i(1-\text{maf}_i)])$. Non-effect SNP i, $i = K+1, \ldots, N$, is simply assigned the null true effect size, $b_i = 0$.
- Defining the heritability h^2 as the proportion of phenotypic variation, var(Y), that is explained by all SNPs, var(Y) can be computed as

$$\text{var}(Y) = \frac{\sum_{i=1}^{K} 2\text{maf}_i(1-\text{maf}_i) \cdot b_i^2}{h^2} \quad \text{(S7)}$$

and following this, the true effect sizes are scaled giving $b_i = \frac{b_i}{\sqrt{\text{var}(Y)}}$ for $i = 1, \ldots, N$. This scaling provides a phenotype with variance 1. In order to briefly illustrate why this is the case, let us consider re-defining the unscaled phenotype as Y^* and the unscaled coefficients as b_i^*. Then, the scaled effect sizes are defined as $b_i = \frac{b_i^*}{\sqrt{\text{var}(Y^*)}}$ and the scaled phenotype becomes $Y = \frac{Y^*}{\sqrt{\text{var}(Y^*)}}$ and is defined by the same equation as before but with scaled coefficients, b_i. Using Eq (S7) to provide an expression for the variance of the unscaled phenotype, var(Y^*), in which we merely change Y to Y^* and b_i^2 to $(b_i^*)^2$, it is clear to see that the variance of the scaled phenotype, var(Y), would be equal to 1.
- Next, using the R function sample, K random positions between 1 and N are chosen for the effect SNPs and the vectors containing the values of true effect size, b_i and minor allele frequency, maf_i are adjusted accordingly.
- In order to reduce computation time, it is assumed that the same linkage disequilibrium structure exists in independent blocks of 100 SNPs. Therefore, for each block of 100 SNPs, the estimated effect sizes, $\hat{\beta}_i$ are simulated using Eq (S6). As we have already scaled b_i to ensure the phenotype has variance 1, we have $\sigma^2 = 1$. The matrix $\boldsymbol{D}$ is a diagonal 100 × 100 matrix. Thus, using Eq (S2), $\boldsymbol{D}^{-1/2}$ is a similar diagonal matrix with $d_i^{-1/2} = \frac{1}{\sqrt{n \cdot 2 \cdot \text{maf}_i(1-\text{maf}_i)}}$ and $\boldsymbol{D}^{1/2}$ has diagonal entries $d_i^{1/2} = \sqrt{n \cdot 2 \cdot \text{maf}_i(1-\text{maf}_i)}$. The challenge here is to choose a suitable matrix for $\boldsymbol{R}$, the 100 × 100 LD matrix of inter-genotype correlations. For simplicity, we have chosen $\boldsymbol{R}$ to be of the following format:

$$\boldsymbol{R} = \begin{pmatrix} 1 & \rho & \rho^2 & \rho^3 & \cdots & \rho^{99} \\ \rho & 1 & \rho & \rho^2 & \cdots & \rho^{98} \\ \rho^2 & \rho & 1 & \rho & \cdots & \rho^{97} \\ \vdots & \vdots & \vdots & \vdots & \ddots & \vdots \\ \rho^{99} & \rho^{98} & \rho^{97} & \rho^{96} & \cdots & 1 \end{pmatrix} \quad \text{(S8)}$$

The task is now to choose a suitable value for ρ. In Bosch et al. (6), it can be seen in Figure 1a that the average r^2 at a distance of 5,000 bases is just over 0.4 in Europeans. Using our UKBB data set, we computed the median distance between SNPs and obtained a value of 189 bases. This would suggest that there exist an average of approximately 26 SNPs per 5,000 bases and thus, a rough estimate of an appropriate value for ρ may be computed as follows:

$$\rho^{26} \approx \sqrt{0.4} \approx 0.63245 \rightarrow \rho \approx 0.9825. \quad \text{(S9)}$$

Within each block of 100 SNPs, we then simulated values for $E(\hat{\beta})$, as defined by Eq (S5), and $\hat{\beta}$. We obtained $\hat{\beta}$ using the R function rnorm with $\hat{\beta} = E(\hat{\beta}) + \boldsymbol{R}^{\frac{1}{2}}\boldsymbol{D}^{-\frac{1}{2}} \cdot$ rnorm(100). The standard errors for each SNP, $\text{se}(\hat{\beta}_i)$ were easily obtained from the diagonal entries of $\boldsymbol{D}^{-½}$.

- The above process thus provided values for $E(\hat{\beta}_i), \hat{\beta}_i$ and $\text{se}(\hat{\beta}_i)$ for each SNP i = 1, ..., N, in which the same LD structure described by the simple matrix $\boldsymbol{R}$ has been imposed for each independent block of 100 SNPs.

Quantitative trait with independence assumed

Similar steps were followed in order to obtain an estimated effect size, $\hat{\beta}_i$ and corresponding standard error, $\text{se}(\hat{\beta}_i)$ for SNP i, i = 1, ..., N, in which a quantitative trait was considered, it was assumed that SNPs were independent and effect sizes follow a normal distribution.

1) A polygenic background of $K = \pi \cdot N$, $1 \leq K \leq N$, effect SNPs is formed, in which π is the proportion of the total SNPs which are truly associated with the trait in question.
2) A minor allele frequency is attained for SNP i using the uniform distribution $\text{maf}_i \sim U[0.01, 0.5]$.
3) Assuming that the true effect sizes, β_i of associated SNPs follow a Gaussian distribution with mean 0, β_i is sampled for SNP i, i = 1, ..., K, from the distribution $\beta_i \sim \sim N(0, [2\text{maf}_i(1- \text{maf}_i)])$. Non-effect SNP i, $i = K$+1, ..., N, is simply assigned the null true effect size, $\beta_i = 0$.
4) Defining the heritability h^2 as the proportion of phenotypic variation, var(Y), that is explained by all SNPs, var(Y) is computed in the same manner as Eq (S7) but with b_i replaced by β_i. Following this, the true effect sizes are scaled by dividing each by the square root of var(Y) in order to ensure a phenotype with variance 1.
5) For a single SNP i, it is assumed that the underlying relationship between y_j, a numerical measurement of the trait of individual j and $x_j \in \{0,1,2\}$, the number of minor alleles that individual j has at SNP i, is described by the simple linear model

$$y_j = \beta_0 + \beta_1 x_j + \varepsilon_j \tag{S10}$$

for j = 1, ..., n. In this equation, β_1 is recognised as the effect size of SNP i, i.e. $\beta_1 = \beta_i$. Using the properties of a linear model, we obtain

$$\text{se}(\hat{\beta}_i) = \sqrt{\frac{1 - 2\text{maf}_i(1 - \text{maf}_i)\ \cdot\ \beta_i^2}{(n-2)\cdot 2\text{maf}_i(1 - \text{maf}_i)}} \tag{S11}$$

for each SNP i, i = 1, ..., N.

6) Finally, assuming that the effect size of each SNP follows a Gaussian distribution with mean β_i and standard deviation $\text{se}(\hat{\beta}_i)$, an estimated effect size, $\hat{\beta}_i$ is simulated for each SNP i, i = 1, ..., N, i.e. $\hat{\beta}_i \sim N\left(\beta_i,\ \text{se}(\hat{\beta}_i)\right)$.

For a bimodal distribution of effect sizes, summary statistics are simulated in the exact same manner but with step 3) above replaced by:

3) It is assumed that the true effect sizes, β_i of half of the associated SNPs follow a Gaussian distribution with mean 2.5 while the true effect sizes, β_i of the other half follow a Gaussian distribution with mean 0. Thus, β_i is sampled for SNP i, i = 1, ..., $K/2$, from the distribution β_i ~ ~ N(2.5, [2maf$_i$(1- maf$_i$)]) and for SNP i, i = $K/2$ + 1, ..., K, β_i is sampled from the distribution β_i ~ ~ N(0, [2maf$_i$(1- maf$_i$)]). As above, non-effect SNP i, i = K+1, ..., N, is assigned the null true effect size, β_i = 0.

Similarly, for a skewed distribution of effect sizes, step 3) is altered and takes the form of:

3) It is assumed that the true effect sizes, β_i of 10% of the associated SNPs follow a negative exponential distribution with rate ([2maf$_i$(1- maf$_i$)])$^{-1/2}$ while the true effect sizes, β_i of the other 90% follow an exponential distribution with the same rate. Thus, β_i is sampled for SNP i, i = 1, ..., $K/10$, from the distribution $\beta_i \sim -\text{Exp}\left(\frac{1}{\sqrt{2\text{maf}_i(1-\text{maf}_i)}}\right)$ and for SNP i, i = $K/10$ + 1, ..., K, β_i is sampled from the distribution $\beta_i \sim \text{Exp}\left(\frac{1}{\sqrt{2\text{maf}_i(1-\text{maf}_i)}}\right)$. As above, non-effect SNP i, i = K+1, ..., N, is assigned the null true effect size, β_i = 0.

Binary trait with independence assumed

Maintaining a normal effect size distribution and a set of independent SNPs, the following steps were executed in order to obtain an estimated effect size, $\hat{\beta}_i$ and corresponding standard error, $\text{se}(\hat{\beta}_i)$ for SNP i, i = 1, ..., N, for a binary trait with disease prevalence of 0.1.

1) A polygenic background of $K = \pi \cdot N$, $1 \le K \le N$, effect SNPs is formed, in which π is the proportion of the total SNPs which are truly associated with the trait in question.
2) A minor allele frequency is attained for SNP i using the uniform distribution maf$_i$ ~ U[0.01, 0.5].
3) Assuming that the true effect sizes, β_i of associated SNPs follow a Gaussian distribution with mean 0, β_i is sampled for SNP i, i = 1, ..., K, from the distribution β_i

~ ~ $N(0, [2\text{maf}_i(1-\text{maf}_i)])$. Non-effect SNP i, $i = K+1, ..., N$, is simply assigned the null true effect size, $\beta_i = 0$.

4) As demonstrated by Yi and Zhi (7), using the latent-data formulation of the logistic regression, the proportion of the latent-data variance explained by the variants, i.e. the liability heritability, can be computed as:

$$h^2 = \frac{\sum_{i=1}^{k} 2\text{maf}_i(1-\text{maf}_i) \cdot \beta_i^2}{1.6^2 + \sum_{i=1}^{k} 2\text{maf}_i(1-\text{maf}_i) \cdot \beta_i^2} \tag{S12}$$

This provides an expression for the heritability h^2 of the binary trait and thus, the true effect sizes are re-scaled giving

$$\beta_i = \beta_i \cdot \sqrt{\frac{1.6^2 h^2}{(1-h^2)\sum_{i=1}^{k} 2\text{maf}_i(1-\text{maf}_i) \cdot \beta_i^2}} \tag{S13}$$

for $i = 1, ..., N$.

5) For a single SNP i, it is assumed that the underlying relationship between y_j, a numerical measurement of the trait of individual j and $x_j \in \{0,1,2\}$, the number of minor alleles that individual j has at SNP i, is described by the logistic model:

$$\text{logit}(\text{P}(y_j = 1|x_j)) = \beta_0 + \beta_1 x_j + \varepsilon_j \tag{S14}$$

for $j = 1, ..., n$. In this equation, β_1 is recognised as the effect size of SNP i, i.e. $\beta_1 = \beta_i$. For SNP i, as we have simulated a value for both its minor allele frequency maf_i and true effect size $\beta_i = \beta_1$, we can obtain a corresponding value for β_0 using the fact that we have chosen the disease prevalence to be 0.1, i.e. $\text{P}(Y = 1) = 0.1$. Therefore, as $\text{P}(Y = 1) = \sum \text{P}(Y = 1 \mid X)\,\text{P}(X)$ and $\text{P}(Y = 1|X) = \frac{e^{\beta_0+\beta_1 X}}{1+ e^{\beta_0+\beta_1 X}}$, for SNP i, we have:

$$\text{maf}_i^2 \cdot \frac{e^{\beta_0+2\beta_1}}{1+e^{\beta_0+2\beta_1}} + 2\text{maf}_i(1-\text{maf}_i) \cdot \frac{e^{\beta_0+\beta_1}}{1+e^{\beta_0+\beta_1}} + (1-\text{maf}_i)^2 \cdot \frac{e^{\beta_0}}{1+e^{\beta_0}} = 0.1 \tag{S15}$$

Solving this equation then provides a value for β_0, given values for maf_i and β_1. Now, the aim is to obtain a value for $\text{se}(\hat{\beta}_i) = \text{se}(\hat{\beta}_1)$ for each SNP i. Firstly, let us denote the maximum likelihood estimated logistic regression coefficient vector, from regressing the $n \times 1$ response vector $\boldsymbol{Y}$ on the $n \times 1$ genotype vector $\boldsymbol{X}$, as $\widehat{\boldsymbol{\beta}} = \begin{pmatrix}\hat{\beta}_0 \\ \hat{\beta}_1\end{pmatrix}$, which can be represented as:

$$\widehat{\boldsymbol{\beta}} = \left(\boldsymbol{X}^T \widehat{\boldsymbol{W}} \boldsymbol{X}\right)^{-1} \boldsymbol{X}(\boldsymbol{Y} - \widehat{\boldsymbol{p}}) \tag{S16}$$

Here,

$$\widehat{\boldsymbol{W}} = \begin{pmatrix} \hat{p}_1(1-\hat{p}_1) & 0 & \cdots & 0 \\ 0 & \hat{p}_2(1-\hat{p}_2) & \cdots & 0 \\ \vdots & \vdots & \ddots & \vdots \\ 0 & 0 & \cdots & \hat{p}_n(1-\hat{p}_n) \end{pmatrix} \tag{S17}$$

$$\hat{p}_j = \frac{e^{\hat{\beta}_0+\hat{\beta}_1 X_j}}{1+e^{\hat{\beta}_0+\hat{\beta}_1 X_j}} \tag{S18}$$

and

$$\hat{\boldsymbol{p}} = (\hat{p}_1, \dots, \hat{p}_n)^T \tag{S19}$$

It is well known that var($\hat{\boldsymbol{\beta}}$) ~ $(\boldsymbol{X^T}\widehat{\boldsymbol{W}}\boldsymbol{X})^{-1}$. This can be re-expressed as:

$$\boldsymbol{X^T}\widehat{\boldsymbol{W}}\boldsymbol{X} = \begin{pmatrix} \sum_{j=1}^{n} \hat{p}_j(1-\hat{p}_j) & \sum_{j=1}^{n} X_j\hat{p}_j(1-\hat{p}_j) \\ \sum_{j=1}^{n} X_j\hat{p}_j(1-\hat{p}_j) & \sum_{j=1}^{n} X_j^2\hat{p}_j(1-\hat{p}_j) \end{pmatrix}$$
$$\sim (n)\begin{pmatrix} \mathrm{E}(P(1-P)) & \mathrm{E}(GP(1-P)) \\ \mathrm{E}(GP(1-P)) & \mathrm{E}(G^2P(1-P)) \end{pmatrix} \tag{S20}$$

where P is the random variable that takes value $\mathrm{P}(Y=1|X) = \frac{e^{\beta_0+\beta_1 X}}{1+e^{\beta_0+\beta_1 X}}$ for genotype X, and X is the binomial distribution for genotype assuming Hardy Weinberg Equilibrium. Therefore, using the above, we obtain the inverse of $\boldsymbol{X^T}\widehat{\boldsymbol{W}}\boldsymbol{X}$. Taking the square root of the value in the second row and second column of this matrix provides $\mathrm{se}(\hat{\beta}_1)$. This process is repeated for each SNP i, i = 1, ..., N.

6) Finally, assuming that the effect size of each SNP follows a Gaussian distribution with mean β_i and standard deviation $\mathrm{se}(\hat{\beta}_i)$, an estimated effect size, $\hat{\beta}_i$ is simulated for each SNP i, i = 1, ..., N, i.e. $\hat{\beta}_i \sim N\left(\beta_i,\ \mathrm{se}(\hat{\beta}_i)\right)$.

Evaluation of performance assuming independent SNPs

Surprisingly, in this instance in which SNPs are assumed to be independent, with respect to the evaluation metric 'change in RMSE of significant SNPs' the simulations suggest that many of the investigated *Winner's Curse* methods tend to break down, or no longer make improvements when the proportion of effect SNPs is 0.001. In these simulations, a replication approach, which selects significant SNPs using a discovery GWAS and then employs a replication GWAS of the same size to obtain unbiased association estimates for these SNPs, was also considered. This can be viewed as acting as a form of benchmark for the other methods. Both the empirical Bayes method and 'EB-gam-nb' perform very similar to this replication approach, as can be seen in S8 Fig. Under the assumption that SNPs are independent, this observation supports the use of these two methods to adjust for *Winner's Curse* bias, particularly when a replication GWAS is not available. The consistency of the methods is what makes them stand out. Unlike the bootstrap, FIQT and other variations of the empirical Bayes method, applying 'EB' or 'EB-gam-nb' rarely results in an increase in the RMSE over all significant SNPs.

It is surprising to see the empirical Bayes method which uses two shape constrained additive models (SCAMs) perform extremely poorly when the sample size is 300,000 and the proportion of effect SNPs 0.001. This method was seen to perform well with real data and when a correlation structure was imposed on simulated data. When the proportion of effect SNPs is 0.01, the proposed bootstrap method for summary statistics performs in a comparable manner to both empirical Bayes and the replication method. However, the bootstrap method ceases to perform as well at reducing the RMSE of association estimates of significant SNPs when the proportion of effect SNPs is reduced to 0.001. That said, it is still seen to perform competitively with respect to other currently published methods. Just as was observed in the simulations with linkage disequilibrium, described in the main manuscript, the conditional likelihood methods tend to perform poorly compared to the other methods. The average percentage improvement in estimated RMSE across all scenarios was 10% for 'EB-gam-nb' and 16.2% for 'EB', although this average metric was negative for some methods, such as the conditional likelihood based approaches, indicating increased inaccuracy from applying *Winner's Curse* corrections.

Imposing a threshold of 5×10^{-4}, it is still the original empirical Bayes method which seems to be the best, as can be seen in S9 Fig. For the larger sample size of 300,000, it is worth noting that the other four variations of the empirical Bayes method do not seem to perform well compared to other methods, with a positive value for change in RMSE noted in some cases. When the proportion of effect SNPs is 0.001, 'EB-gam-nb' is not behaving as well as seen previously.

In addition to the above, summary statistics were simulated for a quantitative trait in which the effect sizes followed bimodal or skewed distributions and for a binary trait with a normal effect size distribution. For these supplementary investigations, in order to reduce computational burden, the assumption of independent SNPs was maintained and our suggested variations of the empirical Bayes method were excluded from evaluation. The results from assessing the methods using estimated change in RMSE of significant SNPs can be seen in S10-S15 Figs. Focusing on the 5×10^{-8} threshold, very similar conclusions may be

deduced for the three different situations considered. Firstly, the extreme unreliability of the conditional likelihood methods is again evident. Out of the six methods evaluated, the empirical Bayes method is clearly the most consistent at reducing the RMSE of association estimates for significant SNPs. In S10, S12 and S14 Figs, it can be seen to perform in a similar manner to the method which obtains the estimated effect sizes of significant SNPs in the discovery GWAS from an independent replication GWAS with a similar number of samples. Its dominance over the other correction methods is most noticeable in the depiction of results corresponding to a quantitative trait in which the effect sizes of SNPs have a skewed distribution. Across all situations, both FIQT and the bootstrap method tend to behave poorly when the proportion of effect SNPs is 0.001 but show improved performances when this proportion is increased to 0.01. However, under the assumption of a skewed effect size distribution, this improvement by the two methods is no longer observed, often resulting in positive values for the estimated change in RMSE.

Derivation of the estimated MSE of significant SNPs

In order to evaluate the *Winner's Curse* correction methods using real data sets, we chose to compute the estimated MSE of N_{sig} significant SNPs with respect to each method and discovery GWAS. The following provides a derivation of the expression for this estimated MSE which is given in Eq (14) in the main manuscript.

- The true empirical MSE over all significant SNPs is $\frac{1}{N_{\text{sig}}}\sum_{i=1}^{N_{\text{sig}}}\left(\hat{\beta}_{\text{disc,adj},i}-\beta_i\right)^2$, in which $\hat{\beta}_{\text{disc,adj},i}$ is the adjusted/corrected version of the estimated effect size for SNP i obtained in the discovery study and β_i is the true effect size of that SNP. Unfortunately, in contrast to the simulation study, the value of the true effect size for each SNP is unknown. However, a replication study has provided an independent estimated effect size for each SNP, $\hat{\beta}_{\text{rep},i}$. These estimated effect sizes, $\hat{\beta}_{\text{rep},i}$, are considered to be unbiased estimates of their corresponding true effect sizes, i.e. $\text{E}\left[\hat{\beta}_{\text{rep},i}\right]=\beta_i$, i = 1, ..., N_{sig}.
- Therefore, let us reconstruct the equation, $\frac{1}{N_{\text{sig}}}\sum_{i=1}^{N_{\text{sig}}}\left(\hat{\beta}_{\text{disc,adj},i}-\hat{\beta}_{\text{rep},i}\right)^2$, in order to obtain an expression that approximates the empirical MSE over all significant SNPs:

$$\begin{aligned}\frac{1}{N_{\text{sig}}}\sum_{i=1}^{N_{\text{sig}}}\left(\hat{\beta}_{\text{disc,adj},i}-\hat{\beta}_{\text{rep},i}\right)^2 &= \frac{1}{N_{\text{sig}}}\sum_{i=1}^{N_{\text{sig}}}\left[\left(\hat{\beta}_{\text{disc,adj},i}-\beta_i+\beta_i-\hat{\beta}_{\text{rep},i}\right)^2\right]\\ &= \frac{1}{N_{\text{sig}}}\sum_{i=1}^{N_{\text{sig}}}\left[\left(\hat{\beta}_{\text{disc,adj},i}-\beta_i\right)^2\right]-2\frac{1}{N_{\text{sig}}}\sum_{i=1}^{N_{\text{sig}}}\left[\left(\hat{\beta}_{\text{disc,adj},i}-\beta_i\right)\left(\beta_i-\hat{\beta}_{\text{rep},i}\right)\right]\\ &\quad+\frac{1}{N_{\text{sig}}}\sum_{i=1}^{N_{\text{sig}}}\left[\left(\beta_i-\hat{\beta}_{\text{rep},i}\right)^2\right]\end{aligned} \qquad \text{(S21)}$$

The second term here is approximately 0 due to the fact that the discovery and replication estimates are independent and the replication estimate is considered to be unbiased, $\text{E}\left[\hat{\beta}_{\text{rep},i}\right]=\beta_i$. This is illustrated below:

$$\begin{aligned}\frac{1}{N_{\text{sig}}}\sum_{i=1}^{N_{\text{sig}}}\left[\left(\hat{\beta}_{\text{disc,adj},i}-\beta_i\right)\left(\beta_i-\hat{\beta}_{\text{rep},i}\right)\right] &\approx \text{E}\left[\frac{1}{N_{\text{sig}}}\sum_{i=1}^{N_{\text{sig}}}\left[\left(\hat{\beta}_{\text{disc,adj},i}-\beta_i\right)\left(\beta_i-\hat{\beta}_{\text{rep},i}\right)\right]\right]\\ &= \frac{1}{N_{\text{sig}}}\sum_{i=1}^{N_{\text{sig}}}\text{E}\left[\left(\hat{\beta}_{\text{disc,adj},i}-\beta_i\right)\left(\beta_i-\hat{\beta}_{\text{rep},i}\right)\right]=0\end{aligned} \qquad \text{(S22)}$$

since $\text{E}\left[\left(\hat{\beta}_{\text{disc,adj},i}-\beta_i\right)\left(\beta_i-\hat{\beta}_{\text{rep},i}\right)\right]=\text{E}\left(\hat{\beta}_{\text{disc,adj},i}-\beta_i\right)\text{E}\left(\beta_i-\hat{\beta}_{\text{rep},i}\right)=0$.
In addition, the third term may be approximated as:

$$\frac{1}{N_{\text{sig}}}\sum_{i=1}^{N_{\text{sig}}}\left[\left(\beta_i-\hat{\beta}_{\text{rep},i}\right)^2\right]\approx\frac{1}{N_{\text{sig}}}\sum_{i=1}^{N_{\text{sig}}}\text{E}\left[\left(\beta_i-\hat{\beta}_{\text{rep},i}\right)^2\right]\approx\frac{1}{N_{\text{sig}}}\sum_{i=1}^{N_{\text{sig}}}\left(\widehat{\text{se}\left(\hat{\beta}_{\text{rep},i}\right)}\right)^2 \qquad \text{(S23)}$$

- Rearranging the final expression provides us with a metric, namely the estimated MSE over all significant SNPs, approximating the empirical MSE:

$$\frac{1}{N_{\text{sig}}}\sum_{i=1}^{N_{\text{sig}}}\left[\left(\hat{\beta}_{\text{disc,adj},i}-\beta_i\right)^2\right] \approx \frac{1}{N_{\text{sig}}}\sum_{i=1}^{N_{\text{sig}}}\left(\hat{\beta}_{\text{disc,adj},i}-\hat{\beta}_{\text{rep},i}\right)^2 - \frac{1}{N_{\text{sig}}}\sum_{i=1}^{N_{\text{sig}}}\left(\widehat{\text{se}\left(\hat{\beta}_{\text{rep},i}\right)}\right)^2 \tag{S24}$$

Note that our estimated MSE, $\frac{1}{N_{\text{sig}}}\sum_{i=1}^{N_{\text{sig}}}\left(\hat{\beta}_{\text{disc,adj},i}-\hat{\beta}_{\text{rep},i}\right)^2 - \frac{1}{N_{\text{sig}}}\sum_{i=1}^{N_{\text{sig}}}\left(\widehat{\text{se}\left(\hat{\beta}_{\text{rep},i}\right)}\right)^2$, and the true empirical MSE, $\frac{1}{N_{\text{sig}}}\sum_{i=1}^{N_{\text{sig}}}\left(\hat{\beta}_{\text{disc,adj},i}-\beta_i\right)^2$ *both* have the same expectation: $\frac{1}{N_{\text{sig}}}\text{E}\left[\sum_{i=1}^{N_{\text{sig}}}\left(\hat{\beta}_{\text{disc,adj},i}-\beta_i\right)^2\right]$.

Evaluation of performance using pruned real data sets

Due to the issues mentioned in the main manuscript regarding the performance of methods in the presence of linkage disequilibrium in real data sets, we also explored applying the methods to data sets of pruned SNPs. PLINK 2.0 (4) was used to prune the original set of SNPs. The command '--indep-pairwise 50 5 0.5' was employed for pruning. This meant that pruning occurred by first calculating LD between each pair of SNPs in a window of 50 SNPs. If an LD value greater than 0.5 was observed, then one SNP out of this pair was removed. The window was shifted 5 SNPs forward and the process was repeated. 1,589,295 SNPs remained after this procedure, a data set about 20% of the size of the original.

Due to this great reduction in the total number of SNPs in each of the six data sets, the number of significant SNPs that passed the two thresholds, 5×10^{-8} and 5×10^{-4}, naturally also decreased for each data set. For example, at the significance threshold 5×10^{-8}, the number of significant SNPs for the first BMI data set fell from 6,908 to 439 while for the first height data set, 5859 significant SNPs were obtained in comparison to the original 70,020. With respect to T2D, only two and four SNPs were deemed significant for the first and second data set, respectively. Despite these observations, the proportions of significant SNPs with smaller replication estimates and the proportions of significant SNPs that were significantly overestimated remained at similar values for the most part.

Similar to the approach described in the main manuscript, the correction methods were applied and subsequently evaluated using the estimated MSE over all significant SNPs. S15 and S16 Tables, together with S21 and S22 Figs, illustrate the results obtained. Overall, these results are extremely similar to those which use the original unpruned set of SNPs. The most obvious difference is seen for the T2D datasets when a threshold of 5×10^{-8} is imposed. Most methods now have lower estimated MSE values than that of the naïve approach, which is encouraging. However, it must be noted that these calculations are based on a very small number of significant SNPs. At this 5×10^{-8} threshold, 'EB', 'EB-gam-nb' and FIQT were the most reliable correction methods, producing an average improvement in estimated MSE of greater than 50% across the six data sets. With respect to the 5×10^{-4} significance threshold, all correction methods, apart from the conditional likelihood methods, resulted in an average improvement in estimated MSE of between 71% and 77%. 'EB-scam' provided the greatest average improvement of approximately 77%. In conclusion, evaluating the methods using a pruned set of SNPs failed to produce any major additional insights for our study. That said, it perhaps reinforced the concept that the performance of Winner's Curse correction methods should not be considered reliable if there is a very low number of independent signals in the set of SNPs passing the chosen significance threshold.

Number of independent signals required

As detailed in the main manuscript, the evaluation of the Winner's Curse correction methods using estimated MSE with a significance threshold of 5×10^{-8} and the T2D data sets highlighted the importance of ensuring that the set of significant SNPs contain enough independent signals before applying the methods. We note that this issue mainly pertains to methods that utilize the collective distribution of SNP effect sizes, either explicitly or implicitly, namely the empirical Bayes methods, the bootstrap method and FIQT. In this section, we describe our attempt at determining the number of independent signals required to ensure appropriate performance of these methods.

Using the data sets of BMI and height, we first computed the estimated MSE for each correction method at increased significance thresholds. S23 Fig illustrates the results obtained for the BMI data sets at thresholds 5×10^{-10}, 5×10^{-12} and 5×10^{-14}. The number of significant SNPs passing the specified threshold is noted in the figure. For the first BMI data set, the initial 'breakdown' of methods, i.e. methods starting to perform worse than the naïve approach of using no correction, seems to occur at 5×10^{-10} while for the second, a similar observation can be made at 5×10^{-12}. These thresholds correspond to 3,333 and 2,745 significant SNPs, respectively. Corresponding Manhattan plots, S25 and S26 Figs, have been produced using the R package qqman (8), to assist in deducing the number of independent signals contained in these two sets of significant SNPs. Note that the blue line in each Manhattan plot represents the mentioned significance threshold. In a similar manner, S24 Fig depicts the results of method evaluation using the height data sets at thresholds 5×10^{-32}, 5×10^{-34} and 5×10^{-36}. Concentrating on the empirical Bayes methods 'EB', 'EB-gam-nb' and 'EB-scam', the bootstrap method and FIQT, it seems that 'breakdown' begins to take place at 5×10^{-32} for the first height data set and at 5×10^{-34} for the second. 3,459 SNPs have been deemed significant at this 5×10^{-32} threshold while at 5×10^{-34} in the other data set, there are 3,358 significant SNPs. Manhattan plots for height can be viewed in S27 and S28 Figs. The four Manhattan plots suggest that these mentioned sets of significant SNPs are each comprised of approximately 30 independent signals.

This observation provides us with some empirical evidence, at least with respect to UKBB data, regarding how many independent significant signals are required in order to guarantee confidence in method application. Therefore, we can state that given our experience described here with real data, we would anticipate that for modern high-density genotyping arrays like UKBB, one would need hundreds of significant SNPs, possibly close to 3500, originating from perhaps at least 30 separate genomic regions to apply the methods reliably. For older arrays, where there is less LD, or for LD-pruned arrays, 30 significant SNPs representing 30 significant independent signals would be potentially sufficient.

S23 Fig. Estimated MSE of significant SNPs at thresholds 5×10^{-10}, 5×10^{-12} and 5×10^{-14} for each method and BMI data set. The estimated MSE obtained for the naïve approach is represented by the darker green bar as well as the dashed black line. The number of significant SNPs obtained for each data set, at the specified threshold, is included.

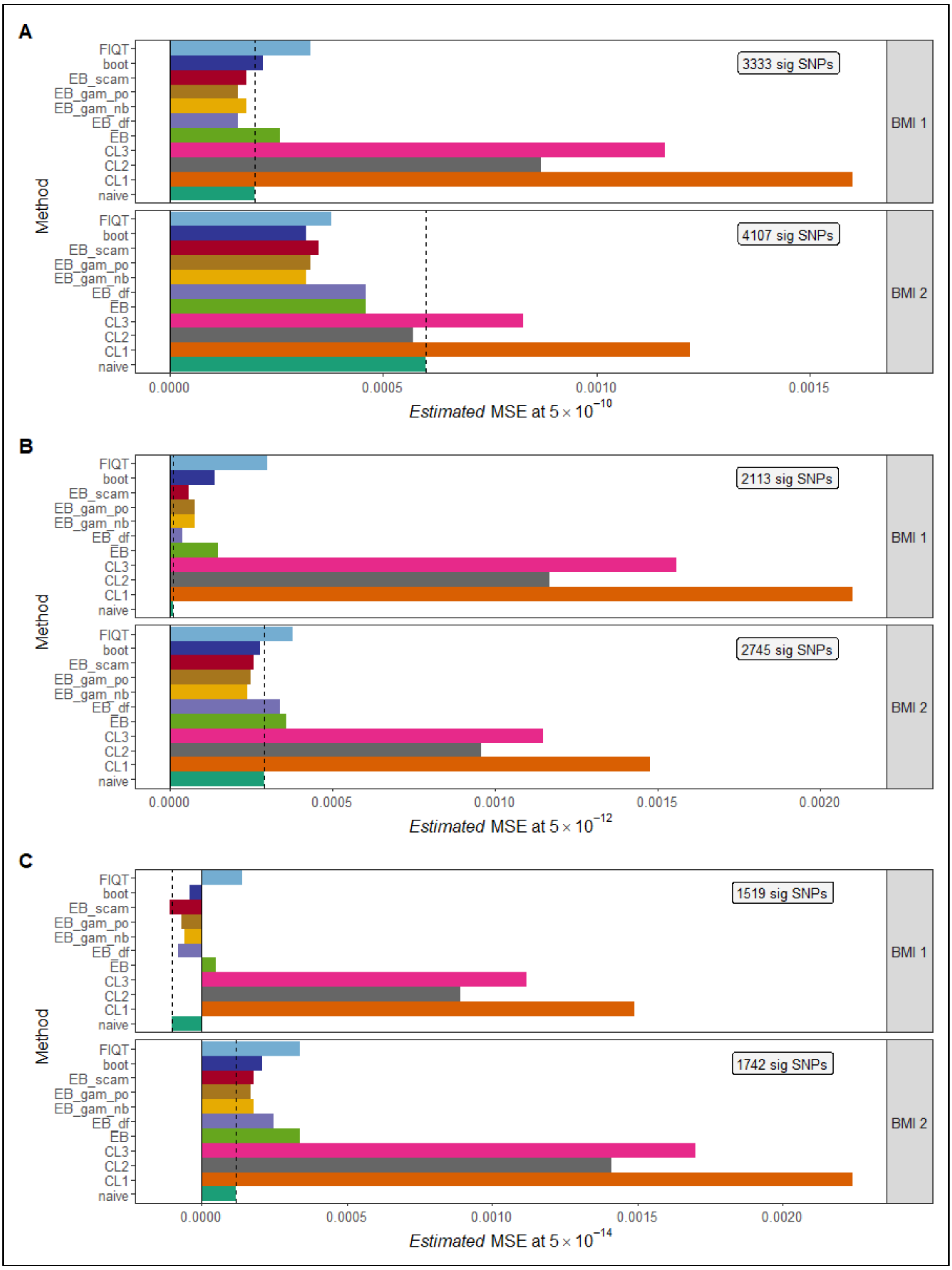

S24 Fig. Estimated MSE of significant SNPs at thresholds 5 × 10^{-32}, 5 × 10^{-34} and 5 × 10^{-36} for each method and height data set. The estimated MSE obtained for the naïve approach is represented by the darker green bar as well as the dashed black line. The number of significant SNPs obtained for each data set, at the specified threshold, is included.

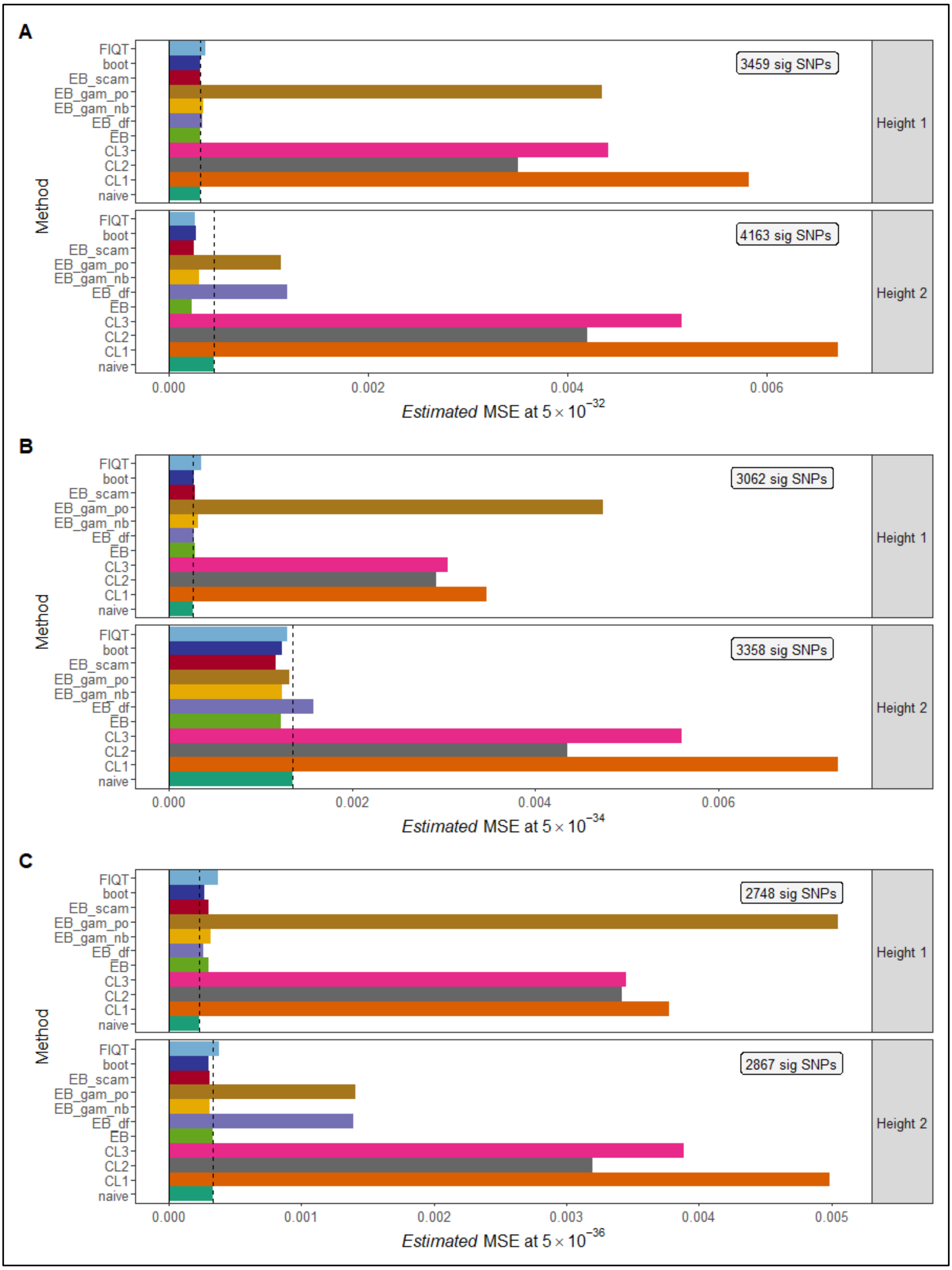

S25 Fig. Manhattan plot for the first BMI data set. The chromosome and position of each SNP with a *p*-value less than 0.05 (x-axis) is plotted against $-\log_{10}$ of its *p*-value (y-axis), for the first BMI data set. The red line represents the genome-wide significance threshold of 5×10^{-8}, while the blue line represents a 5×10^{-10} threshold.

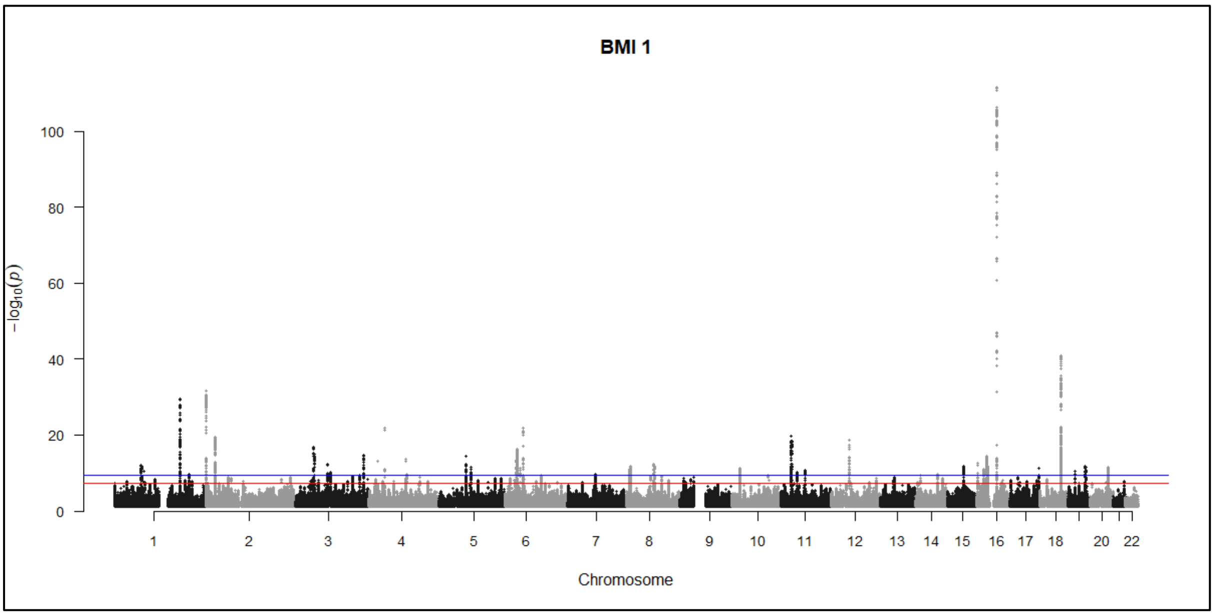

S26 Fig. Manhattan plot for the second BMI data set. The chromosome and position of each SNP with a *p*-value less than 0.05 (x-axis) is plotted against $-\log_{10}$ of its *p*-value (y-axis), for the second BMI data set. The red line represents the genome-wide significance threshold of 5×10^{-8}, while the blue line represents a 5×10^{-12} threshold.

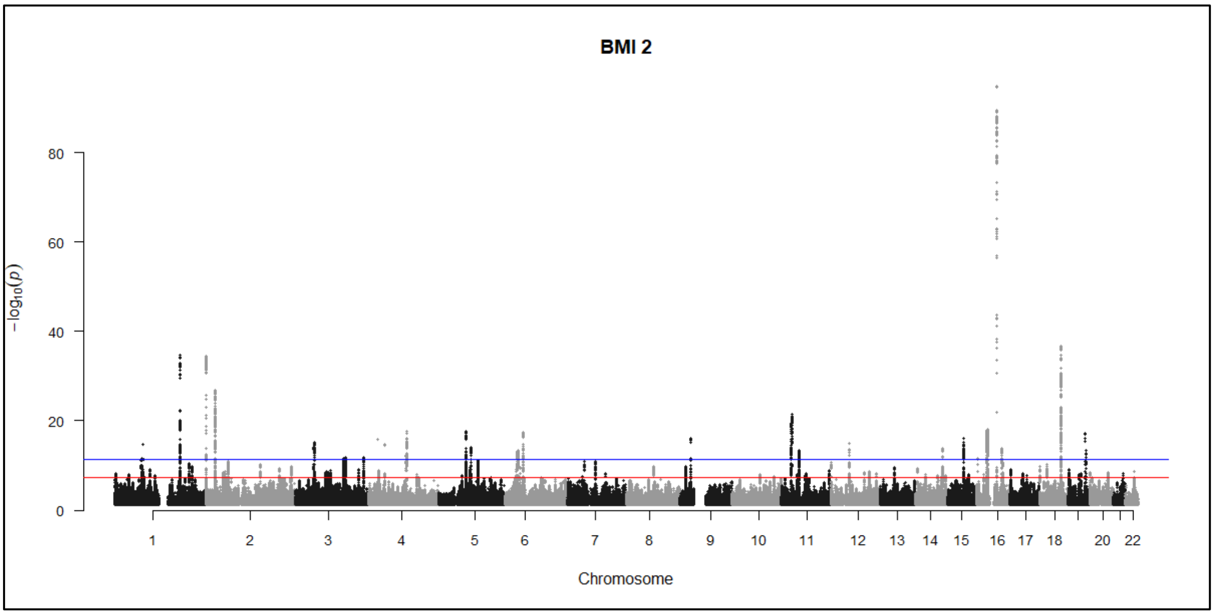

S27 Fig. Manhattan plot for the first height data set. The chromosome and position of each SNP with a *p*-value less than 0.05 (x-axis) is plotted against $-\log_{10}$ of its *p*-value (y-axis), for the first height data set. The red line represents the genome-wide significance threshold of 5×10^{-8}, while the blue line represents a 5×10^{-32} threshold.

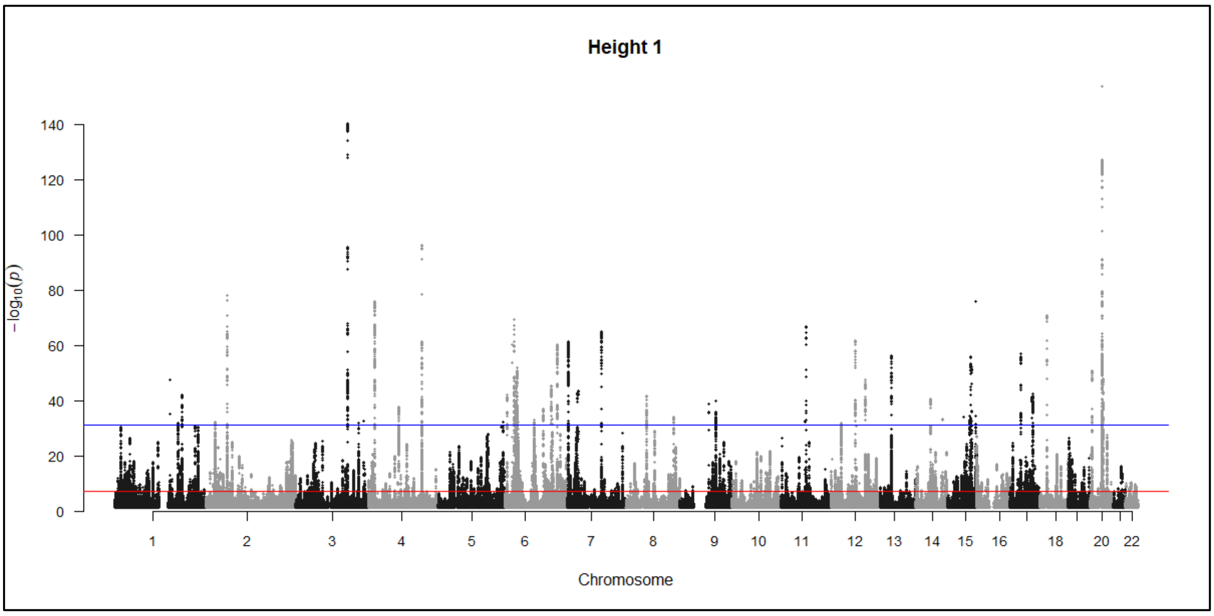

S28 Fig. Manhattan plot for the second height data set. The chromosome and position of each SNP with a *p*-value less than 0.05 (x-axis) is plotted against $-\log_{10}$ of its *p*-value (y-axis), for the first height data set. The red line represents the genome-wide significance threshold of 5×10^{-8}, while the blue line represents a 5×10^{-34} threshold.

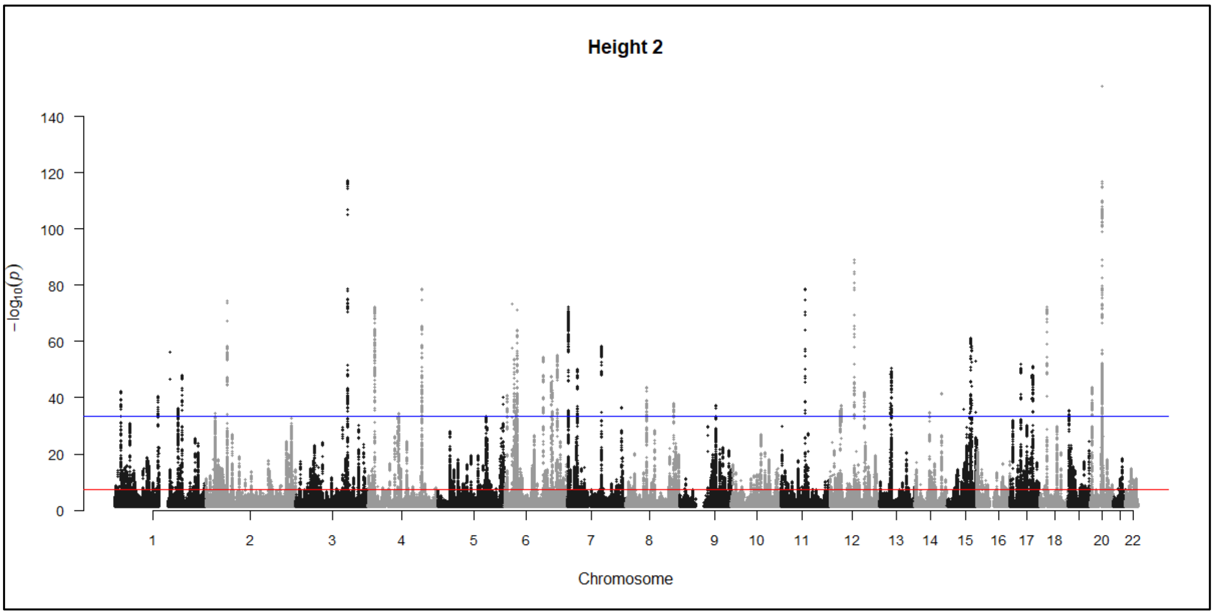

Performance of the conditional likelihood method for true effects near genome-wide significance threshold

In the simulation study discussed in the main manuscript, the conditional likelihood methods were seen to perform poorly, especially when the genome-wide significance threshold of 5×10^{-8} was used. In most instances, these methods resulted in an increase in RMSE in comparison to the RMSE obtained using the naïve approach of no correction. We hypothesized that in addition to an increase in variance, the reason for this observation was due to the overcorrection of estimated effect sizes, especially those close to the significance threshold. We chose to explore this concept further as follows.

Firstly, we considered a set of 81 equally spaced true standardized effect sizes, μ between 4 and 8. For each of these true effect sizes, we simulated 10,000 corresponding estimated effect sizes, z from the distribution $z \sim N(\mu, 1)$. Selection was imposed on these estimated effect sizes by only keeping those that were considered significant at the genome-wide significance threshold of 5×10^{-8}, i.e. had p-values less than 5×10^{-8}. The first conditional likelihood method, denoted by 'CL1' in the manuscript, was applied to the remaining estimated effect sizes, z, producing the conditional maximum likelihood estimator, $\tilde{\mu}$ for each z. The conditional bias, standard error and RMSE for 'CL1' at each value of μ was then estimated as follows, in which N_{sig} is the number of significant estimated effect sizes, z for that value of μ:

$$\widehat{\text{bias}}(\mu) = \frac{1}{N_{\text{sig}}} \sum_{i=1}^{N_{\text{sig}}} [\tilde{\mu}_i - \mu]$$
$$\widehat{\text{se}}(\mu) = \sqrt{\frac{1}{N_{\text{sig}}} \sum_{i=1}^{N_{\text{sig}}} (\tilde{\mu}_i - \overline{\tilde{\mu}_i})^2} \qquad \text{(S25)}$$
$$\widehat{\text{RMSE}}(\mu) = \sqrt{\frac{1}{N_{\text{sig}}} \sum_{i=1}^{N_{\text{sig}}} (\tilde{\mu}_i - \mu)^2}$$

The values obtained for conditional bias, standard error and RMSE are plotted against the true standardized effect sizes, μ in S29 Fig below, represented by black circles. In a similar manner, the conditional bias, standard error and RMSE were obtained with respect to the naïve approach using the above equations but in which $\tilde{\mu}_i$ is replaced with z_i. These values are depicted with blue squares in S29 Fig.

For true standardized effect sizes between 4 and 8, the conditional likelihood RMSE is clearly consistently greater than the naïve approach, as anticipated from our observations in the simulation study. However, the RMSE does decrease as the true standardized effect size increases and moves away from the significance threshold. The standard error can be seen to follows a similar pattern. In addition, even though the conditional likelihood bias is generally smaller than that of the naïve approach, overcorrection is evident and seems to be most severe for true standardized effect sizes less than the 5×10^{-8} threshold with bias values close to -1. S29 Fig thus demonstrates that overcorrection can also contribute to large RMSE values for the conditional likelihood method, especially when this RMSE is computed over many significant SNPs close to the significance threshold.

Note that we are only able to perform an analysis such as the one described for the conditional likelihood methods as correction is performed to each SNP separately, independently of estimated associations of other SNPs.

S29 Fig. Conditional bias, standard error and RMSE, conditional on selection, for various true standardized effect sizes for the conditional likelihood method and the naïve approach. True standardized effect sizes, μ (x-axis) are plotted against conditional bias, standard error and RMSE (y-axis), conditional on selection at the genome-wide significance threshold (5×10^{-8}) in plots (A), (B) and (C), respectively. Values of bias, standard error and RMSE computed using the estimated effect sizes obtained after application of the conditional likelihood method are represented by black circles while the blue squares denote values calculated using the naïve estimated effect sizes. The red dotted line represents the genome-wide significance threshold of 5×10^{-8}.

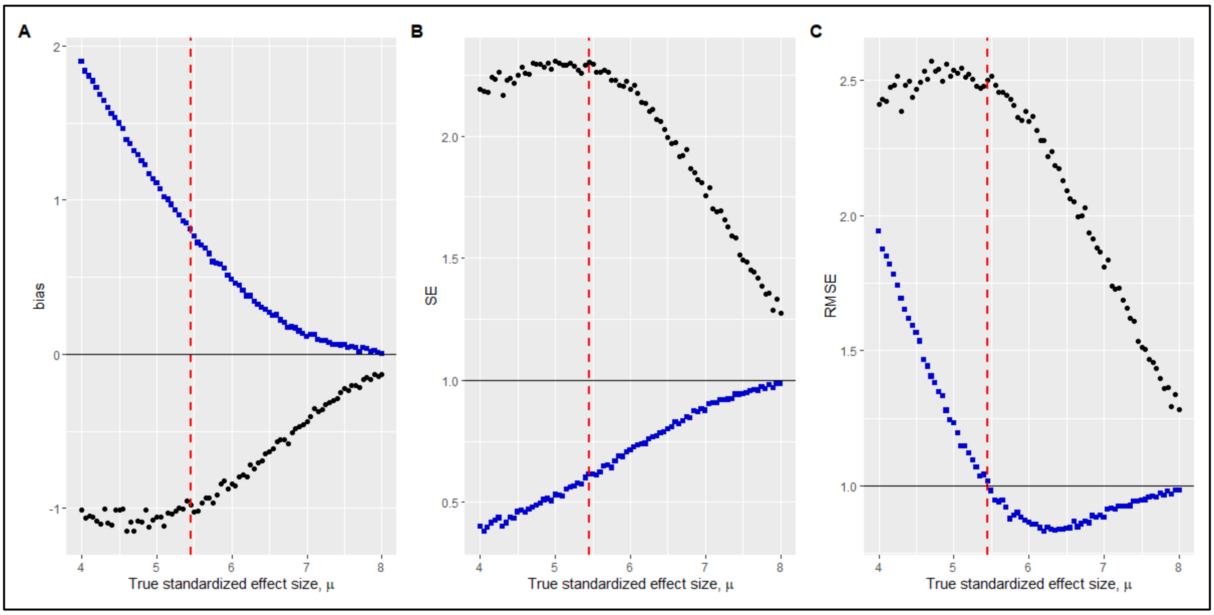

References

1. Efron B. Tweedie's formula and selection bias. J Am Stat Assoc. 2011;106(496):1602-14.

2. Wood SN. Fast stable restricted maximum likelihood and marginal likelihood estimation of semiparametric generalized linear models. J R Stat Soc Series B Stat Methodol. 2011;73(1):3-36.

3. Pya N, Wood SN. Shape constrained additive models. Stat Comput. 2015;25(3):543-59.

4. Purcell S, Neale B, Todd-Brown K, Thomas L, Ferreira MA, Bender D, et al. PLINK: a tool set for whole-genome association and population-based linkage analyses. Am J Hum Genet. 2007;81(3):559-75.

5. Lloyd-Jones LR, Zeng J, Sidorenko J, Yengo L, Moser G, Kemper KE, et al. Improved polygenic prediction by Bayesian multiple regression on summary statistics. Nat Commun. 2019;10:5086.

6. Bosch E, Laayouni H, Morcillo-Suarez C, Casals F, Moreno-Estrada A, Ferrer-Admetlla A, et al. Decay of linkage disequilibrium within genes across HGDP-CEPH human samples: most population isolates do not show increased LD. BMC Genomics. 2009;10(1):1-9.

7. Yi N, Zhi D. Bayesian analysis of rare variants in genetic association studies. Genet Epidemiol. 2011;35(1):57-69.

8. Turner SD. qqman: an R package for visualizing GWAS results using Q-Q and manhattan plots. J Open Source Softw. 2018;3(25):731.
